# Development of Nonclassical Photoprecursors for Rh_2_ Nitrenes

**DOI:** 10.1021/acs.inorgchem.3c01820

**Published:** 2023-07-27

**Authors:** Arpan Paikar, Gerard P. Van Trieste, Anuvab Das, Chih-Wei Wang, Tiffany E. Sill, Nattamai Bhuvanesh, David C. Powers

**Affiliations:** Department of Chemistry, Texas A&M University, College Station, Texas 77843, United States

## Abstract

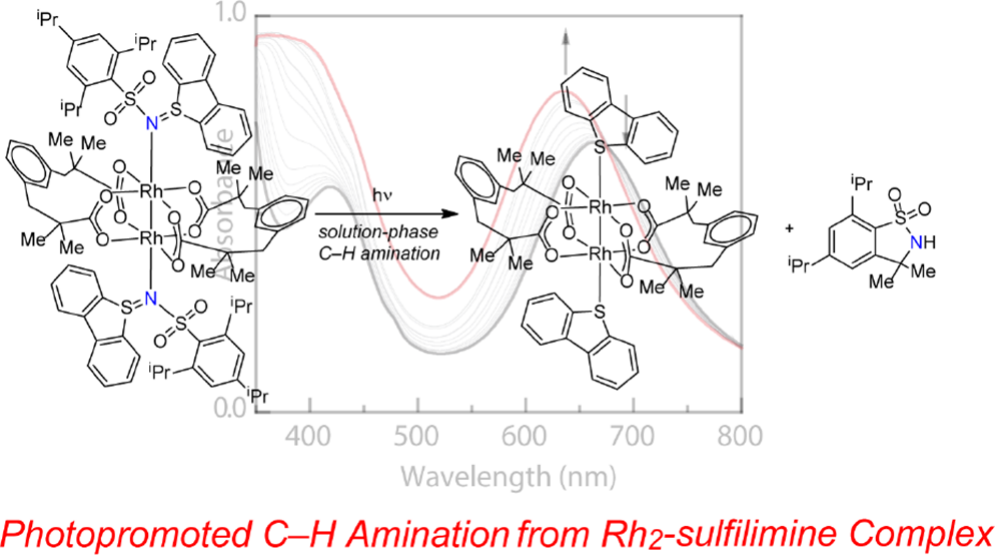

Characterization of reactive intermediates in C–H functionalization is challenging due to the fleeting lifetimes of these species. Synthetic photochemistry provides a strategy to generate post-turnover-limiting-step intermediates in catalysis under cryogenic conditions that enable characterization. We have a long-standing interest in the structure and reactivity of Rh_2_ nitrene intermediates, which are implicated as transient intermediates in Rh_2_-catalyzed C–H amination. Previously, we demonstrated that Rh_2_ complexes bearing organic azide ligands can serve as solid-state and *in crystallo* photoprecursors in the synthesis of transient Rh_2_ nitrenoids. Complementary solution-phase experiments have not been available due to the weak binding of most organic azides to Rh_2_ complexes. Furthermore, the volatility of the N_2_ that is evolved during *in crystallo* nitrene synthesis from these precursors has prevented the *in crystallo* observation of C–H functionalization from lattice-confined nitrenes. Motivated by these challenges, here we describe the synthesis and photochemistry of nonclassical nitrene precursors based on sulfilimine ligands. Sulfilimines bind to Rh_2_ carboxylate complexes more tightly than the corresponding azides, which has enabled the full solid-state and solution-phase characterization of these new complexes. The higher binding affinity of sulfilimine ligands as compared with organic azides has enabled both solution-phase and solid-state nitrene photochemistry. Cryogenic photochemical studies of Rh_2_ sulfilimine complexes confined within polystyrene thin films demonstrate that sulfilimine photochemistry can be accomplished at low temperature but that C–H amination is rapid at temperatures compatible with N=S photoactivation. The potential of these structures to serve as platforms for multistep *in crystallo* cascades is discussed.

## Abstract 2

The synthesis of dibenzothiophene-derived sulfilimine ligands and their coordination with Rh_2_esp_2_ is described. The coordination of the sulfilimine ligands with Rh_2_(II,II) complexes is characterized by UV−vis, IR, NMR spectroscopy, and X-ray crystallography. Photochemically promoted nitrogen group transfer (NGT) chemistry is observed in both solution-phase and solid-state photolyses, demonstrating the potential of sulfilimine ligands as nonclassical nitrene photoprecursors.

## Introduction

Rh_2_-catalyzed C–H amination reactions have emerged as among the most efficient and functional group-tolerant methods to convert readily available C–H bonds to C–N bonds and have been demonstrated in the context of complex molecule synthesis.^1−10^ Motivated both by a fundamental interest in the mechanistic details of C–H amination processes and by a practical interest in extending the scope of Rh_2_-catalyzed amination to stronger C–H bonds, unique chemoselectivities, and intermolecular bond constructions, a series of experimental and computational investigations have been pursued to elucidate the key mechanistic details of C–H cleavage and nitrogen-group transfer (NGT).^11−17^ In general, Rh_2_-catalyzed NGT reactions proceed through turnover-limiting nitrene transfer from iminoiodinane, organic azide, or hydroxylamine derivatives (i.e., classical nitrene precursors) to the Rh_2_ catalyst to generate a transient Rh_2_ nitrene intermediate.^11,12^ Subsequent NGT to the C–H bond affords amines and regenerates the Rh_2_[II,II] catalyst. Because the Rh_2_ nitrene intermediates that engage in C–H bond cleavage and NGT chemistry is post-turnover limiting step, these structures are not observable *in operando* but are instead inferred based on kinetics experiments and computational studies.^11,15^

**Figure 1:**
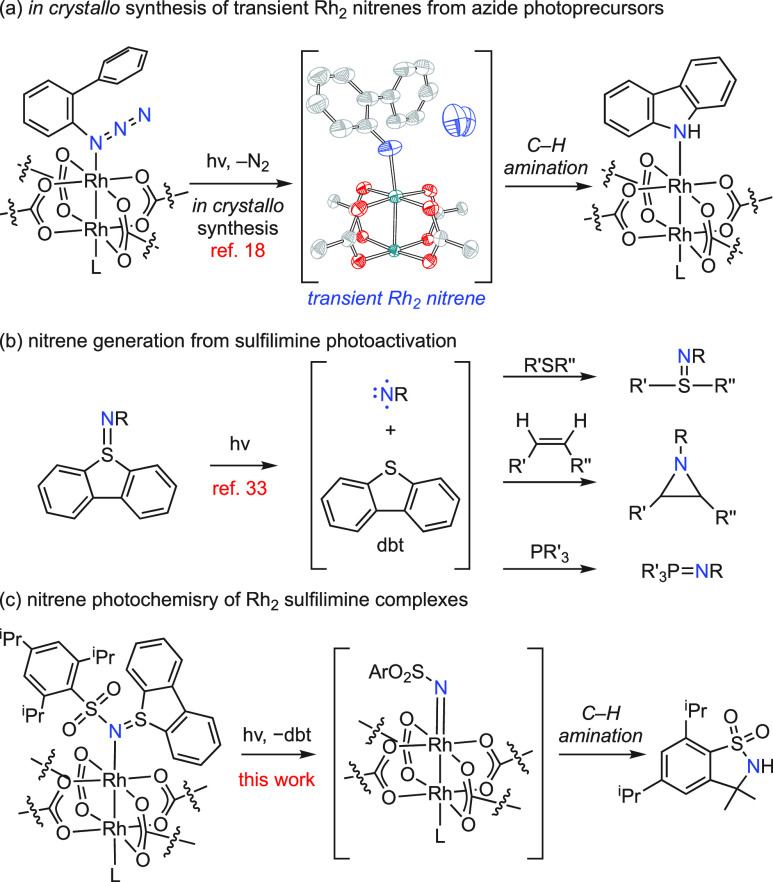
(a) *In crystallo* photogeneration and characterization of transient Rh_2_ nitrene from an organic azide photoprecursor. (b) Photoactivation of dibenzosulfilimines affords reactive nitrene fragments with concurrent extrusion of dibenzothiophene. (c) Here, we demonstrate the nitrene photochemistry of Rh_2_ sulfilimine complexes.

We have been interested in advancing *in crystallo* photochemistry as a method to prepare and characterize reactive intermediates within single crystal environments (Figure 1a).^18−22^ These studies are predicated on the photolysis of single-crystal samples of molecular precursors to unveil the reactive species of interest.^23−30^ The combination of lattice confinement and cryogenic temperatures extends the lifetimes of photogenerated intermediates to enable characterization by using diffraction-based methods. In the context of Rh_2_ nitrenes, we demonstrated that organic azide ligands represent appropriate photoprecursors to the Rh_2_ nitrenes (via solid-state photoextrusion of N_2_).^19,21^ While these studies enabled observation of Rh_2_ nitrenes, (1) N_2_ mobility within the single crystal resulted in loss of crystallinity during thermal annealing experiments designed to promote *in crystallo* NGT at the lattice-confined Rh_2_ nitrene, and (2) lability of organic azide ligands in solution prevented complementary spectroscopic experiments.^18^

We sought to overcome these challenges by designing new photolabile nitrene precursors (i.e., nonclassical nitrene precursors).^20^ We envisioned that access to more strongly binding ligands could facilitate solution-phase photochemical studies and that photoprecursors based on nongaseous leaving groups may enable multistep *in crystallo* cascades to be accomplished without loss of crystallinity. Motivated by reports of S–N bond photoactivation from dibenzothiophene sulfilimines to unveil reactive nitrene fragments and dibenzothiophene (dbt, Figure 1b),^31−34^ here, we describe the synthesis, characterization, and nitrene photochemistry of a family of Rh_2_ sulfilimine complexes (Figure 1c). Ligand titration experiments confirm that sulfilimine ligands bind Rh_2_ more tightly than do the corresponding azide ligands, which enables solution-phase characterization of these precursors. Photolysis, in either the solution phase or the solid state, results in NGT chemistry. Immobilization within polystyrene thin films enabled cryogenic photochemical experiments to be carried out, which revealed that NGT is fast relative to N–S photoactivation, even at cryogenic temperatures. The results presented demonstrate a strategy in the design of nitrene photoprecursors that enables solution-phase and solid-state experiments to be pursued.

## Results and Discussion

### Synthesis and Characterization of Rh_2_ Sulfilimine Complexes

A small family of dibenzosulfilimine ligands (**6a**–**6e**) was prepared by treatment of dibenzo[*b*,*d*]thiophene 5-oxide (**3a**) or 4,6-dimethylated dibenzo[*b*,*d*]thiophene-5-oxide (**3b**) with the appropriate aryl sulfonamide in the presence of trifluoroacetic anhydride (TFAA, eq 1).^33^ This suite of sulfilimines was selected because photochemically promoted dibenzothiophene extrusion from **9a**–**9e** would result in differently substituted *N*-arylsulfonyl nitrenes and *N*-substitution has been shown to have a marked effect on nitrene transfer reactivity.^31−33^ Photochemically promoted dibenzothiophene extrusion from **9d** would result in an *N*-arylsulfonyl nitrene featuring pendant methine C–H bonds, which represent potential sites for intramolecular C–H amination.^35^
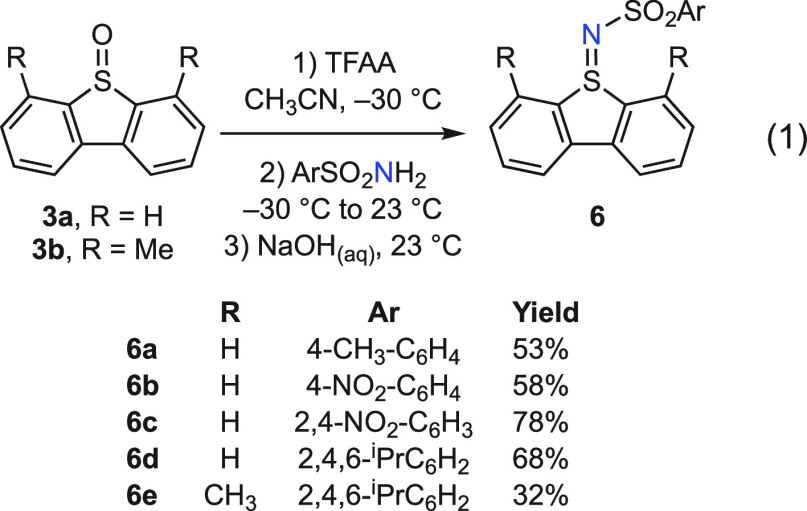
1

**Figure 2:**
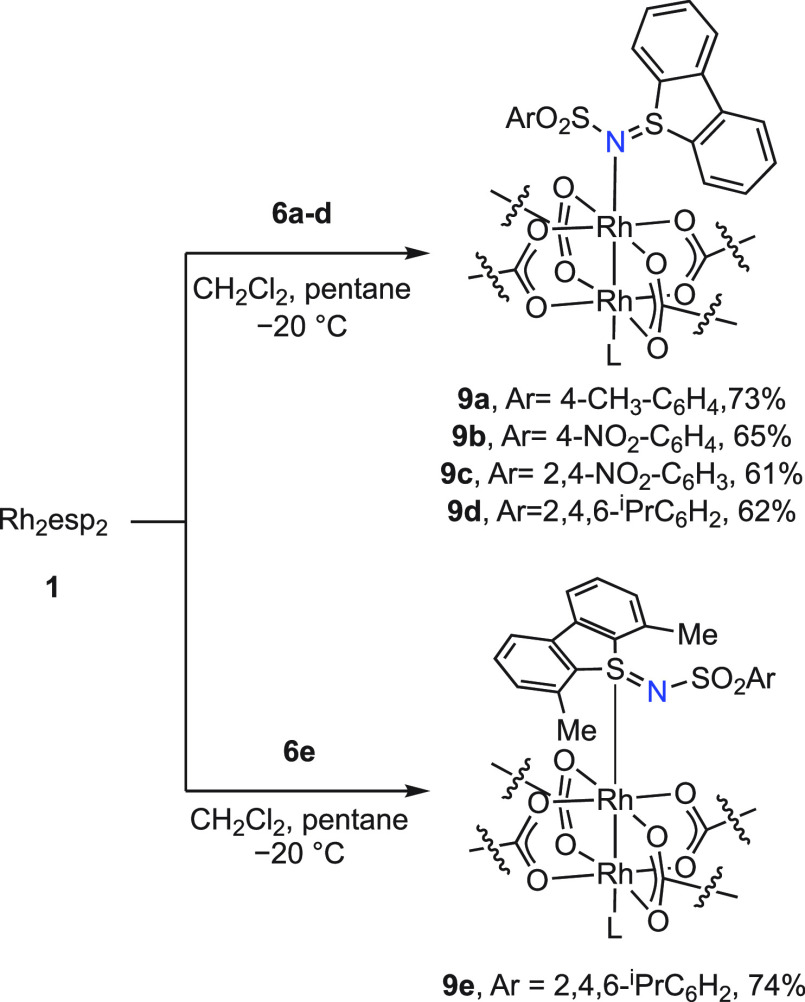
Synthesis of *N*-coordinated *bis*-sulfilimine Rh_2_ complexes (**9a**–**9d**), and *S*-coordinated *bis*-sulfilimine Rh_2_ complex **9e**, which were isolated by crystallization.

Complexation of these sulfilimine ligands with Rh_2_esp_2_ was pursued because (1) Rh_2_esp_2_ is commonly employed as a catalyst in nitrene transfer chemistry^1,2,5,7,8,13,14,16,17,36,37^ and (2) our previous *in crystallo* studies employed this complex.^18 ,21^ Treatment of Rh_2_esp_2_ with 2 equiv of sulfilimine ligands **6a**–**6d** resulted in the formation of 2:1 complexes **9a**–**9d**, which feature a *N*-coordinated sulfilimine ligand on both axial sites of the Rh_2_ core (Figure 2). In contrast, **6e** forms an *S*-coordinated 2:1 complex with the Rh_2_(II,II) core (**9e**). ^1^H NMR analysis of a 2:1 mixture of Rh_2_esp_2_ and **6a**–**6e** features diamagnetically shifted peaks that are integrated, consistent with the assigned structure. Complexes **9a**–**9e** are thermally stable compounds, which based on ^1^H NMR studies persist in solution for hours to days in the absence of light.

UV–vis spectroscopy was utilized to evaluate the donicity of the sulfilimine ligands.^21^ A CH_2_Cl_2_ solution of Rh_2_esp_2_ displays an absorbance at 425 nm, which has been ascribed to the Rh–Rh π* → Rh–O σ* transition, and a lower energy transition at 665 nm, which has been ascribed to the π* → σ* HOMO–LUMO transition.^38^ In comparison, while the higher energy feature does not vary significantly as a function of the sulfilimine structure (**9a**–**9d**), the lower energy feature of these complexes is blue-shifted to 639, 641, 655, and 662 nm, respectively. The lower energy feature is sensitive to the donicity of the axial ligand as this donor perturbs the π* → σ* transition:^39^ In general, the higher the donicity of the axial ligand, the larger the HOMO/LUMO gap is, because the σ* parameter would be raised by the stronger donation of the axial ligand and, consequently, the lower energy band will be more blue-shifted.^38^ Based on this argument, **6a** and **6b** are stronger donors than **6c** and **6d** to the Rh_2_(II,II) core.

Sulfilimine complexes **9** were further characterized in the solid state by diffuse reflectance and IR spectroscopies. Diffuse reflectance UV–vis spectra were obtained for crystalline samples of compounds **1**, **9b**, and **9d** (see Figure S1 in the Supporting Information). Similar to the solution-phase spectra of these compounds, the low-energy feature of Rh_2_esp_2_ is blue-shifted upon coordination of sulfilimines. When compared with the solution-state spectra, the solid-state spectra indicate that the Rh–Rh π* → σ* transition is sensitive to solvent polarity: In the solid state, the low energy bands of **9b** and **9d** are blue-shifted to 582 and 632 nm, respectively, as compared to 641 nm (**9b**) and 662 nm (**9d**) in solution.^40^ The collected IR spectra indicated that the S=N stretching frequency is significantly red-shifted upon binding to Rh_2_esp_2_: For **6a**, ν_N=S_ = 939 cm^–1^, while, for **9a**, ν_N=S_ = 929 cm^–1^ (see Figure S2 in the Supporting Information for IR spectral comparison of sulfilimines and the corresponding Rh_2_ complexes).

**Figure 3:**
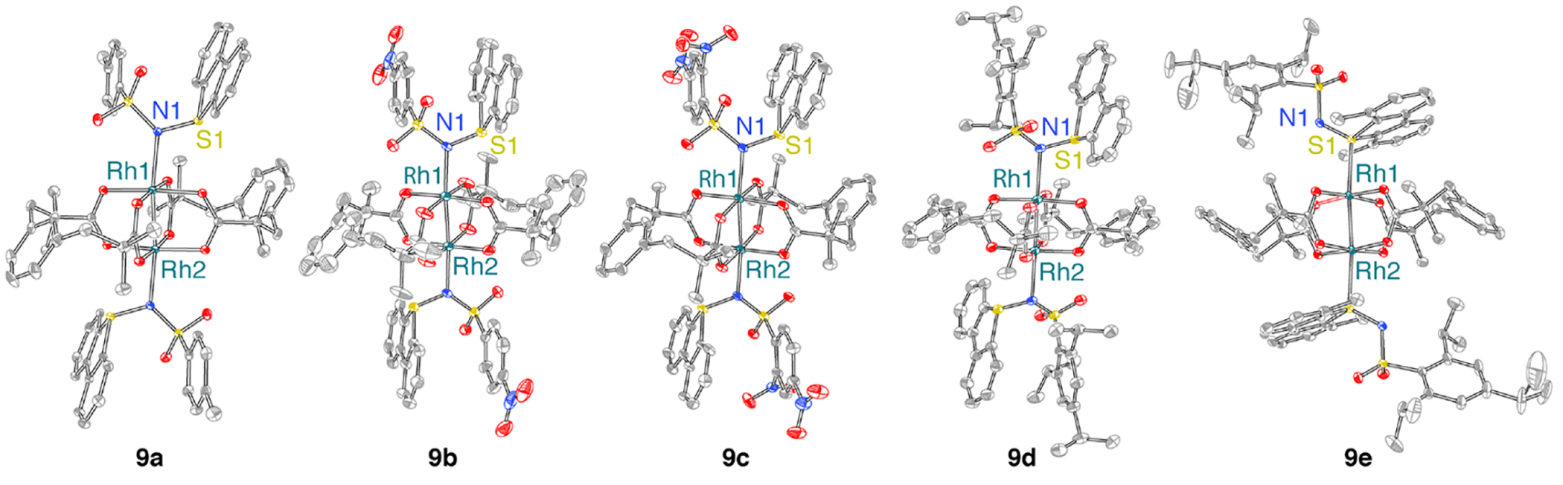
Displacement ellipsoid plots of *N*-coordinated Rh_2_ sulfilimine complexes **9a**–**9d** and *S*-coordinated **9e** plotted at 50% probability. H atoms and solvents are removed for clarity. The crystalline sample used in this diffraction experiment was obtained from a concentrated CH_2_Cl_2_ solution layered with pentane at −20 °C. Selected metrical parameters: for **9a**, Rh(1)–N(1) = 2.2992(2) Å and Rh(1)–Rh(2) = 2.3893(3) Å; for **9b**. Rh(1)–N(1) = 2.297(4) Å and Rh(1)–Rh(2) = 2.3944(8) Å; for **9c**, Rh(1)–N(1) = 2.3374(1) Å and Rh(1)–Rh(2) = 2.3921(3) Å; for **9d**, Rh(1)–N(1) = 2.363(3) Å and Rh(1)–Rh(2) = 2.4026(5) Å; for **9e**, Rh(1)–S(1) = 2.5438(5) Å and Rh(1)–Rh(2) = 2.4048(3) Å.

The solid-state structures of **9a**, **9b**, **9c**, **9d**, and **9e** were obtained by single-crystal X-ray diffraction analysis, and displacement ellipsoid plots are collected in Figure 3. Refinement data are collected in Table 1. Consistent with the ^1^H NMR analysis, X-ray structures show that **9a**, **9b**, **9c**, **9d**, and **9e** are 2:1 complexes in which the sulfilimine ligands bind in the apical positions of the Rh_2_(II,II) core. The Rh(1)–N(1) distances in **9a**–**9d** are 2.2992(2) Å, 2.297(4) Å, 2.3374(1) Å, and 2.363(3) Å, respectively. The significant elongation of the Rh–N bond for compounds **9c** and **9d** may arise due to the enhanced steric profile of sulfilimines **6c** and **6d**.^38^ The apparent weaker coordination of the sulfilimine ligands in complexes **9c** and **9d** is consistent with UV vis analysis described above.^38^ In contrast to **9a**–**9d**, which feature *N*-bound sulfilimine ligands, **9e** features an *S*-bound ligand. This may arise due to differences in the spatial orientations of the aryl sulfonyl substituents and the dibenzothiophene fragment of the sulfilimine ligands: For **9a**–**9d**, the two rings are almost parallel, making the *N*-atom accessible to coordinate with the Rh_2_(II,II); in the case of **9e**, the dimethylated dibenzothiophene and the aryl sulfonyl moieties are perpendicular to each other in such a way that the *N*-atom is unable to access the Rh_2_ center.

**Table 1:** Crystal Data and Structure Refinement

	**9a**·2CH_2_Cl_2_	**9b**·CH_2_Cl_2_	**9c**·4CH_2_Cl_2_	**9d**	**9e**
formula	C_70_H_70_N_2_O_12_Rh_2_S_4_·2(CH_2_Cl_2_)	C_68_H_64_N_4_O_16_Rh_2_S_4_·CH_2_Cl_2_	C_68_H_62_N_6_O_20_Rh_2_S_4_·4(CH_2_Cl_2_)	C_86_H_102_N_2_O_12_Rh_2_S_4_	C_90_H_110_N_2_O_12_Rh_2_S_4_
temp, K	110	100	110	100	110
cryst system	orthorhombic	monoclinic	triclinic	triclinic	monoclinic
space group	*Pbca*	*C*2/*c*	*P*1̅	*P*1̅	*P*2_1_/*C*
color	green	green	green	light green	green
*a*, Å	19.1550(7)	25.393(3)	10.7438(6)	13.044(1)	15.610(1)
*b*, Å	17.2810(5)	14.204(2)	13.7006(8)	13.531(2)	11.0136(8)
*c*, Å	21.6940(6)	22.950(3)	14.487(1)	14.145(1)	26.606(2)
α, deg	90	90	102.721(2)	62.503(2)	90
β, deg	90	120.729(2)	93.557(2)	69.314(2)	95.679(2)
γ, deg	90	90	102.459(2)	72.954(2)	90
*V*, A^3^	7181.1(4)	7715.0(1)	2017.7(2)	2046.2(3)	4551.7(5)
*Z*	4	4	1	1	2
*R*_1_^a^	0.034	0.0683	0.0273	0.0453	0.0373
*wR*_2_^b^	0.0724	0.1734	0.0534	0.1098	0.0184
GOF (*F*^2^)^c^	1.062	1.177	1.058	1.052	1.110

a *R*_1_ = ∑||*F*_o_ – |*F*_c_||/∑|*F*_o_|.

b *wR*_2_ = (∑(*w*(*F*_o_^2^ – *F*_c_^2^)^2^)/∑(*w*(*F*_o_^2^)^2^))^1/2^.

c GOF = (∑*w*(*F*_o_^2^ – *F*_c_^2^)^2^/(*n* – *p*))^1/2^, where *n* is the number of data and *p* is the number of parameters refined.

### Synthesis and Characterization of Dibenzothiophene Complex **10**

**Figure 4:**
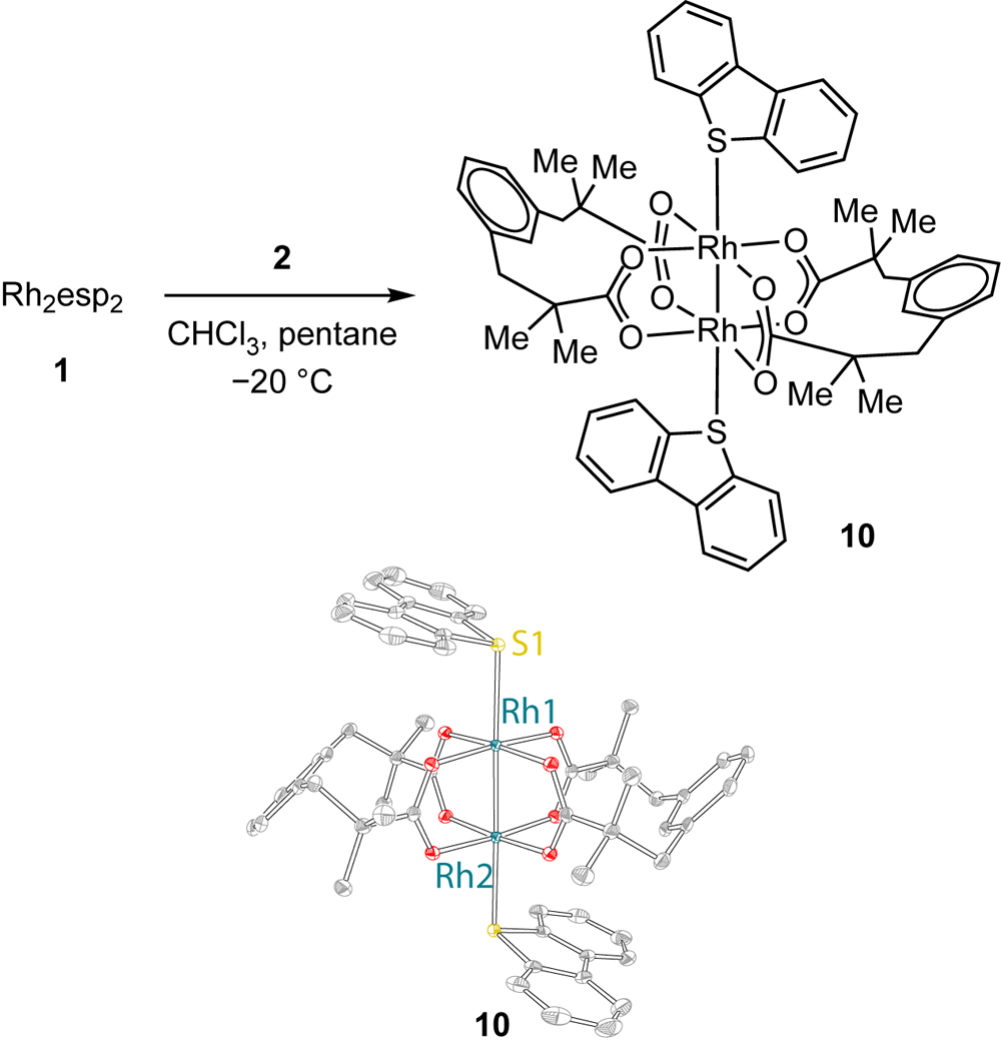
Reaction of dibenzothiophene with **1** affords Rh_2_esp_2_(dbt)_2_ (**10**). Displacement ellipsoid plots of **10** are plotted at 50% probability. H-atoms and and solvent are removed for the sake of clarity. Selected metrical parameters: for **10**, Rh(1)–S(1) = 2.5298(4) Å and Rh(1)–Rh(2) = 2.3993(3) Å.

The targeted nitrene photochemistry of Rh_2_ sulfilimine complexes would be accompanied by the evolution of dbt, which represents a potential ligand for Rh_2_. To evaluate the coordination chemistry of dibenzothiophene, we treated Rh_2_esp_2_ with 2 equiv of dibenzothiophene, which resulted in the formation of adduct **10** (Figure 4). Solution-phase UV–vis spectroscopy of **10** in dichloromethane revealed absorbances centered at 636 nm and a shoulder at 434 nm. ^1^H NMR analysis supported the formulation as a 2:1 adduct. Refinement of single-crystal X-ray diffraction data provided the structure illustrated in Figure 4.

### Ligand Binding Thermodynamics

**Figure 5:**
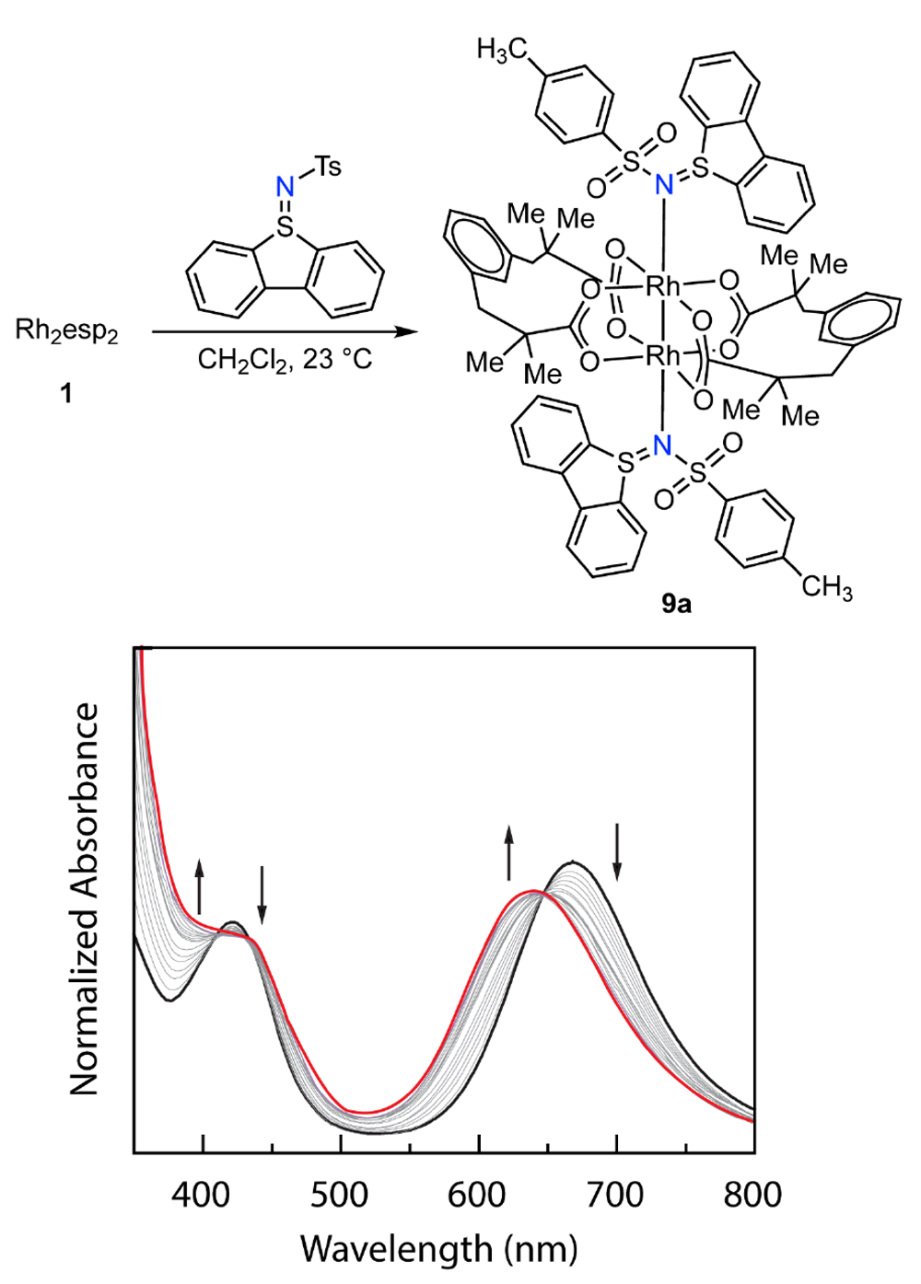
UV–vis spectra obtained during the titration of **1** with **6a**. The well-anchored isosbestic points at 434 and 639 nm indicate the absence of steady-state intermediates in the conversion of **1** (black) to **9a** (red).

To assess the binding of the new family of sulfilimines to Rh_2_, we carried out a series of UV–vis titrations of Rh_2_esp_2_ with sulfilimines **6a**–**6d**. Here, we discuss the UV–vis titration of sulfilimine **6a** with Rh_2_esp_2_ (**1**), which is representative of analogous experiments with **6b**–**6d**. The data obtained from the titration of Rh_2_esp_2_ with **6a** are illustrated in Figure 5 (see Figures S3–S6 in the Supporting Information) for spectral data corresponding to titrations of Rh_2_esp_2_ with sulfilimines **6a**–**6d**). A dichloromethane solution of **1** displays characteristic peaks centered at 665 and 425 nm (*vide supra*).^21,38^ UV–vis spectra obtained via the addition of increasing amounts of **6a** to a solution of Rh_2_esp_2_ display a blue shift of the low-energy feature from 665 nm to 639 nm. The spectral evolution ceased after the addition of 2 equiv of **6a**, consistent with conversion of Rh_2_esp_2_ to **9a** and well-anchored isosbestic points at 646 and 434 nm indicate the absence of steady-state intermediates in this conversion.

The observation that spectral evolution ceases at a 1:2 ratio of **1** and **6a** indicates tight binding of the sulfilimine ligand to the Rh_2_ core (*K*_eq_ > 10). In contrast, similar concentration-dependent UV–vis titration spectra between **1** and TsN_3_ (**8a**) continues to evolve until the addition of ∼25 equiv of **8a** (Figure S7 in the Supporting Information). Similar UV–vis analysis was also performed with NsN_3_ (**8b**) (Figure S8 in the Supporting Information) and TbsN_3_ (**8c**) (Figure S9 in the Supporting Information), which also show ∼20 equiv of organic azides are required to saturate the coordination of **1**.

While sulfilimine ligand **6a** is more tightly binding than the corresponding azide, it can be displaced by other potentially coordinating ligands, such as THF. Titration of complex **9a** with tetrahydrofuran (THF) results in conversion to Rh_2_esp_2_(THF)_2_; ∼2.5 equiv of THF was needed to completely displace the sulfilimine ligands from **9a** (see Figure S10 in the Supporting Information for similar analysis with **9a**). These data indicate that sulfilimine ligands bind more weakly than THF but more strongly than the corresponding sulfonyl azides. Furthermore, while the addition of **6b** or **6c** to a solution of **9a** did not result in a significant spectral change, the addition of **6a** or **6b** to a solution of **9c** did result in a significant blue shift, which suggests that the sulfilimine ligands are exchangeable and that the binding preferences are **6a** > **6b** > **6c** (Figure S11 in the Supporting Information).

### Solution-Phase Photochemistry

**Figure 6:**
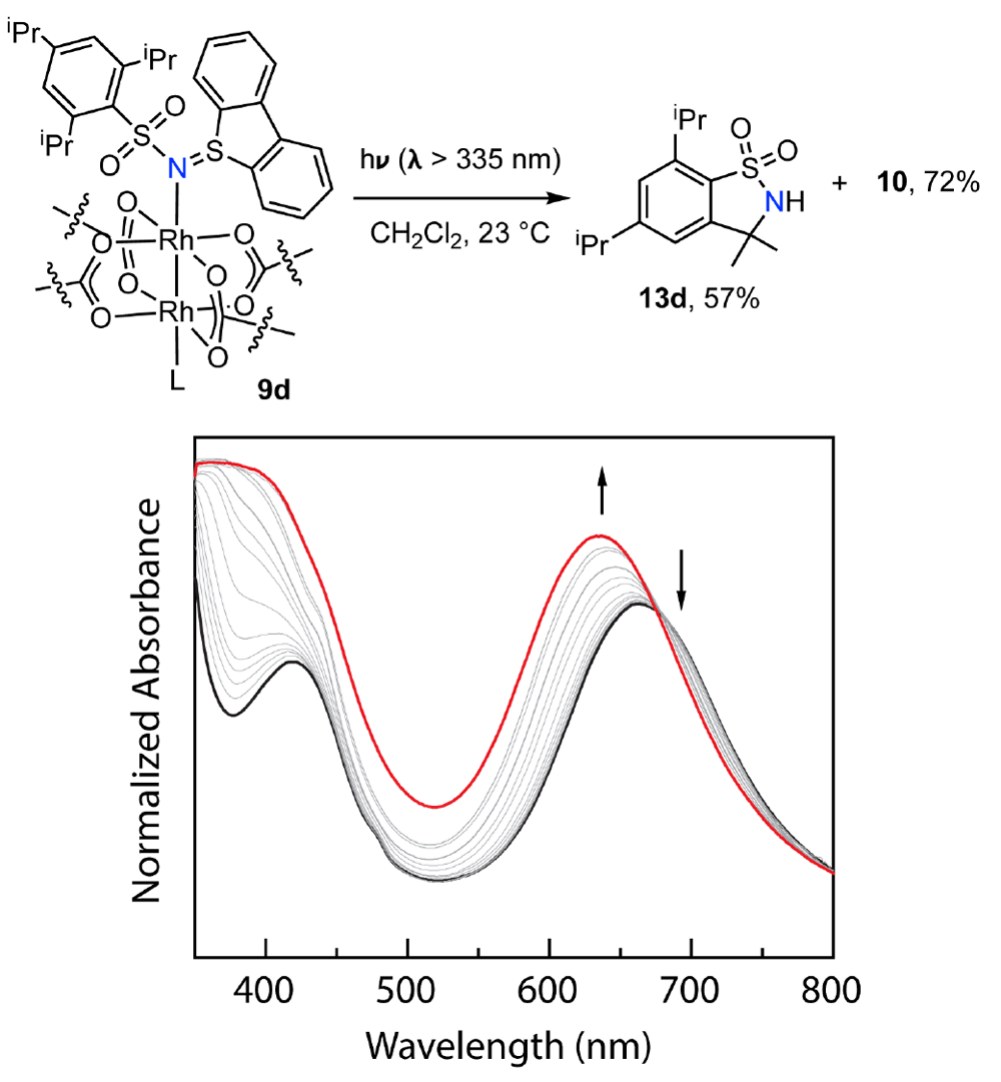
Photolysis of Rh_2_ sulfilimine **9d** results in Rh_2_esp_2_(dbt)_2_ (**10**) and heterocycle **13d**, which are the products expected of intramolecular NGT photochemistry. A well-anchored isosbestic point is observed at 675 nm for the UV–vis spectra collected during photolysis of compound **9d** in CH_2_Cl_2_ (λ > 335 nm), which indicates the lack of a steady-state intermediate in the conversion of **9d** to **10** and **13d**.

Because the sulfilimine ligands are tightly bound to Rh_2_ (*vide supra*), we are able to explore the photochemistry of the corresponding Rh_2_ complexes (i.e., **9a**–**d**) in the absence of an exogenous sulfilimine ligand. We began our investigations of the nitrene photochemistry of Rh_2_ sulfilimine complexes with complex **9d**, because we hypothesized that potential intramolecular amination would simplify the analysis of the reaction outcomes. Photolysis (λ > 335 nm) of a CH_2_Cl_2_ solution of **9d** was monitored periodically by UV–vis spectroscopy (Figure 6). The obtained spectra display a blue shift in the lowest energy absorbance, and the spectral evolution is characterized by the presence of a well-anchored isosbestic point at 675 nm, which indicates the lack of steady-state intermediates in the photochemistry of **9d**. The ultimate spectrum obtained following photolysis is superimposable with that of dibenzothiophene complex **10** (Figure S12b in the Supporting Information). The identity of the Rh-containing photoproduct (i.e., **10**) was further confirmed by crystallization and X-ray diffraction analysis of the photochemical reaction mixture. Preparative-scale photolysis of **9d** enabled isolation and characterization of the organic fragment generated to be heterocycle **13d**, which is the product expected of intramolecular C–H amination chemistry (57% yield) (for UV–vis and NMR analysis of the photolysis of compound **9d** see Figures S12 and S13 in the Supporting Information). Similarly, the photolysis of a CH_2_Cl_2_ solution of **9e** results in the formation of Rh_2_esp_2_(dmdbt)_2_ (**15**) (Figure S14 in the Supporting Information). The formation of heterocycle **13d** is evidenced by the ^1^H NMR spectra of the reaction mixture following photolysis (Figure S15 in the Supporting Information). We speculate that the nitrene photochemistry of **9e** may result from the initial *S*- to *N*-photoisomerization.

**Figure 7:**
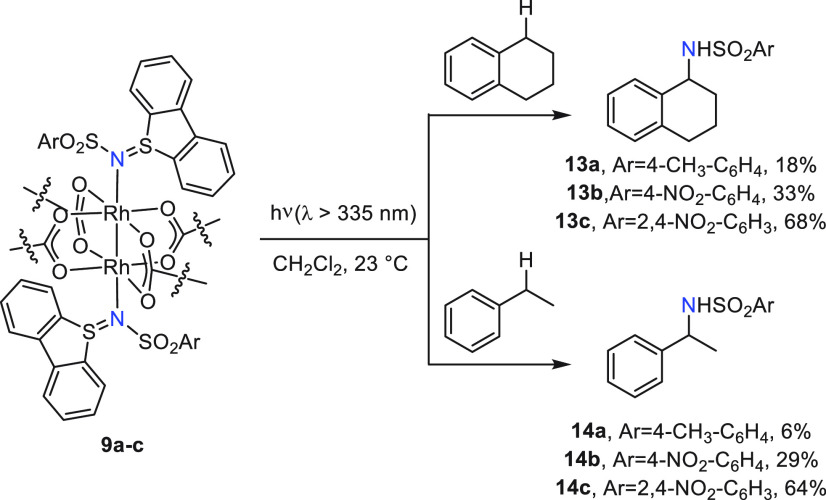
Photolysis of compounds **9a**, **9b**, and **9c** with tetralin and ethylbenzene in CH_2_Cl_2_ results in the respective intermolecular aminated products.

Photolysis of CH_2_Cl_2_ solutions of sulfilimine complexes **9a**–**9c** in the presence of appropriate nitrene traps, such as tetralin or ethylbenzene, results in the products of intermolecular nitrene transfer: Rh_2_esp_2_(dbt)_2_ (**10**) and the trap-derived benzylamine derivative (**13a**–**13c** or **14a**–**14c**) were observed by ^1^H NMR spectroscopy of crude photolysis mixtures (Figure 7). In comparison, photolysis of **6a** and **6b** under analogous conditions resulted in no amination products; photolysis of **6c** resulted in <10% yield of **13c** and trace amount of **14c**. In addition, no amination from **9a**–**9c** was obtained in the absence of light or thermolytic conditions at 40 °C (for UV–vis evolution and NMR analysis of the photolysis of compounds **9a**–**9c** with tetralin, see Figures S13–S18 in the Supporting Information).

The efficiency of intermolecular C–H amination depends intimately on the substitution of the *N*-sulfonyl substituents. The addition of electron-withdrawing aryl substituents, which results in progressively lower ligand-centered LUMOs, results in increasingly efficient amination of both tetralin and ethylbenzene. Similar substituent effects have been previously described in Rh_2_-catalyzed nitrene transfer reactions.^14^

### Solid-State Photochemistry

**Figure 8:**
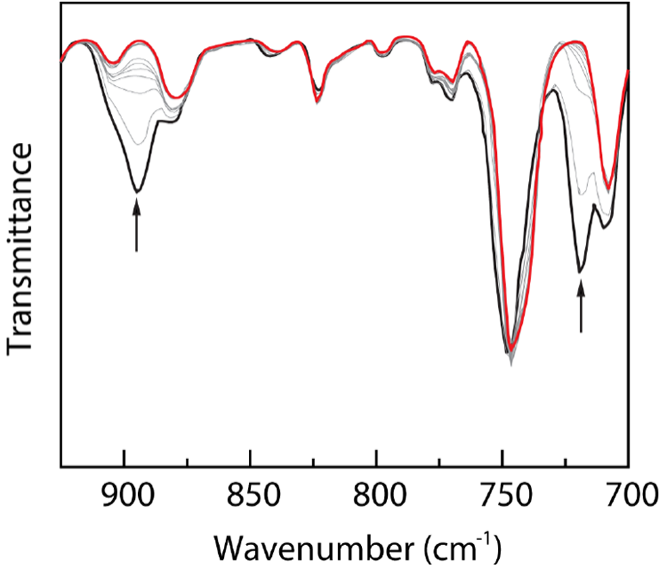
Solid-state photolysis of compound IR spectra collected during the photolysis (335 nm < λ < 610 nm) of a KBr pellet of **9d** at 23 °C from 0 min (black) to 24 h (red). The disappearance of the peaks at 896 cm^–1^ is for the N=S cleavage. The disappearance of the peak at 719 cm^–1^ and slight red shift from 752 cm^–1^ to 744 cm^–1^ depicts the conversion from **9d** to **10** during 24 h of photolysis.

We were interested in studying the solid-state photochemistry of compounds **9** as a platform to both examine chemistry in the absence of potentially reactive solvents and to use this as the basis for *in crystallo* experiments. To this end, we monitored the solid-state photolysis of **9b** and **9d** by using IR spectroscopy (Figure 8). The time-dependent IR spectra of **9d** display the depletion of the peak at 896 cm^–1^, which corresponds to the N=S stretching frequency in Rh_2_-bound sulfilimine ligands. The concurrent disappearance of the peak at 719 cm^–1^ and slight red shift of the peak at 752 to 744 cm^–1^ indicate the formation Rh_2_esp_2_(dbt)_2_ (**10**) during the solid-state photolysis (Figures S22 and S23 in the Supporting Information). Together, these observations suggest that the solid-state photochemistry of **9d** is analogous to the solution-phase photochemistry. For detailed IR analysis of the solid-state photochemistry of compound **9b**, see Figures S24 and S25 in the Supporting Information. Solid-state photolysis of **9e** also proceeds analogously to the solution-phase experiments (i.e., the formation of **13d** and **15**; see Figures S26 and S27 in the Supporting Information for relevant spectral data).

### Photocrystallography

We have previously demonstrated *in crystallo* photosynthesis of transient Rh_2_ nitrenes using Rh_2_ complexes featuring Rh_2_-bound organic azide ligands.^18,21^ While these experiments provided evidence for the formation of triplet nitrene adducts of Rh_2_, the synthesis of the nitrene was accompanied by the extrusion of N_2_. At cryogenic temperatures, N_2_ could be localized in electron density maps. Warming the single-crystalline samples in order to promote C–H amination resulted in a rapid loss of crystallinity, presumably due to increased mobility of intracrystalline N_2_.^18^ Based on the fact that dbt is solid at ambient temperature, we hypothesized that dbt photoextrusion could result in a crystalline nitrene that could engage in subsequent single-crystal to single-crystal C–H amination. To this end, we examined the photolysis (λ = 365 nm) of single crystals of complexes **9a**–**9d** by X-ray diffraction. Photolysis was carried out at 100 K and diffraction data were collected with 50 keV synchrotron radiation. Unfortunately, in no case was significant single-crystal photochemistry observed. We speculate that in the van der Waals crystals used in these experiments, photogenerated dbt was not sufficiently mobile to migrate from the reactive nitrene, thus preventing productive N=S cleavage.

### Polymer Thin-Film Photochemistry

While the sulfilimine ligands utilized to prepare adducts **9a**–**9d** are more tightly binding than the corresponding azide ligands, displacement by potentially coordinating solvents (i.e., THF) represents a challenge to cryogenic photochemical experiments aimed at the spectroscopic characterization of the transient nitrene intermediates implicated above. For example, common glassy solvents such as 2-methyltetrahydrofuran displace the sulfilimine ligands of **9**. Furthermore, complexes **9** are poorly soluble in potential noncoordinating glassy solvents, such as 2,2-dimethylbutane/*tert*-butylbenzene mixtures.^41^

**Figure 9:**
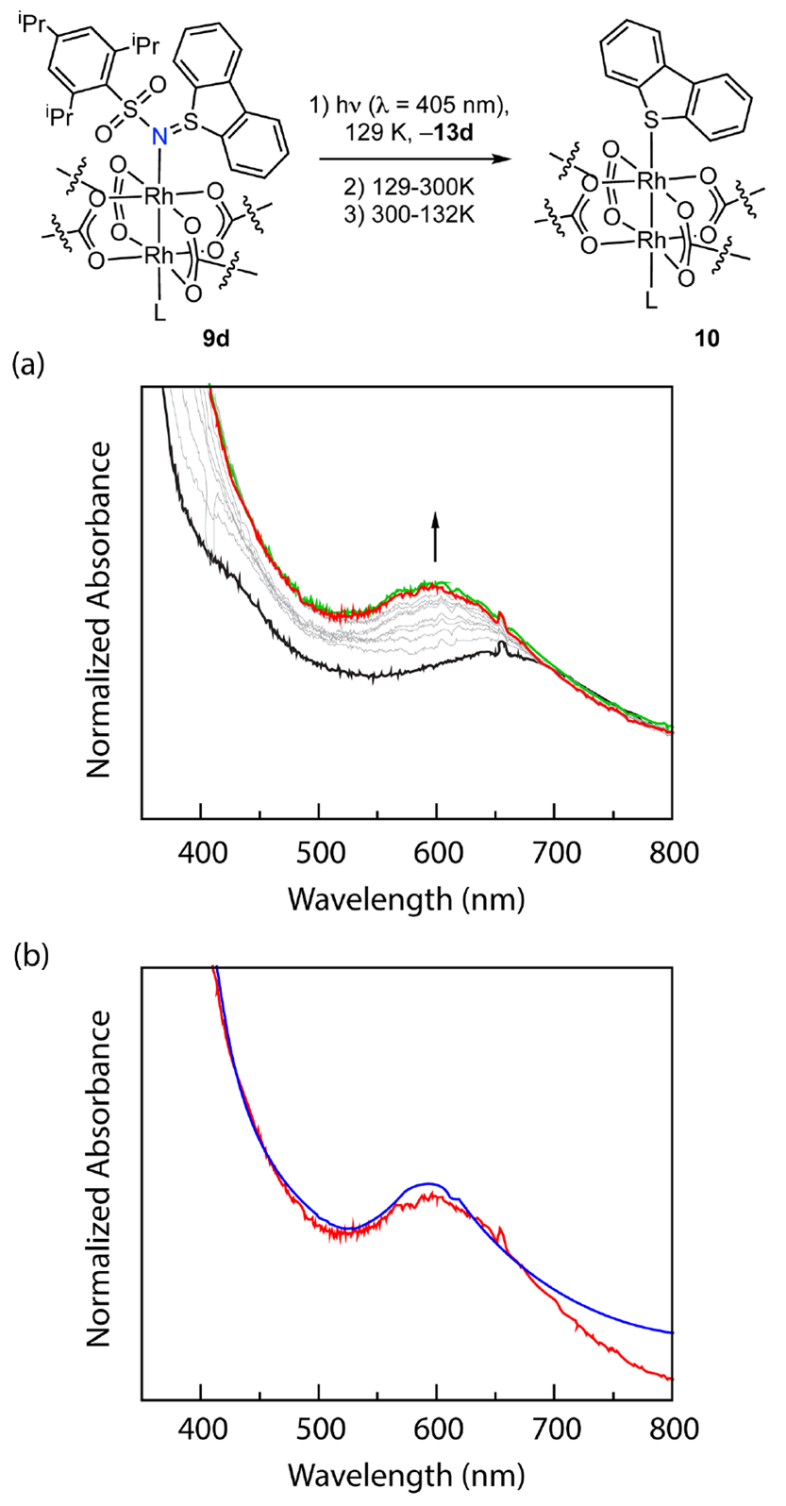
Solid-state cryogenic photolysis of compound **9d** in a polystyrene film matrix. (a) Solid-state UV–vis spectra periodically collected during the photolysis over 4 h at 129 K where the colors black, red, and green represent UV–vis spectra at *t* = 0, 4.5 h, and the end of the thaw-freeze cycle, respectively. (b) Overlay of the UV–vis spectrum of independently prepared **10** (blue) and the thermally annealed spectrum (red) indicates the formation of **10** during the cryogenic photolysis of **9d**.

To circumvent these challenges, we pursued low-temperature photochemical experiments using polymer thin films impregnated with Rh_2_ sulfilimine complexes (Figure 9).^41^ To this end, we prepared a 4.27 mM solution of **9d** in 1,2-dichloroethane containing 2 wt % polystyrene (*M*_w_ = 350 000). We dropcast a thin film on a sapphire slide and removed residual 1,2-dichloroethane *in vacuo* (for comparison between the solution-phase spectrum and thin-film spectrum of compound **9d** see Figure S28b in the Supporting Information). Photolysis (λ = 405 nm) of this film at 129 K for 4 h resulted in a blue-shift of the low-energy spectral feature of **9d** (Figure 9). Thermal annealing (300 K) followed by recooling to 129 K did not result in further spectral evolution. Furthermore, the spectrum following photolysis and thermal annealing (300 K) overlays with that of complex **10** (recorded as a polystyrene thin film). These observations indicate that C–H amination of the proximal C–H bond takes place even under cryogenic conditions. Photolysis at lower temperatures (i.e., 77 K) did not result in appreciable spectral evolution on experimentally tractable time scales.

The observation of facile intramolecular C–H amination for a thin film of **9d** but no *in crystallo* photoconversion suggests that dibenzothiophene photoextrusion is likely reversible below temperatures that are compatible with C–H amination. Poor solubility in polymer thin films prevented analogous experiments with sulfilimine complexes **9a**–**9c**.

## Concluding Remarks

Synthetic nitrene photochemistry requires access to the appropriate photoprecursor molecules. Many of the commonly encountered reagents in nitrene transfer catalysts, such as organic azides and iminoiodinanes, are either weakly binding ligands to many transition metals or kinetically labile toward nitrene transfer. As a result, access to solution-stable metal nitrene photoprecursors is limited, which prevents the general application of time-resolved solution-phase methods or solid-state matrix isolation techniques to the synthesis and characterization of these species.

Here, we have introduced sulfilimine ligands as photoprecursors to reactive metal nitrene intermediates. Specifically, we reported the coordination chemistry of a small family of sulfilimine ligands with Rh_2_esp_2_. UV–vis, NMR spectroscopy, and SCXRD data indicate 2:1 coordination of these ligands with Rh_2_(II,II) core. Concentration-dependent UV–vis titration revealed that that the sulfilimine ligands have higher binding affinity, compared to the corresponding organic azides. Both intermolecular and intramolecular C–H amination was achieved in the solution phase via the photoextrusion of the dibenzothiophene (dbt) moiety from the complexes.

Demonstration of solution-phase nitrene photochemistry presaged examination of the solid-state photolysis of these complexes. In a KBr pellet, dbt photoextrusion and ultimate formation of Rh_2_ dbt adducts (i.e., **10**) was observed. C–H amination products were obtained as well demonstrating the homology of solution-phase and solid-state processes. Cyogenic photolysis of these compounds in polystyrene thin film matrix revealed that C–H amination is fast, relative to dbt photoextrusion, which prevented direct observation of incipient nitrene intermediates.

Together, the results of solution-phase and solid-state photochemical experiments demonstrated the ability to obtain nitrene photochemistry from sulfilimine precursors. These results provide the foundation for the application of sulfilimine ligands, which balance ground-state binding thermodynamics with photolability, in the photosynthesis of metal nitrenes of interest to catalysis.

## Supporting Information Available

Experimental procedures and spectroscopic data (PDF)
