# Supplementary material for: Development of Nonclassical Photoprecursors for Rh2 Nitrenes
Source: Inorg Chem. 2023 Jul 27;62(31):12557–64. doi: 10.1021/acs.inorgchem.3c01820 (PMC10862545; doi:10.1021/acs.inorgchem.3c01820)
Supplement: Supplementary file 1 — ic3c01820_si_001.pdf [file ic3c01820_si_001.pdf]

Supporting Information

Development of Non-Classical Photoprecursors for Rh_2 Nitrenes

Arpan Paikar, Gerard P. Van Trieste III, Anuvab Das, Chih-Wei Wang, Tiffany E. Sill, Nattamai Bhuvanesh, and David C. Powers*

Department of Chemistry, Texas A&M University, College Station, TX 77843, United States.

Table of Contents

A.	General Considerations	S3
B.	Synthesis and Characterization	S6
C.	Supporting Data	S20
D.	Ligand Binding Titration	S22
E.	Ligand Exchange Experiment	S38
F.	Solution-Phase Photolysis Experiments	S39
G.	Solid-State Photolysis Experiments	S51
H.	Thin-Film Photochemistry	S57
I.	X-ray Diffraction Data	S59
J.	NMR Spectra	S71
K.	References	S97

A. General Considerations

Materials and Methods. Unless otherwise noted, all the chemicals and solvents (ACS reagent grade) were used as received. 4-Methylbenzenesulfonyl chloride was purchased from Alfa Asear. *N*,*N*,*N′*,*N′*-Tetramethylethylenediamine (TMEDA) was obtained from Strem Chemicals. Dibenzo[*b,d*]thiophene, sodium azide was purchased from BeanTown Chemical. *p*-Nitrobenzenesulfonamide and triisopropyl benzenesulfonyl chloride were purchased from Acros Organic. Methyl iodide, diethyl ether, tetralin, *m*-chloroperbenzoic acid, trifluoroacetic anhydride, *n*-butyllithium *p*-toluenesulfonamide, *meta*-chloroperoxybenzoic acid (77% *m*-CPBA), and polystyrene beads (M_w = 350000) were purchased from Sigma Aldrich. Silica gel (0.06–0.20 mm, 60 Å for column chromatography) was obtained from Acros Organics and chlorobenzene was obtained from TCI America. Dirhodium tetraacetate was purchased from Pressure Chemical Company. 2,4-Dinitrobenzene sulfonyl chloride from Thermoscientific. 1,2-Dichloroethane, $NaHCO_3$, $(NH_4)_2CO_3$, $MgSO_4$, and Na_2SO_4 were purchased from Oakwood Chemicals. NH_4OH (28-30%), and HCl (36.5-38%) were obtained from VWR chemicals. Acetonitrile, acetone, hexanes, chloroform, ethyl acetate, and dichloromethane were purchased from Fischer Chemicals. Ethylbenzene was purchased from Tokyo Chemical Company. Sapphire substrate double-sided polished slides (12.7 × 12.7 × 0.5 mm, c plane) were purchased from Advalue Technology. Tetralin, ethyl benzene, 1,2-dichloroethane, TMEDA, chloroform, and hexanes were distilled according to literature methods.[1] N_2 was purchased from Airgas. NMR solvents were purchased from Cambridge Isotope Laboratories and were used as received. All reactions were carried out under an ambient atmosphere unless otherwise noted. Anhydrous dichloromethane was obtained from a drying column and stored over activated molecular sieves.[2] Rh_2esp_2 (**1**) was prepared according to the literature method.[3] 4-Methylbenzenesulfonyl, 4-nitrobenzenesulfonyl, 2,4-dinitrobenzenesulfonyl, 2,4,6-triisopropylbenzenesulfonyl, and 4,6-dimethyldibenzo[b,d]thiophene are abbreviated as Ts-, Ns-, Dns-, Tbs-, and dmdbt, respectively.

Characterization Details. NMR spectra were recorded on Bruker Avance NEO 400 NMR spectrometer and were referenced against solvent signals:[4] $CDCl_3$ (7.26 ppm, ^{1}H; 77.16 ppm, ^{13}C), CD_2Cl_2 (5.32 ppm, 1H; 53.84 ppm, ^{13}C), and $(CD_3)_2SO$ (2.50 ppm, ^{1}H; 39.52 ppm for ^{13}C). ^{1}H NMR data are reported as follows: chemical shift (δ, ppm), multiplicity (s (singlet), d (doublet), t (triplet), dd (doublet of doublets), td (triplet of doublets), h (heptet), dq (doublet of quartets), m (multiplet), br (broad), integration. Solution-phase UV-vis spectra were recorded on a Shimadzu 2501PC spectrometer with DH UV-vis-NIR light source (190–1100 nm). Solution-phase spectra were blanked against the appropriate solvent. Solid-state UV-vis spectra were recorded on a Hitachi U-4100 spectrometer with DH UV-vis-NIR light source (175–2600 nm) and blanked against the appropriate quartz slide. IR spectra were recorded on a Shimadzu FTIR/IRAffinity-1 spectrometer, were blanked against KBr, and were determined as the average of 64 scans. ATR-IR data also collected in the same instrument. IR data are reported as follows: wavenumber (cm^{-1}), peak intensity (s, strong; m, medium; w, weak). Mass spectrometry data were recorded on either

Orbitrap Fusion™ Tribrid™ Mass Spectrometer or Q Exactive™ Focus Hybrid Quadrupole - Orbitrap™ mass spectrometer.

X-Ray Diffraction Details Experimental details of crystallization are included in the synthetic procedures for the relevant compounds. A Bruker Quest (PHOTON III) X-ray (three-circle) diffractometer was used for crystal screening, unit cell determination, and data collection for the X-ray crystal structures of **9a**, **9c**, **9e**, and **10**. Crystals suitable for X-ray diffraction were mounted on a MiTeGen dual-thickness micro-mount and placed under a cold N_2 stream (Oxford). The X-ray radiation employed was generated from a Mo sealed X-ray tube (K_α = 0.70173 Å with a potential of 40 kV and a current of 40 mA). Bruker AXS APEX III software was used for data collection and reduction. Absorption corrections were applied using the program SADABS. Solutions were obtained using XT/XS in APEX III and refined in Olex2-1.5.[5, 6] Hydrogen atoms were placed in idealized positions and were set riding on the respective parent atoms. All non-hydrogen atoms were refined with anisotropic thermal parameters. The structure was refined (weighted least squares refinement on F2) to convergence.[5]

The X-ray crystal structures **9b** and **9d** were collected using synchrotron radiation (either 0.24796 Å and 0.33062 Å) at ChemMatCARS located at the Advanced Photon Source (APS) housed at Argonne National Laboratory (ANL). Crystals suitable for X-ray diffraction were mounted on a glass fiber. Data were collected at 100 K (Cryojet N_2 cold stream) using a vertically mounted Bruker D8 three-circle platform goniometer equipped with a PILATUS3 X CdTe 1M detector. Data was collected as a series of φ and/or ω scans. Data were integrated using SAINT and scaled with a multi-scan absorption correction using SADABS. Structures were solved by intrinsic phasing using SHELXT (Apex3 program suite v2014.1) and refined against F2 on all data by full matrix least squares with SHELXL97. All non-hydrogen atoms were refined anisotropically. H atoms were placed at idealized positions and refined using a riding model.

Solution-Phase Photochemistry. Steady-state photochemical experiments were carried out as follows: A 20-mL vial was charged with compound **1** (1.00 equiv.), the appropriate sulfilimine ligand (2.00 equiv.), the appropriate substrate (>100 equiv.), and CH_2Cl_2 (3.00 mL) inside an N_2-filled glovebox (the vial was capped properly with an electrical tape to keep the mixture under inert atmosphere). The solution was photolyzed by a Nikon Hg 100 W lamp equipped with a glass filter (335 nm) for 24 h at 23 °C. The products were characterized by ^{1}H NMR spectroscopy.

Cryogenic Thin-Film Photochemistry. Preparation of polystyrene thin films containing Rh_2 complexes are detailed on page S50. The prepared sapphire slides were loaded on a cold finger in an optical cryostat (JANIS) under vacuum (1×10^{-5} to 1×10^{-6} mbar). Liquid nitrogen was used as coolant and a temperature controller (Model 335, LakeShore) was used to maintain the reaction temperature during photolysis. A 405 nm CW diode laser (RGB Laser) was used for the photolysis. UV-vis monitoring of the photolysis was measured

with a CCD spectrometer (QE65 Pro, Ocean Optics) with DH UV-vis-NIR light source (200–1100 nm).

B. Synthesis and Characterization

Synthesis of 4,6-Dimethyldibenzo[*b,d*]thiophene (2a)

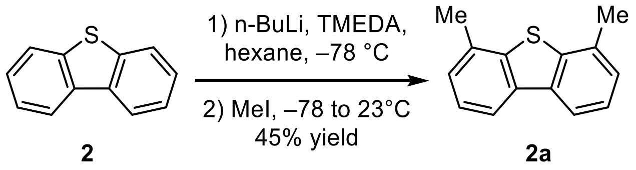

4,6-Dimethyldibenzo[*b,d*]thiophene (**2a**) was prepared according to the following modification of literature methods.[8] Under an N_2 atmosphere, a 100-mL Schlenk flask was charged with TMEDA (2.40 mL, 16.0 mmol, 2.73 equiv.) and dry hexanes (10 mL) and the reaction mixture was cooled to 0 °C. *n*-BuLi (10.0 mL of 2.50 M in hexanes, 25.0 mmol, 4.27 equiv.) was added dropwise over 10 min. The reaction was stirred for 30 min at 0 °C before being warmed to 23 °C and stirred an additional 30 min. The reaction mixture was diluted with 10.0 mL of hexanes and dibenzothiophene (1.08 g, 5.86 mmol, 1.00 equiv.) was added in one portion. The reaction was warmed to 60 °C at which temperature it was maintained for 2 h. The reaction mixture was cooled to −78 °C before methyl iodide (1.00 mL, 16.1 mmol, 2.75 equiv.) was added. The reaction mixture was warmed to 23 °C at which temperature it was stirred for 12 h. The reaction was poured into ice water (50 mL). The reaction was extracted with dichloromethane (3 × 20 mL). The combined organic phase was acidified with 1N HCl (10.0 mL), washed with water (3 × 10 mL), dried over anhydrous Na_2SO_4. Solvent was removed *in vacuo*. The obtained yellow solid was recrystallized from dichloromethane and washed with cold diethyl ether to afford the title compound as white crystalline solid (**2a**, 554 mg, 45% yield) ^1H NMR (δ, 23 °C, CD_2Cl_2): 8.00 (d, *J* = 7.9 Hz, 2H), 7.39 (t, *J* = 7.6 Hz, 2H), 7.28 (dt, *J* = 7.3, 1.0 Hz, 2H), 2.59 (d, *J* = 0.8 Hz, 6H). ^{13}C NMR (δ, 23 °C, CD_2Cl_2): 139.7, 136.37, 132.76, 127.3, 125.2, 119.7, 20.67. Spectral data was previously reported in $CDCl_3$;[9] our data in CD_2Cl_2 is consistent with those reports and spectra are included in Section I.

Synthesis of Dibenzo[*b,d*]thiophene-5-oxide (3a)

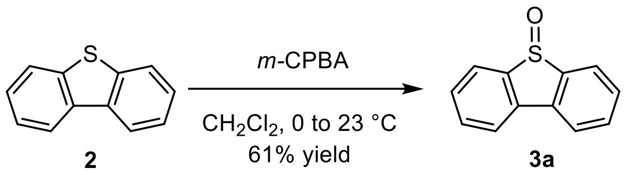

Dibenzo[*b,d*]thiophene-5-oxide (**3a**) was prepared according to the following modification of literature methods.[7] A 100-mL round-bottomed flask was charged with dibenzothiophene (**2**, 3.00 g, 16.3 mmol, 1.00 equiv.) and dichloromethane (15 mL). The reaction solution was cooled to 0 °C. A solution of *meta*-chloroperoxybenzoic acid (77% *m*-CPBA, 3.09 g, 17.9 mmol, 1.10 equiv.) in dichloromethane (15 mL) was slowly added over 10 min at 0 °C. The reaction mixture was allowed to warm to 23 °C at which temperature the reaction was stirred for 12 h. A saturated, aqueous solution of $NaHCO_3$ (100 mL) was

added. The layers were separated, and the aqueous layer was extracted with dichloromethane (3 × 20 mL). The combined organic layers were washed with brine (50 mL) and dried over anhydrous Na_2SO_4. Solvent was removed *in vacuo* and the residue was purified by SiO_2 chromatography with hexanes/ethyl acetate as linear gradient eluent system (v/v: 2:1 to 1:1) to afford the title compound as a white solid (**3a**, 2.00 g, 61% yield). ^{1}H NMR (δ, 23 °C, $CDCl_3$): 8.00 (d, *J* = 7.6 Hz, 2H), 7.82 (d, *J* = 7.7 Hz, 2H), 7.65–7.56 (m, 2H), 7.55–7.47 (m, 2H). Spectral data are consistent with that reported in the literature.[7]

Synthesis of 4,6-dimethyldibenzo[*b,d*]thiophene 5-oxide (3b)

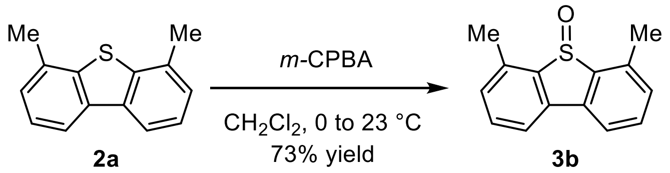

4,6-Dimethyldibenzo[*b,d*]thiophene 5-oxide (**3b**) was prepared according to the following modification of the literature methods.[7] A 25-mL round-bottomed flask was charged with 4,6-dibenzothiophene (**2a**, 213 mg, 1.00 mmol, 1.00 equiv.) and dichloromethane (6 mL). The reaction solution was cooled to 0 °C. A solution of *meta*-chloroperoxybenzoic acid (77% *m*-CPBA, 246.2 mg, 1.43 mmol, 1.10 equiv.) in dichloromethane (6 mL) was slowly added over 5 min at 0 °C. The reaction mixture was allowed to warm to 23 °C at which temperature the reaction was stirred for 2 h. A saturated, aqueous solution of $NaHCO_3$ (10 mL) was added. The layers were separated, and the aqueous layer was extracted with dichloromethane (3 × 5 mL). The combined organic layer was washed with brine (10 mL) and dried over anhydrous Na_2SO_4. Solvent was removed *in vacuo* and the residue was washed with cold hexanes several times to afford the title compound as a white powder (**3b**, 166 mg, 73% yield). ^{1}H NMR (δ, 23 °C, CD_2Cl_2): 7.62 (t, *J* = 9.1 Hz, 2H), 7.47 (t, *J* = 7.6 Hz, 2H), 7.24 (d, *J* = 7.4 Hz, 2H), 2.67 (d, *J* = 15.9 Hz, 6H). ^{13}C NMR (δ, 23 °C, CD_2Cl_2): 143.4, 139.3, 137.8, 133.0, 131.3, 119.9, 18.7. Spectral data was previously reported in $CDCl_3$;[7] our data in CD_2Cl_2 is consistent with those reports and spectra are included in Section I.

Synthesis of 2,4-Dinitrobenzenesulfonamide (4a)

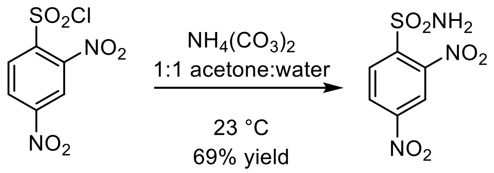

4 4a

2,4-Dinitrobenesulfonamide (**4a**) was prepared according to the following modification of literature methods.[10] A 100-mL round-bottomed flask was charged with 2,4-dinitrobenzenesulfonyl chloride (**4**, 1.10 g, 4.12 mmol, 1.00 equiv.) and $(NH_4)_2CO_3$ (812 mg, 8.45 mmol, 2.05 equiv.) in 1:1 acetone : water mixture (30 mL). The reaction mixture was stirred for 2 h at 23 °C. The acetone was removed *in vacuo* and aqueous layer was extracted with ethyl acetate (3 × 20 mL). The combined organic layer was washed with brine 50.0 mL and dried over anhydrous Na_2SO_4. The solvent was removed *in vacuo*. The residue was washed with hexanes to afford the title compound as a pale-yellow solid. (**4a**, 705 mg, 69% yield). ^{1}H NMR (δ, 23 °C, CD_2Cl_2): 8.71 (d, *J* = 2.2 Hz, 1H), 8.57 (dd, *J* = 8.6, 2.2 Hz, 1H), 8.40 (d, *J* = 8.6 Hz, 1H), 5.65 (s, 2H). ^{13}C NMR (δ, 23 °C, CD_2Cl_2): 141.1, 132.2, 128.2, 125.3. HR-ESI-MS: $[M–H]^-$ = 245.9823 (expt.) and 245.9815 (calc.).

Synthesis of 2,4,6-Triisopropylbenzenesulfonamide (5a)

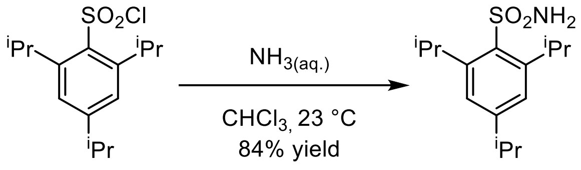

5 5a

2,4-Dinitrobenesulfonamide (**4a**) was prepared according to the following modification of literature methods.[11] A 50-mL round-bottomed flask was charged with 2,4,6-triisopropylbenzenesulfonyl chloride (**5**, 3.10 g, 10.2 mmol, 1.00 equiv.) and 28% aqueous ammonia (6.00 mL, 88.8 mmol, 8.70 equiv.) in chloroform (10 mL). The reaction mixture was stirred at 23 °C for 12 h before the mixture was extracted with chloroform (3 × 20 mL). The combined organic layer was washed with brine (50 mL) and dried over anhydrous $MgSO_4$. The solvent was removed *in vacuo* to obtain the title compound as a white product (**5a**, 2.37 g, 84% yield). ^{1}H NMR (δ, 23 °C, $CDCl_3$): 7.17 (s, 2H), 4.12 (dq, *J* = 13.3, 6.7 Hz, 2H), 2.91 (h, *J* = 6.5 Hz, 1H), 1.3 (dd, *J* = 6.8, 1.5 Hz, 12H), 1.25 (dd, *J* = 6.9, 1.5 Hz, 6H). Spectral data are consistent with that reported in the literature.[11]

General Procedure of Sulfilimine Synthesis (6)

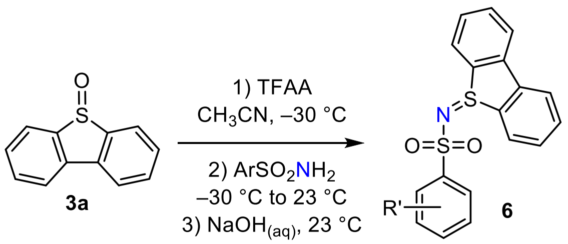

Sulfilimines were prepared according to the following modification of literature methods.[7] A 50-mL round-bottomed flask was charged with dibenzo[*b*,*d*]thiophene-5-oxide (**3a**, 200 mg, 0.998 mmol, 1.00 equiv.) and acetonitrile (6 mL) and the mixture was cooled to –30 °C. Trifluoroacetic anhydride (TFAA, 637 mg, 3.00 mmol, 3.00 equiv.) was added dropwise over 5 min and the reaction mixture was stirred at –30 °C for 20 min. The appropriate sulfonamide (3.00 mmol, 3.00 equiv.) was added, and the reaction mixture was stirred at –30 °C for 8 h. The reaction was warmed to 23 °C before a 0.10 M aqueous solution of sodium hydroxide (10 mL) was added. The layers were separated, and the aqueous layer was extracted with dichloromethane (3 × 20 mL). The combined organic layer was washed with water (50 mL) and dried over anhydrous Na_2SO_4. The solvent was removed *in vacuo*. The residue was purified as specified.

N-(5λ⁴-dibenzo[b,d]thiophen-5-ylidene)-4-methylbenzenesulfonamide (6a)

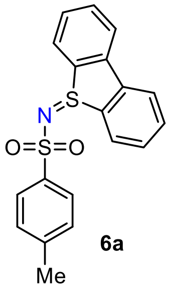

Following the general procedure, the residue was purified by trituration with hot ethyl acetate at 60 °C followed by vacuum filtration to afford sulfilimine **6a** as a white powder (187 mg, 68% yield). ^{1}H NMR (δ, 23 °C, $CDCl_3$): 7.87 (dt, *J* = 7.4, 1.0 Hz, 2H), 7.85–7.80 (m, 2H), 7.69–7.62 (m, 4H), 7.46 (td, *J* = 7.6, 1.1 Hz, 2H), 2.44 (s, 3H). ^{13}C NMR (δ, 23 °C, $CDCl_3$): 142.3, 141.5, 137.7, 137.4, 133.1, 130.2, 129.5, 127.7, 126.8, 122.6, 21.7. IR (KBr pellet): ν = 3082(m), 3057(s), 3024(m), 2925(m), 2893(w), 2852(w), 1597, 1577, 1494, 1481, 1465, 1446(s), 1427(m), 1404(w), 1384(m), 1299(s), 1288(s), 1217, 1145(s), 1085(s), 1020(m), 991(m), 958(s), 939(s), 877(m), 850(m), 815(m), 804(m), 752(s), 707(s), 649(s), 613(w) cm^{-1}. Spectral data are consistent with that reported in the literature.[7]

***N*-(5λ⁴-dibenzo[b,d]thiophen-5-ylidene)-4-nitrobenzenesulfonamide (6b)**

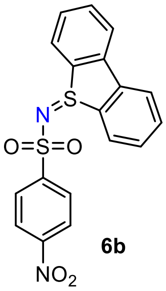

6b

Following the general procedure, the residue was purified by trituration with hot ethyl acetate at 60 °C followed by vacuum filtration to afford sulfilimine **6b** as a white powder (223 mg, 58% yield). ^{1}H NMR (δ, 23 °C, $CDCl_3$): 8.29 (d, *J* = 7.9 Hz, 2H), 8.07 (d, *J* = 8.9 Hz, 2H), 7.95 (d, *J* = 7.43 Hz, 2H), 7.69 (t, *J* = 7.4 Hz, 4H), 7.49 (dt, *J* = 19.0, 7.1 Hz, 2H). ^{13}C NMR (δ, 23 °C, $CDCl_3$): 150.2, 149.5, 137.7, 136.6, 130.5, 128.0, 127.7, 124.2, 122.9. IR (KBr pellet): ν = 3124(w), 3072(w), 1604(w), 1529(s), 1448(m), 1400(w), 1355(s), 1301(s), 1149(s), 1087(s), 951(s), 856(s), 777(m), 756(s), 744(s), 727(s), 703(m), 684(s) cm^{-1}. Spectral data was previously reported in d_6-DMSO;[7] our data in CD_2Cl_2 is consistent with those reports and spectra are included in Section I.

***N*-(5 λ⁴-dibenzo[b,d]thiophen-5-ylidene)-2,4-dinitrobenzenesulfonamide (6c)**

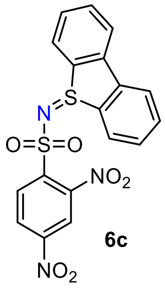

6c

Following the general procedure, the residue was purified by trituration with hot ethyl acetate at 60 °C followed by vacuum filtration to afford sulfilimine **6c** as a white powder (291 mg, 68% yield). ^{1}H NMR (δ, 23 °C, $(CD_3)_2SO$): 8.84 (d, *J* = 2.3 Hz, 1H), 8.52 (dd, *J* = 8.6, 2.3 Hz, 1H), 8.25 (d, *J* = 8.0 Hz, 2H), 8.15 (d, *J* = 8.6 Hz, 1H), 7.81 (t, *J* = 7.8 Hz, 5H), 7.59 (t, *J* = 7.7 Hz, 2H) ^{13}C NMR (δ, 23 °C, $(CD_3)_2SO$): 149.4, 147.1, 140.7, 137.7, 136.6, 133.9, 131.2, 130.7, 127.5, 127.0, 123.7, 120.0. IR (KBr pellet): ν =3095(m), 2904(w), 1604(m), 1558(s), 1539(s), 1481(w), 1465(w), 1449(m), 1433(w), 1352(s), 1319(s), 1300(s), 1151(s), 1132(s), 1107(s), 1051(m), 964(m), 920(m), 875(w), 854(w), 835(w), 752(s), 733(s), 706(w), 661(w), 617(s) cm^{-1}. HR-ESI-MS: $[M–H]^+$ = 430.0156 (expt.) and 430.0162 (calc.).

N-(5λ⁴-dibenzo[b,d]thiophen-5-ylidene)-2,4,6-triisopropylbenzenesulfonamide (6d)

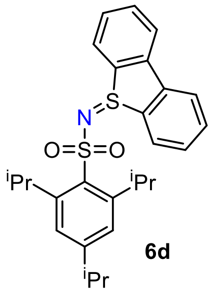

6d

Following the general procedure, the residue was purified by trituration with hot ethyl acetate at 60 °C followed by vacuum filtration to afford sulfilimine **6d** as a white powder (288 mg, 62% yield). ^{1}H NMR (δ, 23 °C, $CDCl_3$): 7.86 (d, *J* = 7.7 Hz, 2H), 7.61 (td, *J* = 7.5, 1.2 Hz, 2H), 7.50–7.43 (m, 2H), 7.39 (td, *J* = 7.6, 1.1 Hz, 2H), 7.14 (s, 2H), 4.43 (h, *J* = 6.7 Hz, 2H), 2.93 (h, *J* = 6.9 Hz, 1H), 1.30 (d, *J* = 6.9 Hz, 6H), 1.19 (d, *J* = 6.8 Hz, 12H). ^{13}C NMR (δ, 23 °C, $CDCl_3$): 151.9, 149.7, 138.6, 138.6, 137.6, 133.2, 130.3, 127.9, 123.8, 122.8, 34.7, 30.0, 25.3, 24.3. IR (KBr pellet): 3070(w), 2968(m), 2871(m), 1597(m), 1560(w), 1462(w), 1442(w), 1423(w), 1381(w), 1361(w), 1301(m), 1288(m), 1251(w), 1193(w), 1159(w), 1138(m), 1105(w), 1057(w), 1039(w), 952(s), 877(m), 866(w), 842(w), 775(w), 752(s), 734(w), 715(s), 686(m), 655(m), 648(m) cm^{-1}. HR-ESI-MS: $[M–H]^+$ = 466.1866 (expt.) and 466.1869 (calc.).

Synthesis of N-(4,6-dimethyl-5λ⁴-dibenzo[b,d]thiophen-5-ylidene)-2,4,6-triisopropylbenzenesulfonamide (6e)

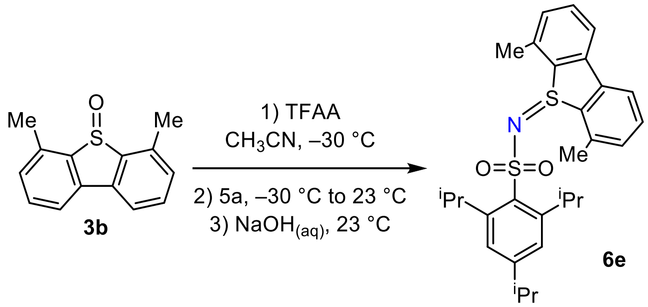

N-(4,6-dimethyl-5λ⁴-dibenzo[b,d]thiophen-5-ylidene)-2,4,6-triisopropylbenzenesulfonamide (**6e**) was prepared according to the following modification of literature methods.[7] A 50-mL round-bottomed flask was charged with 4,6-dimethyldibenzo[*b,d*]thiophene 5-oxide (**3b**, 115 mg, 0.500 mmol, 1.00 equiv.) and acetonitrile (3 mL) and the mixture was cooled to –30 °C. Trifluoroacetic anhydride (TFAA) (317 mg, 1.51 mmol, 3.00 equiv.) was added dropwise over 5 min and the reaction mixture was stirred at –30 °C for 20 min. 2,4,6-Triisopropylbenzenesulfonamide (**5a**, 426 mg 1.50 mmol, 3.00 equiv.) was added, and the reaction mixture was stirred at –30 °C for 8 h. The reaction was warmed to 23 °C before a 0.10 M aqueous solution of sodium hydroxide (5 mL) was added. The layers were

separated, and the aqueous layer was extracted with dichloromethane (3 × 10 mL). The combined organic layer was washed with water (50 mL) and dried over anhydrous Na_2SO_4. The solvent was removed *in vacuo*. The residue was triturated with hot ethyl acetate at 60 °C followed by vacuum filtration to afford the title compound as a white powder (**6e**, 78.4 mg, 32% yield). ^1H NMR (δ, 23 °C, CD_2Cl_2): 7.72 (d, *J* = 7.6 Hz, 2H), 7.53 (t, *J* = 7.6 Hz, 2H), 7.19 (d, *J* = 7.6 Hz, 2H), 7.05 (s, 2H), 4.16 (h, *J* = 6.8 Hz, 2H), 2.86 (h, *J* = 7.0 Hz, 1H), 2.31 (s, 6H), 1.22 (d, *J* = 6.9 Hz, 7H), 1.08 (d, *J* = 6.7 Hz, 12H). ^{13}C NMR (δ, 23 °C, CD_2Cl_2): 151.8, 149.5, 149.0, 139.2, 138.2, 137.0, 133.3, 131.7, 123.4, 120.5, 34.6, 29.8, 24.8, 23.9, 18.9. IR (KBr pellet): 3049 (w), 2953(m), 2927(m), 2868(m), 1598(m), 1562(w), 1460(m), 1423(m), 1382(m), 1365(m), 1301(m), 1290(m), 1251(w), 1193(w), 1163(w), 1139(s), 1105(w), 1060(w), 1040(m), 959(s), 880(w), 783(s), 709(s), 661(s), 619(w) cm^{-1}. HR-ESI-MS: $[M–H]^+$ = 494.2182 (expt.) and 494.2168 (calc.).

General Procedure of Arylsulfonyl Azide Synthesis (8)

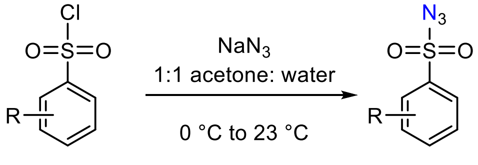

7 **8**

SAFETY Organic azides are potentially explosive compounds. Proper safety precautions must be taken during storage and usage.

Arylsulfonyl azides were prepared according to the following modification of literature methods.[12] A 50-mL round-bottomed flask was charged with the appropriate aryl sulfonyl chloride, (**7**, 1.00 mmol, 1.00 equiv.) and acetone (5 mL) and the reaction mixture was cooled to 0 °C. Sodium azide (130 mg, 2.00 mmol, 2.00 equiv.) was dissolved in water (5 mL) and was added to the reaction mixture dropwise. The reaction mixture was slowly warmed up to 23 °C at which temperature it was stirred for 12 h. The acetone was removed *in vacuo* and the aqueous layer was extracted with ethyl acetate (3 × 10 mL). The combined organic layer was washed with 5% Na_2CO_3 (10 mL) and brine (20 mL) and dried over anhydrous Na_2SO_4. The solvent was removed *in vacuo*.

4-Methylbenzenesulfonyl Azide (8a)

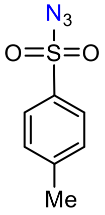

8a

Following the general procedure, the title compound was obtained as colorless oil which turned into white solid upon storage at −20 °C (**8a**, 170 mg, 86% yield). ^{1}H NMR (δ, 23 °C, $CDCl_3$): 7.91–7.77 (m, 2H), 7.41 (d, *J* = 8.2 Hz, 2H), 2.48 (s, 3H). ^{13}C NMR (δ, 23 °C, $CDCl_3$): 146.3, 135.7, 130.42 127.7, 21.91. IR (ATR, cm^{-1}) 2357(w), 2121(s), 1595(m), 1495(w), 1450 (w), 1366 (s), 1307 (w), 1297 (w), 1162 (s), 1120 (m), 1084 (s), 1018 (w), 813 (m), 743 (s), 702 (m), 657 (s). Spectral data are consistent with that reported in the literature.[13]

4-Nitrobenzenesulfonyl Azide (8b)

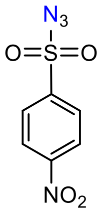

8b

Following the general procedure, the title compound was obtained as beige white solid (**8b**, 189.0 mg, 83% yield). ^{1}H NMR (δ, 23 °C, $CDCl_3$): 8.50–8.43 (m, 2H), 8.20–8.14 (m, 2H).^{13}C NMR (δ, 23 °C, $CDCl_3$): 151.4, 143.9, 129.0, 125.1. IR (ATR, cm^{-1}) 3107(m), 2316(m), 2141(s), 1528(s), 1375(s), 1349(s) 1309(s), 1175(s), 1156(s), 1085(s), 854(s), 603(m), 582(m). Spectral data are consistent with that reported in the literature.[14]

2,4,6-triisopropylbenzenesulfonyl azide (8c)

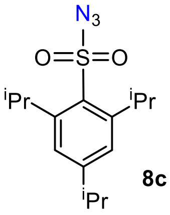

8c

Following the general procedure, the title compound was obtained as white solid (**8c**, 181.6 mg, 58% yield). ^{1}H NMR (δ, 23 °C, $CDCl_3$): 7.22 (s, 2H), 4.05 (h, *J* = 6.7 Hz, 2H), 2.94 (dq, *J* = 13.8, 6.9 Hz, 1H), 1.28 (dd, *J* = 7.8, 6.8 Hz, 18H). ^{13}C NMR (δ, 23 °C, $CDCl_3$): 155.0, 151.1, 132.2, 124.3, 34.5, 30.0, 24.90, 23.6. IR (ATR, cm^{-1}) 2962(m), 2929(w), 2870(w), 2113(s),

1569(m), 1427(m), 1379(m), 1352(m), 1256, 1197(s), 1164(s), 1105(m), 1031(m), 885(m), 738(s), 653(s). Spectral data are consistent with that reported in the literature.[15]

Synthesis of $Rh_2esp_2(6a)_2$ (9a)

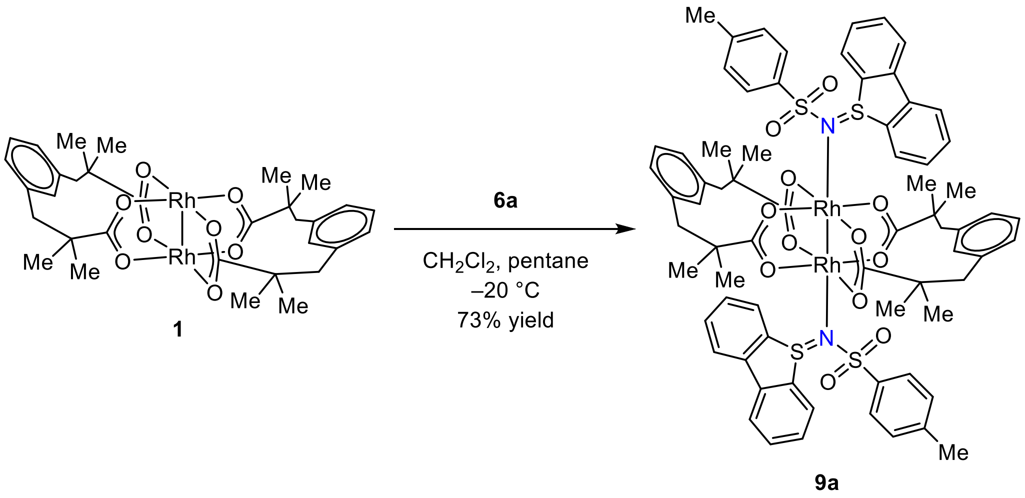

A 20-mL vial was charged with complex **1** (10.4 mg, 0.0137 mmol, 1.00 equiv.), compound **6a** (10.2 mg, 0.0289 mmol, 2.11 equiv.), and CH_2Cl_2 (3 mL) inside a N_2-filled glovebox and the reaction mixture was stirred at 23° C for 1 h. The reaction mixture was cooled to −20 °C, at which temperature the mixture was maintained for 2 d to afford dark-green crystals. The supernatant was decanted, and the crystals were washed with pentane and dried *in vacuo* at 23 °C to afford the title compound (**9a**, 16.8 mg, 84% yield). ^1H NMR (δ, 23 °C, $CDCl_3$): 7.89 (d, *J* = 7.8 Hz, 4H), 7.80 (d, *J* = 8.1 Hz, 4H), 7.69–7.60 (m, 8H), 7.43 (t, *J* = 7.6 Hz, 4H), 7.20 (d, *J* = 7.8 Hz, 4H), 7.06 (t, *J* = 7.5 Hz, 2H) 7.0(s, 2H), 6.83 (dd, *J* = 7.5, 1.8 Hz, 4H), 2.63 (s, 8H), 2.42 (s, 6H), 1.02 (s, 24H) ^{13}C NMR (δ, 23 °C, $CDCl_3$): 196.3, 141.8, 138.3, 137.7, 132.9, 130.0, 129.2, 128.0, 127.7, 122.6, 53.6, 47.2, 46.4, 21.7. IR (KBr pellet): 3055(w), 2966(m), 2949(m), 2920(m), 2886(w), 1583(s), 1475(s), 1448(m), 1407(s), 1385(m), 1380(m), 1357(w), 1315(m), 1311(m), 1263(m), 1244(m), 1199(w) 1151(s), 1087(m), 1059(w), 1020(w), 929(s), 881(w), 820(w), 798(w), 759(m), 738(s), 711(m), 657(m),630(m) cm^{-1}. UV-vis (CH_2Cl_2), λ_{max} (nm, ε ($M^{-1}cm^{-1}$)): 422 (180), 639 (214). HR-ESI-MS: $[M–H]^+$ = 1465.2006 (expt.) and 1465.1994 (calc.). $[M–Na]^+$ = 1487.1798 (expt.) and 1487.1814 (calc.).

Synthesis of $Rh_2esp_2(6b)_2$ (9b)

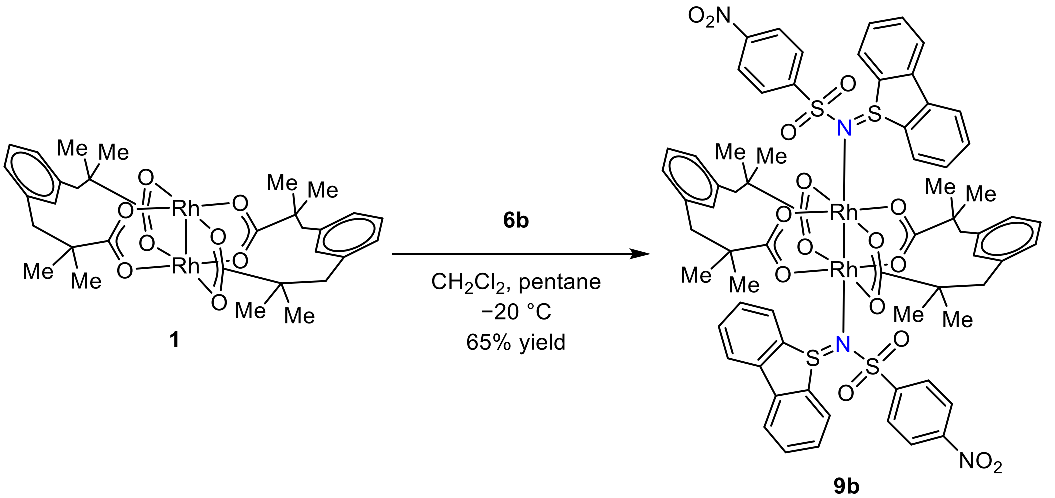

A 20-mL vial was charged with complex **1** (15.3 mg, 0.0202 mmol, 1.00 equiv.), compound **6b** (16.4 mg, 0.0427 mmol, 2.11 equiv.), and CH_2Cl_2 (3 mL) inside a N_2-filled glovebox and the reaction mixture was stirred at 23° C for 1 h. The reaction mixture was cooled to −20 °C, at which temperature the mixture was maintained for 2 d to afford dark-green crystals. The supernatant was decanted, and the crystals were washed with pentane and dried *in vacuo* at 23 °C to afford the title compound (**9b**, 19.1 mg, 65% yield). ^{1}H NMR (δ, 23 °C, $CDCl_3$): 8.2 (d, J = 8.3 Hz, 4H), 8.04 (d, J = 8.7 Hz, 4H), 7.92 (d, J = 7.7 Hz, 4H), 7.69 (td, J = 7.6, 1.1 Hz, 4H), 7.64 (d, J = 7.8 Hz, 4H), 7.46 (t, J = 7.6 Hz, 4H), 7.10 (t, J = 7.5 Hz, 2H), 6.97 (s, 2H), 6.85 (dd, J = 7.5, 1.8 Hz, 4H), 2.64 (s, 8H), 1.01 (s, 24H). ^{13}C NMR (δ, 23 °C, $CDCl_3$):196.3, 150.6, 149.4, 138.4, 138.0, 136.5, 133.4, 131.5, 130.2, 128.3, 128.0, 127.6, 126.8, 123.8, 122.8, 53.6, 47.2, 46.2, 29.8, 25.9. IR (KBr pellet): 3103(w), 3078(w), 3033(w), 2982(m), 2972(m), 2949(m), 2924(m), 2870(m), 1606(w), 1582(s), 1531(s), 1475(m), 1448(m), 1408(s), 1385(m), 1375(m), 1350(s), 1335(m), 1317(m), 1263(m), 1246(m), 1200(w), 1159(s), 1134(m), 1088(m), 945(m), 912(s), 856(m), 825(w), 752(s), 746(s), 721(m), 711(m), 687(m), 632(m), 617(m) cm^{-1}. UV-vis (CH_2Cl_2), λ_{max} (nm, ε ($M^{-1}cm^{-1}$)): 429 (395), 641 (277). HR-ESI-MS: $[M–H]^+$ = 1465.2006 (expt.) and 1465.1994 (calc.).

Synthesis of $Rh_2esp_2(6c)_2$ (9c)

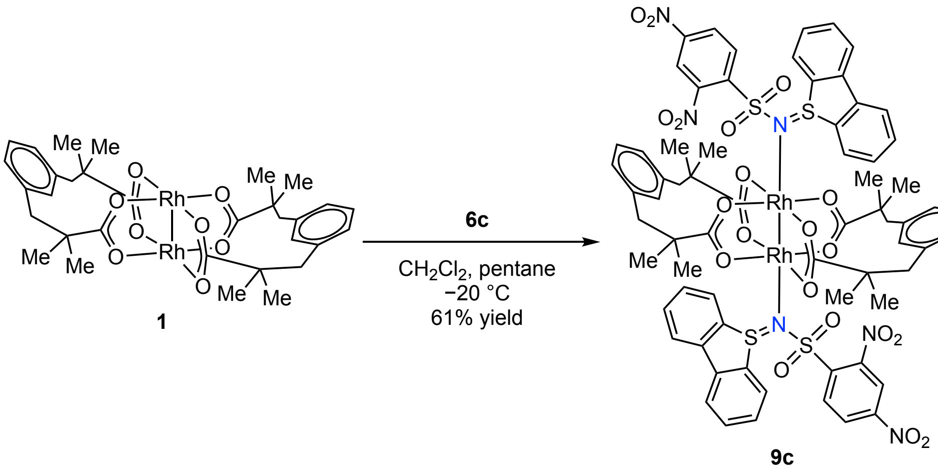

A 20-mL vial was charged with complex **1** (9.60 mg, 0.0123 mmol, 1.00 equiv.), compound **6c** (11.5 mg, 0.0268 mmol, 2.17 equiv.), and CH_2Cl_2 (3 mL) inside a N_2-filled glovebox and the reaction mixture was stirred at 23° C for 1 h. The reaction mixture was cooled to −20 °C, at which temperature the mixture was maintained for 2 d to afford dark-green crystals. The supernatant was decanted, and the crystals were washed with pentane and dried in vacuo at 23 °C to afford the title compound (**9c**, 12.5 mg, 61% yield). ^1H NMR (δ, 23 °C, CD_2Cl_2): 8.44–8.30 (m, 4H), 8.26 (dd, J = 8.6, 2.2 Hz, 2H), 8.00–7.92 (m, 4H), 7.78–7.63 (m, 8H), 7.46 (td, J = 7.7, 1.1 Hz, 4H), 7.12 (t, J = 7.5 Hz, 2H), 6.98 (t, J = 1.9 Hz, 2H), 6.85 (dd, J = 7.5, 1.8 Hz, 4H), 2.61 (s, 8H), 0.98 (s, 24H) ^{13}C NMR (δ, 23 °C, CD_2Cl_2): 197.0, 139.4, 148.0, 143.2, 138.6, 138.5, 136.6, 134.0, 132.9, 131.6, 130.7, 128.3, 127.8, 127.1, 126.5, 123.4, 119.5, 47.3, 46.6, 25.9. IR (KBr pellet): 3097(m), 2964(m), 2924(m), 2870(m), 1579(s), 1553(s), 1539(s), 1475(s), 1148(s), 1409(s), 1350(s), 1319(m), 1265(m), 1244(m), 1161(s), 1149(s), 1105(s), 1051(m), 974(s), 920(s), 902(s), 833(m), 752(s), 733(s), 709(s), 659(m), 632(m), 617(m) cm^{-1}. UV-vis (CH_2Cl_2), λ_{max} (nm, ε ($M^{-1}cm^{-1}$)): 430 (259), 655 (218). HR-ESI-MS: $[M–NH_4]^+$ = 1634.1361(expt.) and 1634.1350(calc.).

Synthesis of $Rh_2esp_2(6d)_2$ (9d)

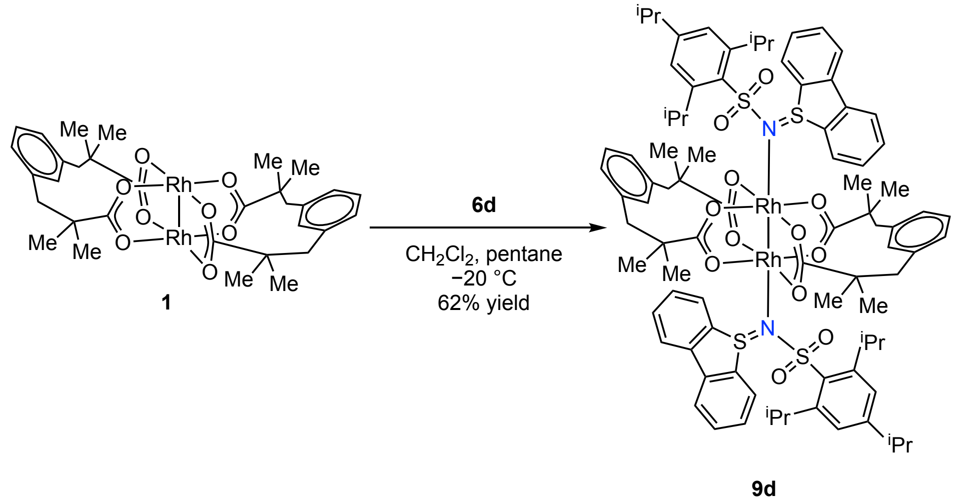

A 20-mL vial was charged with complex **1** (16.0 mg, 0.0211 mmol, 1.00 equiv.), compound **6d** (19.1 mg, 0.0410 mmol, 1.94 equiv.), and CH_2Cl_2 (3 mL) inside an N_2-filled glovebox and the reaction mixture was stirred at 23° C for 1 h. The reaction mixture was cooled to −20 °C, at which temperature the mixture was maintained for 2 d to afford pale-green crystals. The supernatant was decanted, and the crystals were washed with pentane and dried in vacuo at 23 °C to afford the title compound (**9d**, 22.1 mg, 62% yield). ^1H NMR (δ, 23 °C, $CDCl_3$): 7.86 (d, *J* = 7.7 Hz, 4H), 7.62 (td, *J* = 7.6, 1.2 Hz, 4H), 7.48 (d, *J* = 7.7 Hz, 4H), 7.40 (td, *J* = 7.7, 1.1 Hz, 4H), 7.14 (s, 4H), 7.05 (t, *J* = 7.5 Hz, 2H), 6.83 (td, *J* = 7.6, 1.7 Hz, 6H), 4.43 (h, *J* = 6.8 Hz, 4H), 2.93 (h, *J* = 7.0 Hz, 2H), 2.62 (s, 8H), 1.30 (d, *J* = 6.9 Hz, 12H), 1.19 (d, *J* = 6.7 Hz, 24H), 0.99 (s, 24H). ^{13}C NMR (δ, 23 °C, $CDCl_3$): 196.8, 151.7, 149.7,138.1, 137.4, 132.9, 129.9, 128.1,127.8, 123.5, 122.5, 47.0, 46.5, 34.4, 34.2, 29.8, 25.8, 25.1, 24.1, 22.5, 14.2. IR (KBr pellet): 3084(w), 3057(w), 2954(m), 2925(w), 2869(w), 2852(w), 2829(w) 1637(m), 1585(s), 1542(w), 1475(m), 1448(w), 1473(w), 1460(w),1448(w), 1407(s) 1385(s), 1321(m), 1305(m), 1263(m), 1203(w), 1151(m), 1105(w), 1072, 1058(w), 1039(w), 947(w), 923(w), 896(m), 842(w), 822(w), 752(s), 717(s), 686(w), 632(w), 615(w). UV-vis (CH_2Cl_2), λ_{max} (nm, ε ($M^{-1}cm^{-1}$)): 422 (167), 662 (200). HR-ESI-MS: $[M–H]^+$ = 1689.4471 (expt.) and 1689.4498 (calc.).

Synthesis of $Rh_2esp_2(6e)_2$ (9e)

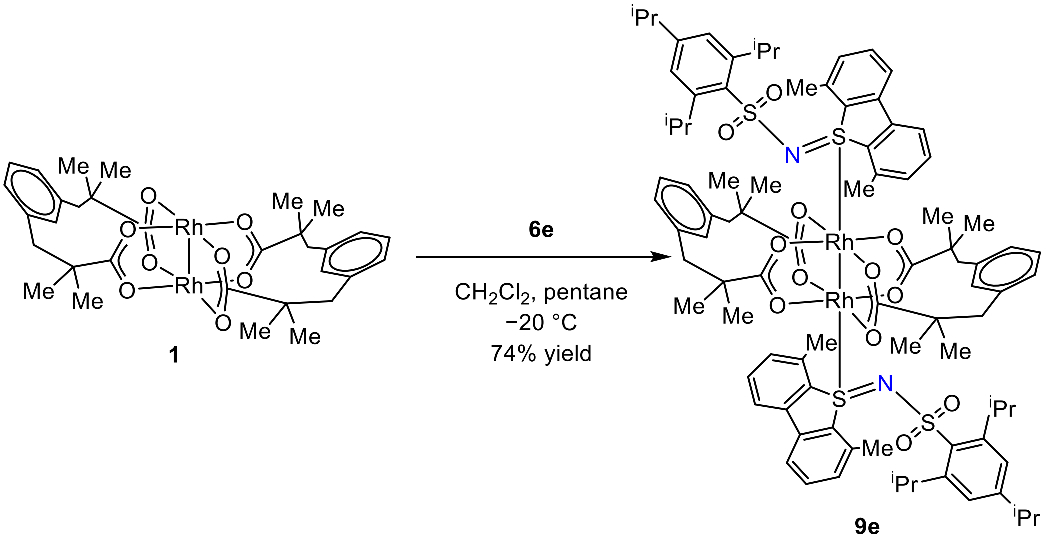

A 20-mL vial was charged with complex **1** (11.7 mg, 0.0154 mmol, 1.00 equiv.), compound **6e** (15.5 mg, 0.0314 mmol, 2.04 equiv.), and CH_2Cl_2 (3 mL) inside a N_2-filled glovebox and the reaction mixture was stirred at 23° C for 1 h. The reaction mixture was cooled to −20 °C, at which temperature the mixture was maintained for 2 d to afford dark-green crystals. The supernatant was decanted, and the crystals were washed with pentane and dried in vacuo at 23 °C to afford the title compound (**9e**, 18.9 mg, 74% yield). ^1H NMR (δ, 23 °C, $CDCl_3$): 7.67 (d, *J* = 7.6 Hz, 4H), 7.50 (t, *J* = 7.6 Hz, 4H), 7.18 (d, *J* = 7.5 Hz, 4H), 7.06 (s, 6H), 6.89 (s, 2H), 6.83 (d, *J* = 7.6 Hz, 4H), 4.31 (p, *J* = 6.7 Hz, 4H), 2.87 (h, *J* = 6.9 Hz, 2H), 2.61 (s, 8H), 2.35 (s, 12H), 1.13 (d, *J* = 6.7 Hz, 24H), 1.25 (d, *J* = 6.9 Hz, 12H), 0.99 (s, 24H). ^{13}C NMR (δ, 23 °C, $CDCl_3$):196.7, 151.3, 149.0, 139.3, 138.2, 138.0, 136.7, 133.0, 123.1, 120.1, 47.06, 34.3, 29.7, 25.8, 25.0, 24.0, 18.9. IR (KBr pellet): 3080(w), 3057(w), 2981(m), 2960(m), 2947(m), 2922(m), 2868(w), 1691(s), 1584(s), 1474(s), 1446(m), 1406(s), 1385(m), 1373(s), 1358(s), 1263(m), 1240(s), 1201(w), 1164(w), 1132(m), 1084(w), 962(w), 925(w), 900(w), 881(w), 825(w), 779(w), 750(w), 709(m), 690(w), 655(w), 632(m) cm^{-1}. UV-vis (CH_2Cl_2), λ_{max} (nm, ε ($M^{-1}cm^{-1}$)): 415 (288), 658 (224). $[M–H]^+$ = 1745.5129 (expt.) and 1745.5124 (calc.).

Synthesis of $Rh_2esp_2(dbt)_2$ (10)

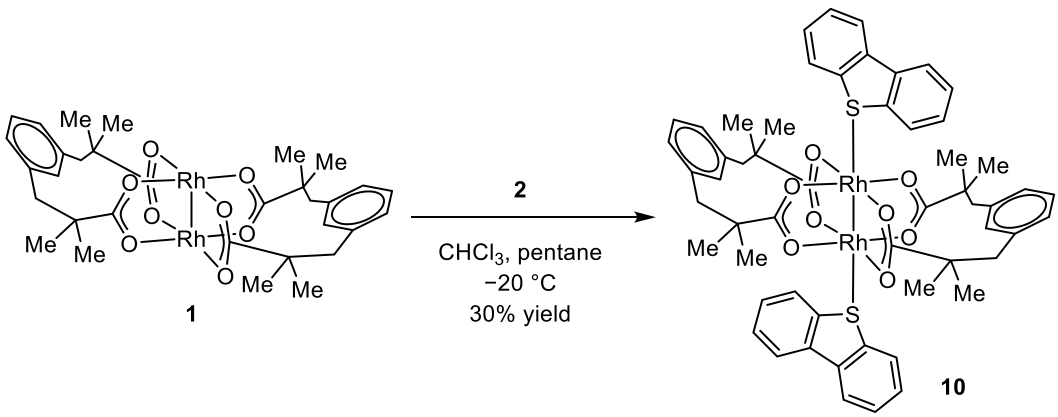

A 20-mL vial was charged with complex **1** (21.7 mg, 0.0286 mmol, 1.00 equiv.), compound **2** (11.3 mg, 0.0613 mmol, 2.14 equiv.) and dry CH_3Cl (3 mL) and the reaction mixture was stirred at 23° C for 2 h. The reaction mixture was cooled to −20 °C, at which temperature the mixture was maintained for 2 d to afford dark-green crystals. The supernatant was decanted, and the crystals were washed with pentane and dried in vacuo at 23 °C to afford the title compound (**10**, 9.67 mg, 30% yield). ^1H NMR (δ, 23 °C, $CDCl_3$): 8.22–8.11 (m, 4H), 7.94 (dd, *J* = 6.4, 2.3 Hz, 4H), 7.53–7.43 (m, 8H), 7.03 (d, *J* = 15.1 Hz, 2H), 6.79 (dd, *J* = 7.5, 1.8 Hz, 4H), 6.57 (s, 2H), 2.50 (s, 8H), 0.92 (s, 24H). ^{13}C NMR (δ, 23 °C, $CDCl_3$): 196.2, 138.7, 138.0, 136.7, 131.0, 127.9, 127.0, 126.7, 125.4, 124.6, 121.7, 47.0, 46.3, 25.7. IR (KBr pellet): 3057(w), 2964(m), 2920(m), 2868(w), 1633(w), 1581(s), 1473(m), 1444(w), 1407(s), 1384(s), 1357(w), 1303(w), 1290(w), 1263(w), 1134(m), 976(m), 823(w), 744(s), 709(s), 634(m), 568(w). UV-vis (CH_2Cl_2), λ_{max} (nm, ε ($M^{-1}cm^{-1}$)): 434 (316), 635 (228). $[M–H]^+$ = 1127.5 (expt.) and 1127.2 (calc.)

C. Supporting Data

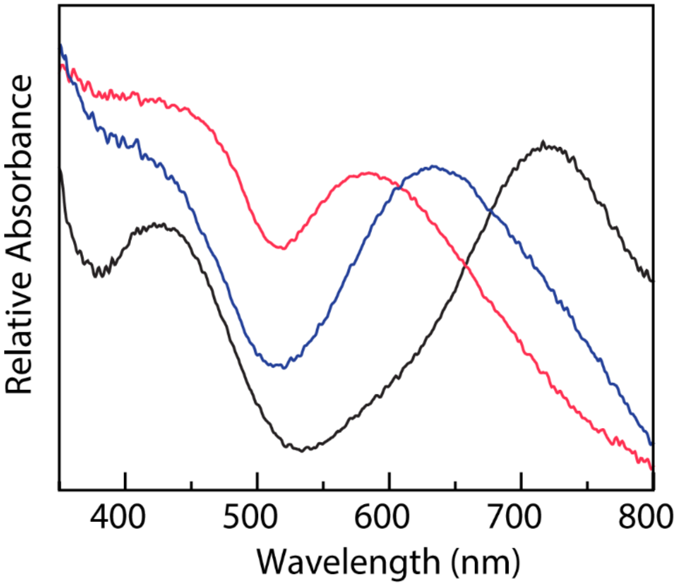

Figure S1. Solid-state UV-vis spectra were obtained for crystalline samples of compounds **1**, **9b**, and **9d** in diffuse reflectance mode. In the solid state, the low energy bands of **9b** (—) and **9d** (—) are blue shifted to 582 and 632 nm with respect to compound **1** (—) where low energy band is centered at 720 nm.

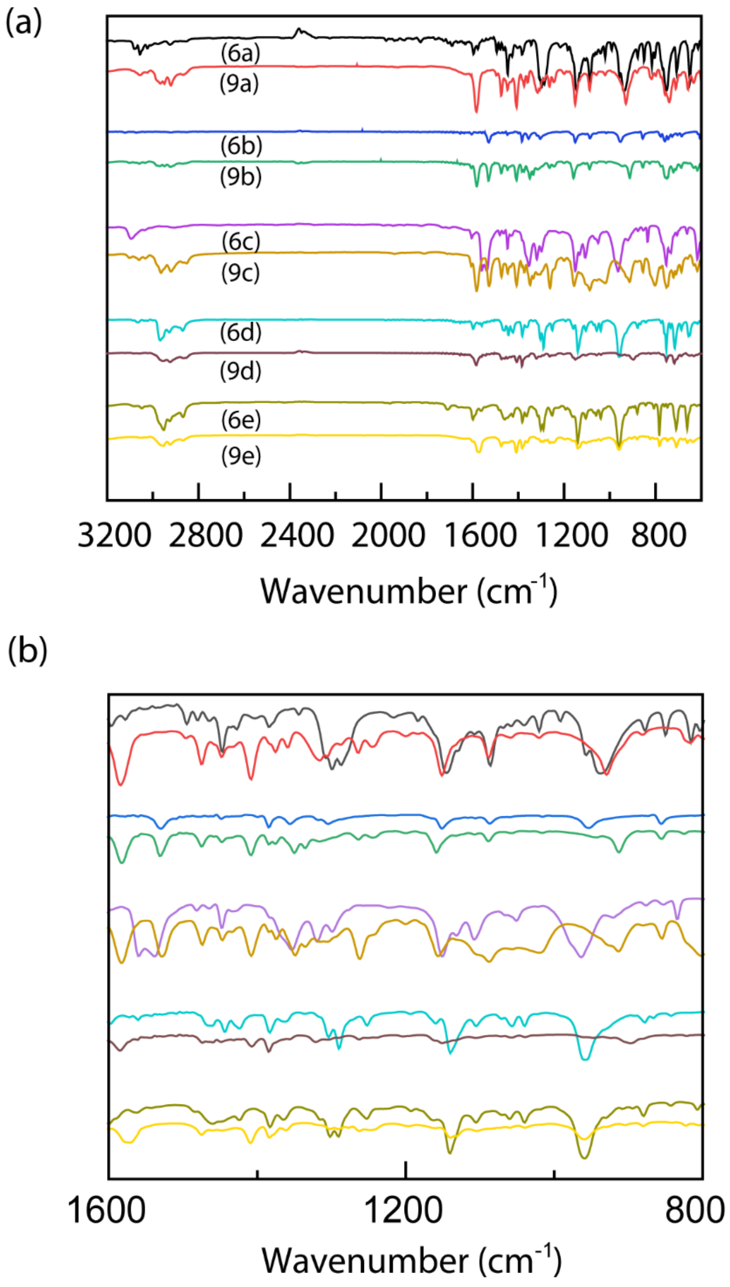

Figure S2. (**a**) Comparison of the IR spectra of the sulfilimines (**6a-6e**) with their corresponding Rh_2 complexes (**9a-9e**) in the spectral window of 3200 to 600 cm^{-1}. (**b**) Comparison of the IR spectra of the sulfilimines (**6a-6e**) with their corresponding Rh_2 complexes (**9a-9d**) in the spectral window of 1600 to 800 cm^{-1}. For **6a** (—)$\nu_{N=S}$ = 939 cm^{-1} while for **9a** (—) $\nu_{N=S}$ = 929 cm^{-1}; For **6b** (—) $\nu_{N=S}$ = 954 cm^{-1} while for **9b** (—) $\nu_{N=S}$ = 912 cm^{-1}; For **6c** (—) $\nu_{N=S}$ = 964 cm^{-1} while for **9c** (—) $\nu_{N=S}$ = 914 cm^{-1}; For **6d** (—) $\nu_{N=S}$ = 960 cm^{-1} while for **9d** (—) $\nu_{N=S}$ = 896 cm^{-1}; For **6e** (—) $\nu_{N=S}$ = 958 cm^{-1} while for **9e** (—) $\nu_{N=S}$ = 960 cm^{-1}.

D. Ligand Binding Titrations

Titration of $Rh_2(esp)_2$ with 6a

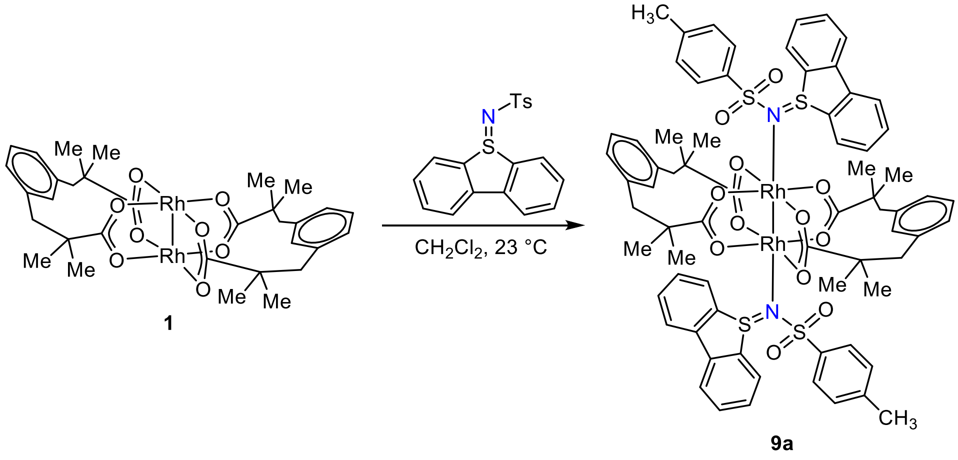

A 20-mL vial was charged with compound **1** (15.4 mg, 0.0203 mmol, 1.00 equiv.) and dissolved in CH_2Cl_2 (8.00 mL). Another 20-mL vial was charged with **6a** (15.2 mg, 0.0430 mmol, 2.11 equiv.) and dissolved in CH_2Cl_2 (1.60 mL). A cuvette was charged with the stock solution of **1** (2.10 mL) and it was titrated with the stock solution of (0–0.00156 M) and monitored *via* UV-vis until the spectrum stopped evolving (Figure S3).

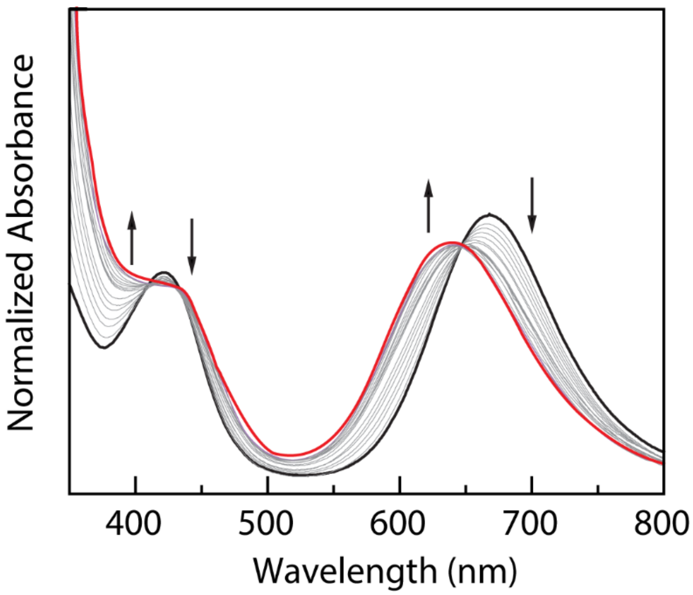

Figure S3. UV-vis spectra obtained during titration of **1** with **6a**. Spectra were collected in CH_2Cl_2 at 23 °C. The initial concentration of **1** was 0.00254 M. UV-vis spectra collected at [**6a**] of 0–0.00516 M. The well-anchored isosbestic points at 434 and 639 nm indicate the absence of steady-state intermediates in the conversion of **1** (—) to **9a** (—).

Titration of $Rh_2(esp)_2$ with 6b

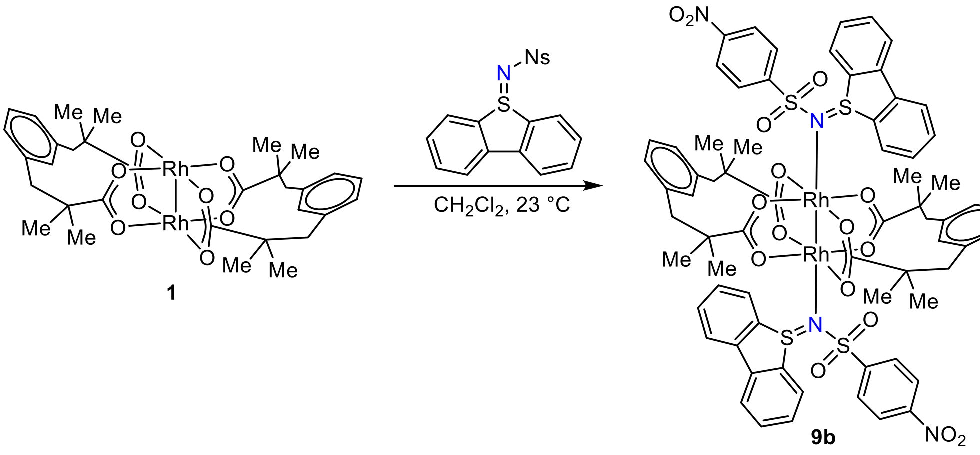

A 20-mL vial was charged with compound **1** (16.2 mg, 0.0214 mmol, 1.00 equiv.) and dissolved in CH_2Cl_2 (8.00 mL). Another 20-mL vial was charged with **6b** (12.1 mg, 0.401 mmol, 1.47 equiv.) and dissolved in CH_2Cl_2 (1.50 mL). A cuvette was charged with the stock solution of **1** (2.30 mL) and it was titrated with the stock solution of (0–0.00489 M) and monitored *via* UV-vis until the spectrum stopped evolving (Figure S4).

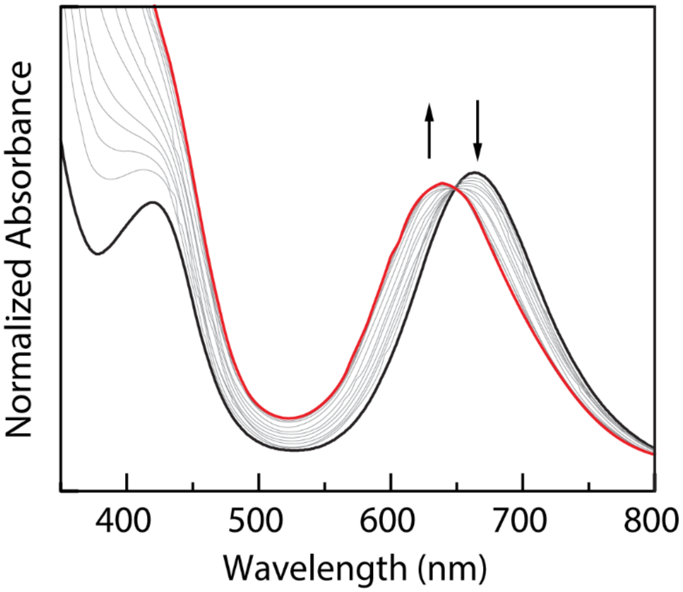

Figure S4. UV-vis spectra obtained during titration of **1** with **6b.** Spectra were collected in CH_2Cl_2 at 23 °C. The initial concentration of **1** was 0.00267 M. (a) UV-vis spectra collected at [**6b**] of 0–0.00489 M. The well-anchored isosbestic point at 647 nm indicates the absence of steady-state intermediates in the conversion of **1** (—) to **9b** (—).

Titration of $Rh_2(esp)_2$ with 6c

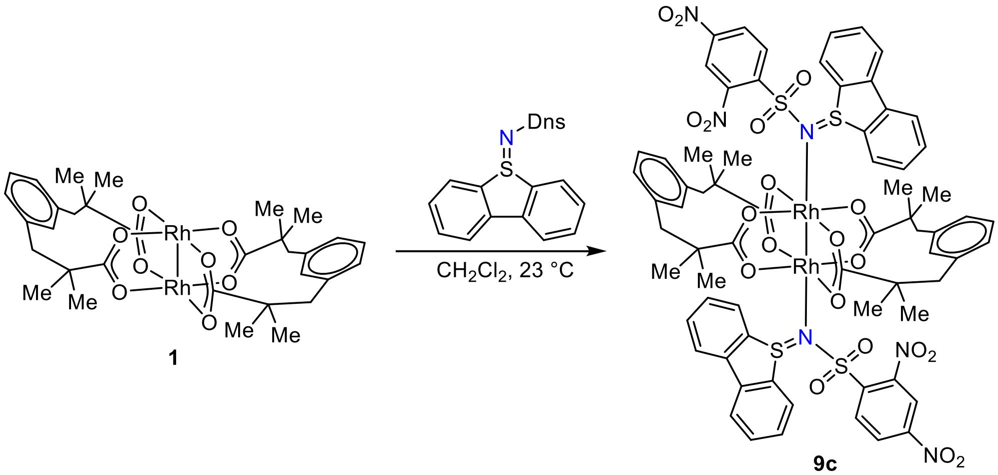

A 20-mL vial was charged with compound **1** (17.8 mg, 0.0235 mmol, 1.00 equiv.) and dissolved in CH_2Cl_2 (9.00 mL). Another 20-mL vial was charged with **6c** (15.5 mg, 0.0361 mmol, 1.54 equiv.) and dissolved in CH_2Cl_2 (2.50 mL). A cuvette was charged with the stock solution of **1** (2.30 mL) and it was titrated with the stock solution of (0–0.00495 M) and monitored *via* UV-vis until the spectrum stopped evolving (Figure S5).

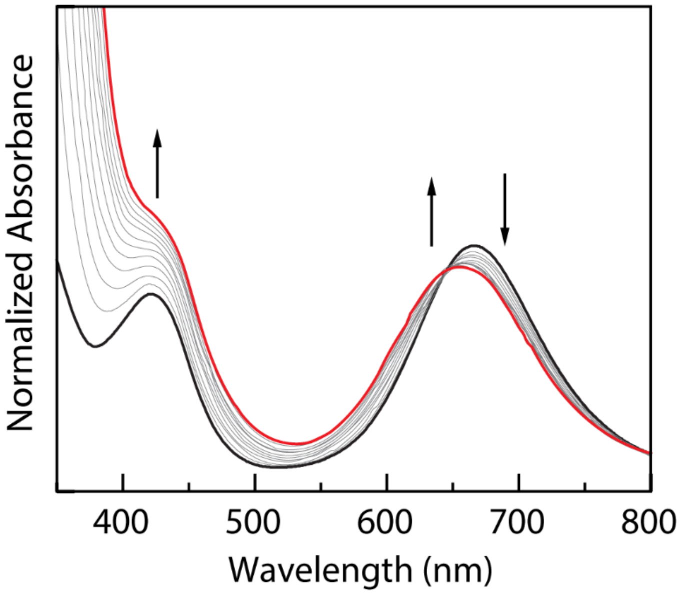

Figure S5. UV-vis spectra obtained during titration of **1** with **6c**. Spectra were collected in CH_2Cl_2 at 23 °C. The initial concentration of **1** was 0.00261 M. (a) UV-vis spectra collected at [**6c**] of 0–0.00495. The well-anchored isosbestic point at 644 nm indicates the absence of steady-state intermediates in the conversion of **1** (—) to **9c** (—).

Titration of $Rh_2(esp)_2$ with 6d

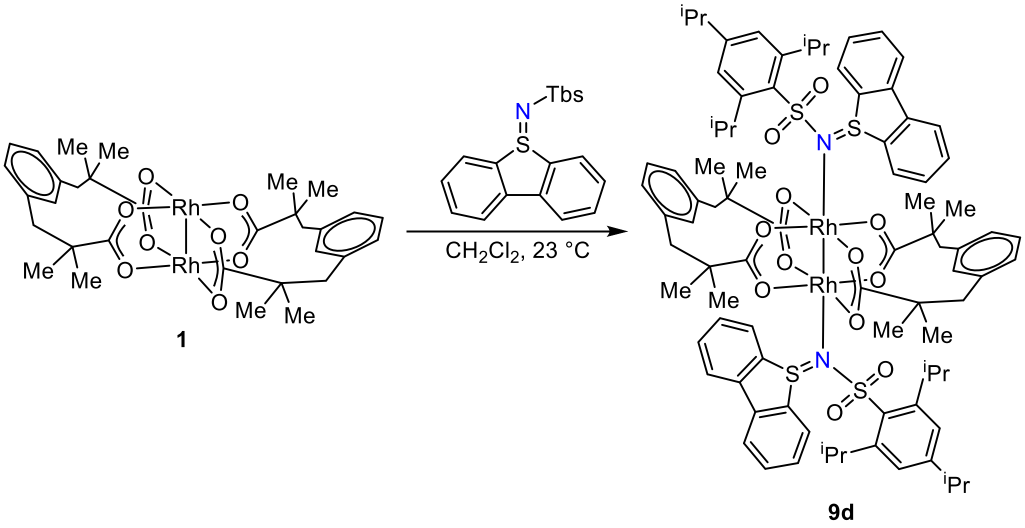

A 20-mL vial was charged with compound **1** (17.8 mg, 0.0235 mmol, 1.00 equiv.) and dissolved in CH_2Cl_2 (9.00 mL). Another 20-mL vial was charged with **6d** (25.3 mg, 0.0544 mmol, 2.31 equiv.) and dissolved in CH_2Cl_2 (1.00 mL). A cuvette was charged with the stock solution of **1** (2.30 mL) and it was titrated with the stock solution of (0–0.00539 M) and monitored *via* UV-vis until the spectrum stopped evolving (Figure S6).

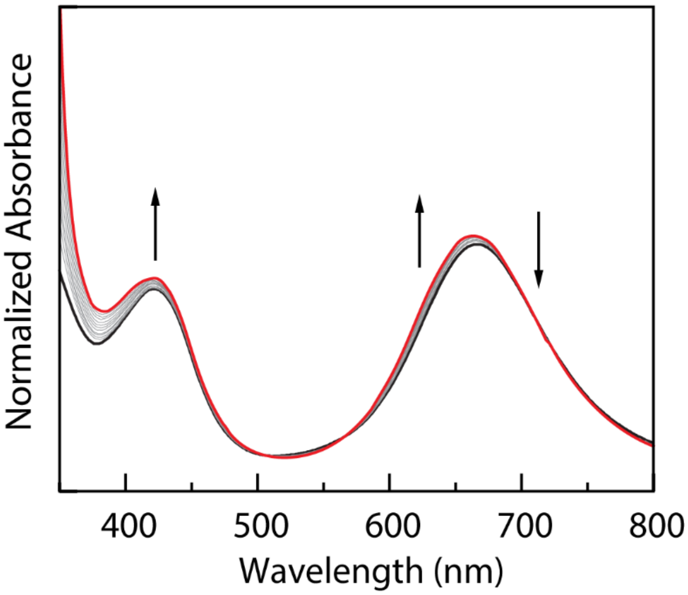

Figure S6. UV-vis spectra obtained during titration of **1** with **9d**. Spectra were collected in CH_2Cl_2 at 23 °C. The initial concentration of **1** was 0.00261 M. (a) UV-vis spectra collected at [**6d**] of 0–0.00539 M. The well-anchored isosbestic points at 567 and 505 nm indicate the absence of steady-state intermediates in the conversion of **1** (—) to **9d** (—).

Titration of $Rh_2(esp)_2$ with 8a

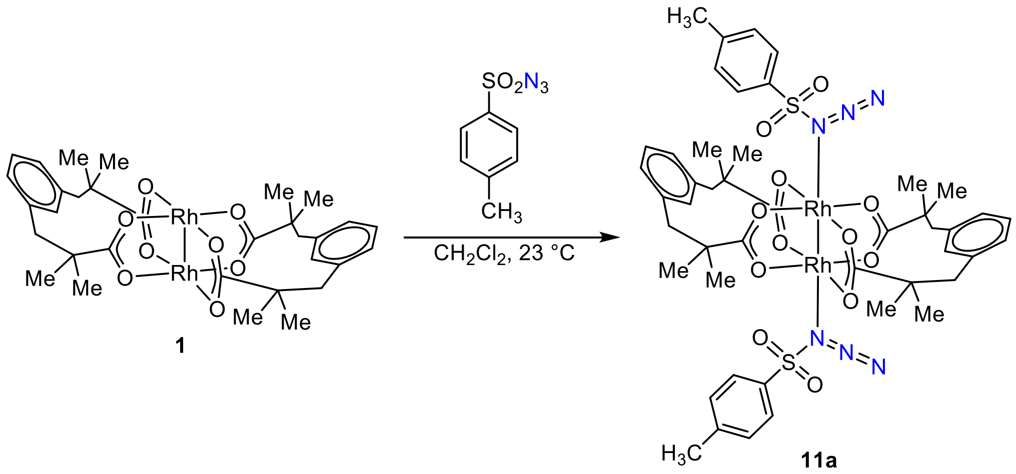

A 20-mL vial was charged with compound **1** (16.2 mg, 0.0214 mmol, 1.00 equiv.) and dissolved in CH_2Cl_2 (8.00 mL). Another 20-mL vial was charged with **8a** (52.0 mg, 0.264 mmol, 12.3 equiv.) and dissolved in CH_2Cl_2 (1.00 mL). A cuvette was charged with the stock solution of **1** (2.30 mL) and it was titrated with the stock solution of (0–0.0648 M) and monitored *via* UV-is until the spectrum stopped evolving (Figure S7).

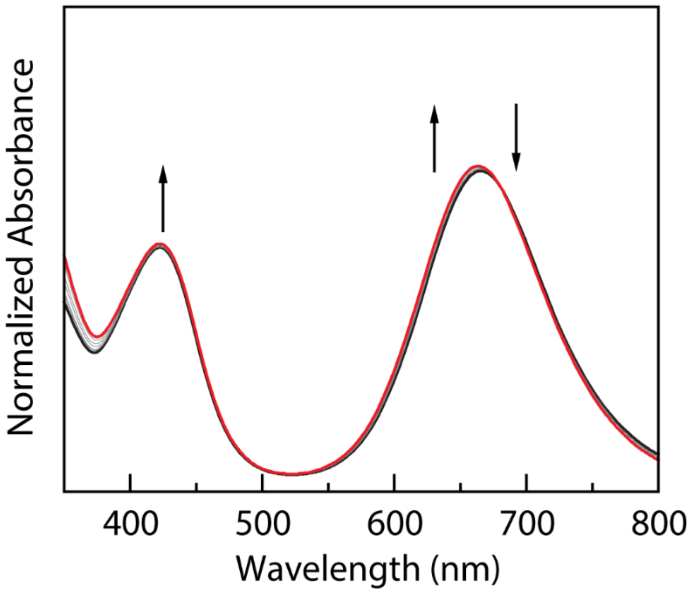

Figure S7. UV-vis spectra obtained during titration of with **8a**. Spectra were collected in CH_2Cl_2 at 23 °C. The initial concentration of complex **1** was 0.00267 M. (a) UV-vis spectra collected at [**8a**] of 0–0.0648 M. Addition of 25.5 equivalents of **8a** shows the conversion of **1** to **11a** (—) to (—).

Titration of $Rh_2(esp)_2$ with 8b

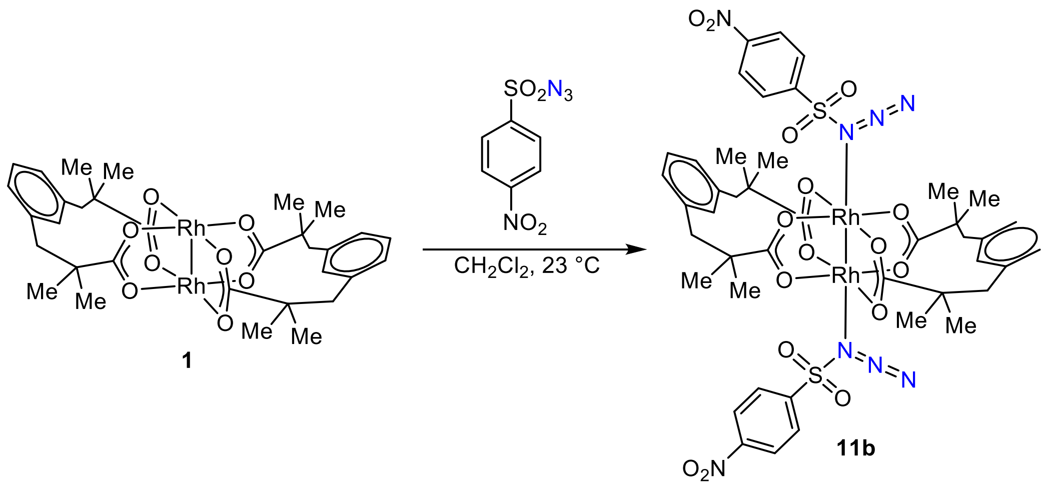

A 20-mL vial was charged with compound **1** (14.5 mg, 0.0191 mmol, 1.00 equiv.) and dissolved in CH_2Cl_2 (8.00 mL). Another 20-mL vial was charged with **8b** (47.3 mg, 0.207 mmol, 10.9 equiv.) and dissolved in CH_2Cl_2 (1.00 mL). A cuvette was charged with the stock solution of **1** (2.30 mL) and it was titrated with the stock solution of (0–0.0502 M) and monitored *via* UV-Vis until the spectrum stopped evolving (Figure S8).

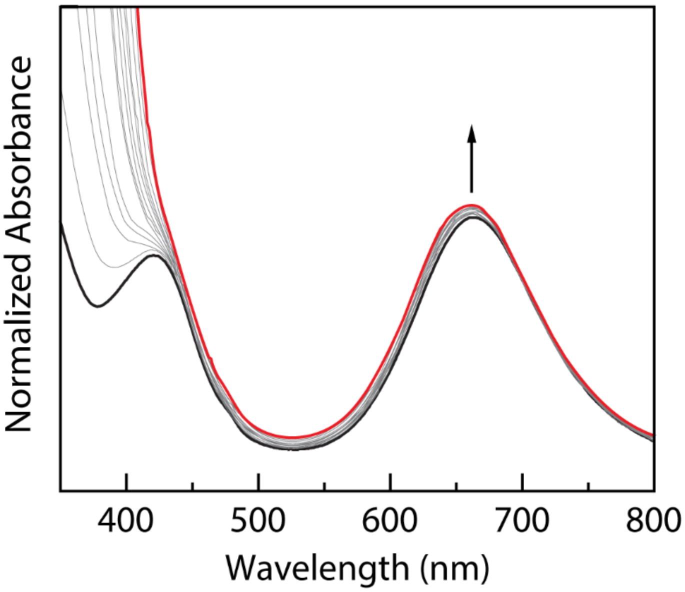

Figure S8. UV-vis spectra obtained during titration of **1** with **8b**. Spectra were collected in CH_2Cl_2 at 23 °C. The initial concentration of complex **1** was 0.00219 M. (a) UV-vis spectra collected at [**8b**] of 0–0.0502 M. Addition of 23.0 equivalents of **8b** shows the conversion of **1** to **11b** (—) to (—).

Titration of $Rh_2(esp)_2$ with 8c

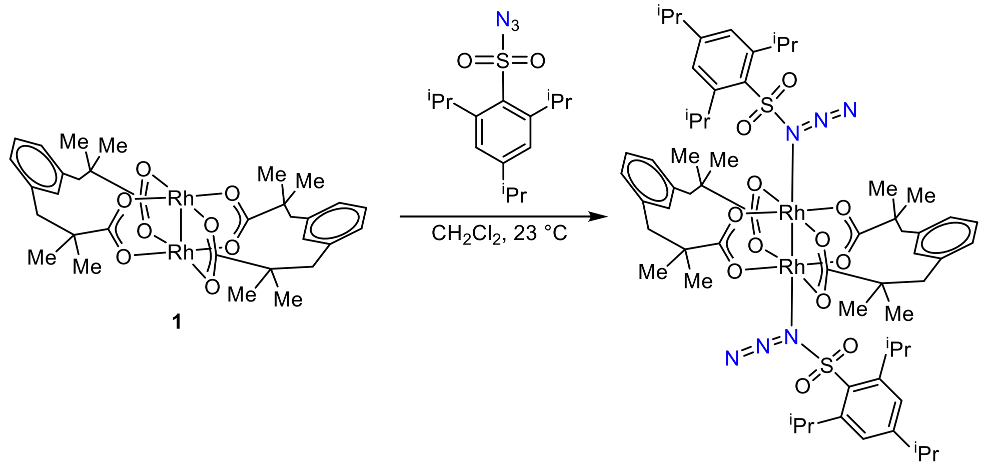

A 20-mL vial was charged with compound **1** (15.8 mg, 0.0208 mmol, 1.00 equiv.) and dissolved in CH_2Cl_2 (8.00 mL). Another 20-mL vial was charged with **8c** (63.9 mg, 0.206 mmol, 9.92 equiv.) and dissolved in CH_2Cl_2 (1.00 mL). A cuvette was charged with the stock solution of **1** (2.30 mL) and it was titrated with the stock solution of (0–0.0508 M) and monitored *via* UV-vis until the spectrum stopped evolving (Figure S9).

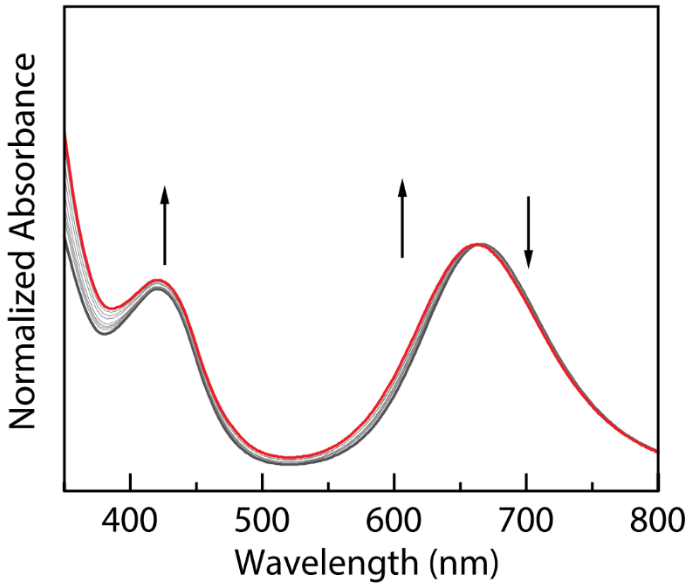

Figure S9. UV-vis spectra obtained during titration of **1** with **8c**. Spectra were collected in CH_2Cl_2 at 23 °C. The initial concentration of complex was 0.00261 M. (a) UV-vis spectra collected at [**8c**] of 0–0.0508 M. The well-anchored isosbestic point at 664 nm indicates the absence of steady-state intermediates in the conversion of **1** (—) to **11c** (—) upon addition of 19.5 equivalents of **8c**.

Titration of 9a with tetrahydrofuran

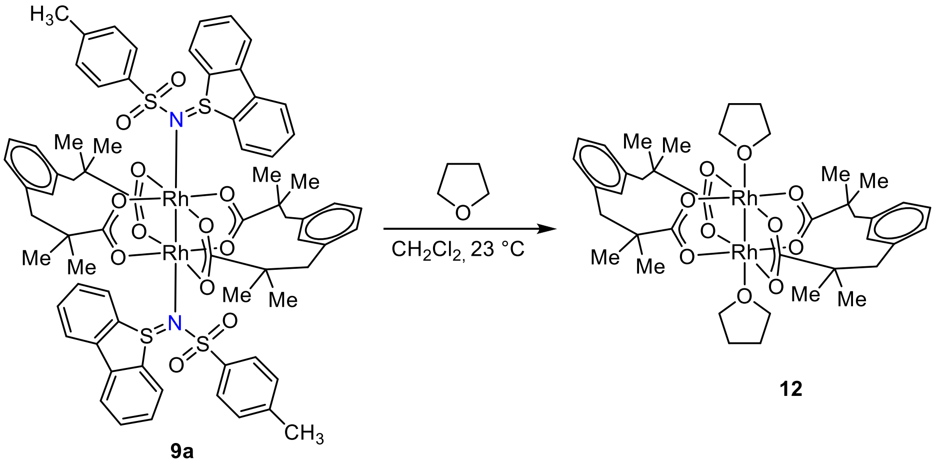

A 20-mL vial was charged with compound **1** (15.4 mg, 0.0203 mmol, 1.00 equiv.) and dissolved in CH_2Cl_2 (8.00 mL). A second 20-mL vial was charged with **6a** (15.2 mg, 0.0430 mmol, 2.11 equiv.) and dissolved in CH_2Cl_2 (1.60 mL). A cuvette was charged with the stock solution of **1** (2.10 mL) and stock solution of **6a** (0.400 mL) to prepare 2.50 mL solution of **9a** (0.00535 mmol, 1.00 equiv.) The formation of **9a** was confirmed by the UV-vis spectroscopy. A third 20-mL vial was charged with THF (0.05 mL, 0.616 mmol, 116 equiv.) and diluted in CH_2Cl_2 (4.00 mL). The solution of **9a** (2.50 mL) in the cuvette was titrated with the stock solution of THF (0–0.00804 M) and monitored *via* UV-vis until the spectrum stopped evolving (Figure S10).

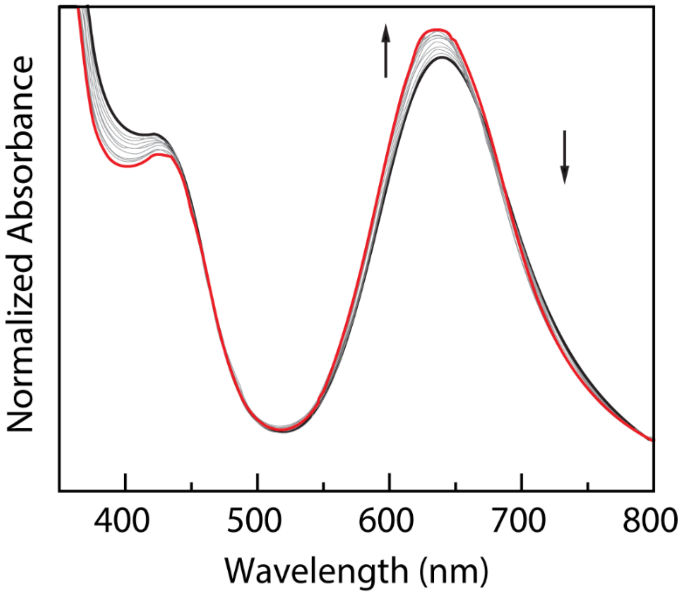

Figure S10. UV-vis spectra obtained during titration of **9a** with THF. Spectra were collected in CH_2Cl_2 at 23 °C. The initial concentration of **9a** was 0.00214 M. UV-vis spectra collected at [THF] of 0–0.00804 M. The well-anchored isosbestic points at 667 nm indicates the absence of steady-state intermediates in the conversion of **9a** (—) to **12** (—).

E. Ligand Exchange Experiment

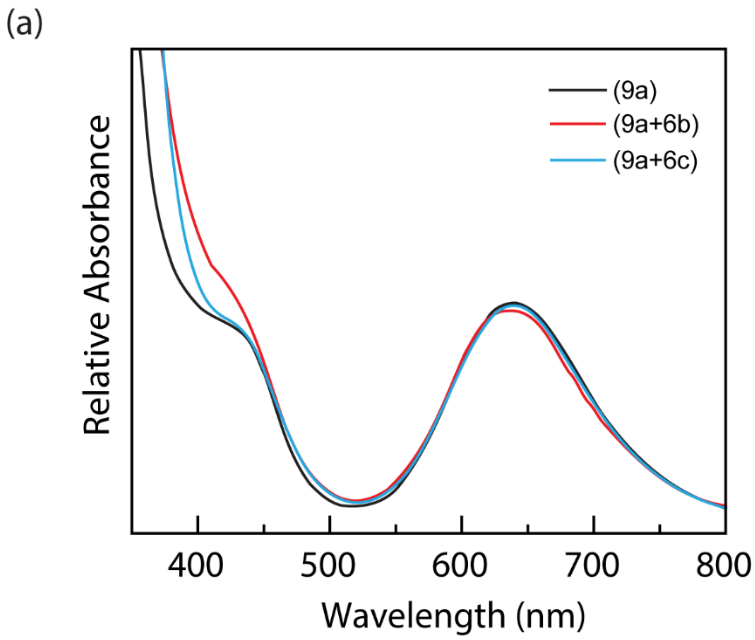

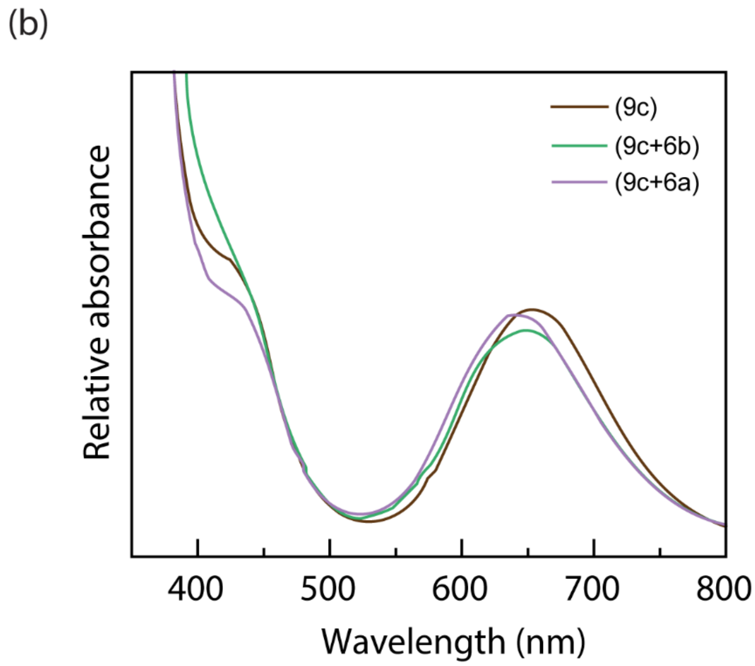

Figure S11. (a) Relative absorbance of compound **9a** (—) or equimolar mixtures of compound **9a** with **6b** (—) and **6c** (—) in dichloromethane. No significant changes were observed for the low-energy absorbance of **9a** upon addition of either **6b** or **6c.** (b) Relative absorbance of compound **9c** (—) or equimolar mixtures of compound **9c** with **6b** (—) and **6a** (—) in dichloromethane. The low energy band of **9c** at 655 nm shows a significant blue shift to 644 nm and 640 nm upon addition of **6b** and **6a**, respectively.

F. Solution-Phase Photolysis Experiments

Photolysis of Compound 9d

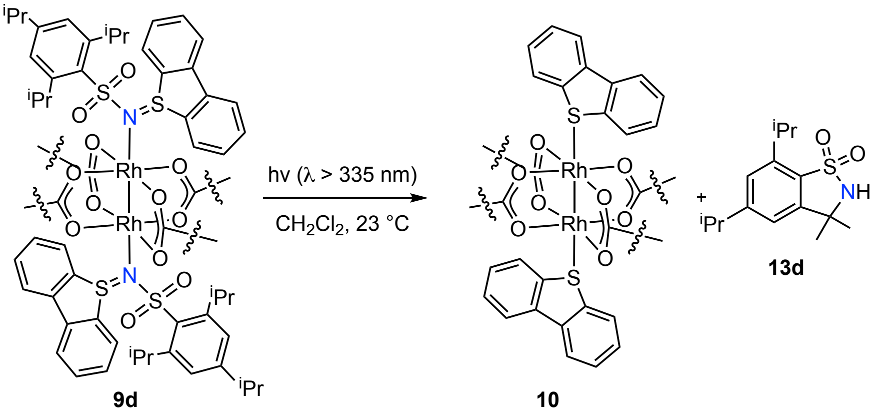

A 20-mL vial was charged with compound **1** (4.70 mg, 0.00619 mmol, 1.00 equiv.), **6d** (6.00 mg, 0.0124 mmol, 2.00 equiv.), and CH_2Cl_2 (3.00 mL) to prepare a stock solution. A screw-capped quartz cuvette was charged with 2.50 mL of the stock solution inside an N_2-filled glovebox. The solution was photolyzed (λ > 335 nm) and the reaction was monitored *via* UV-vis until the spectrum stopped evolving (Figure S12). After completion, the solution was split into two fractions. From one fraction, the solvent was removed *in vacuo* and the residue was taken up in $CDCl_3$ for ^1H NMR analysis (Figure S12). From the other fraction, single crystals of **10** were obtained from concentrated CH_2Cl_2 with pentane layering at 0 °C. The crystals were washed pentane and dried *in vacuo.* Crystals were dissolved in $CDCl_3$ for ^1H NMR analysis (Figure S13).

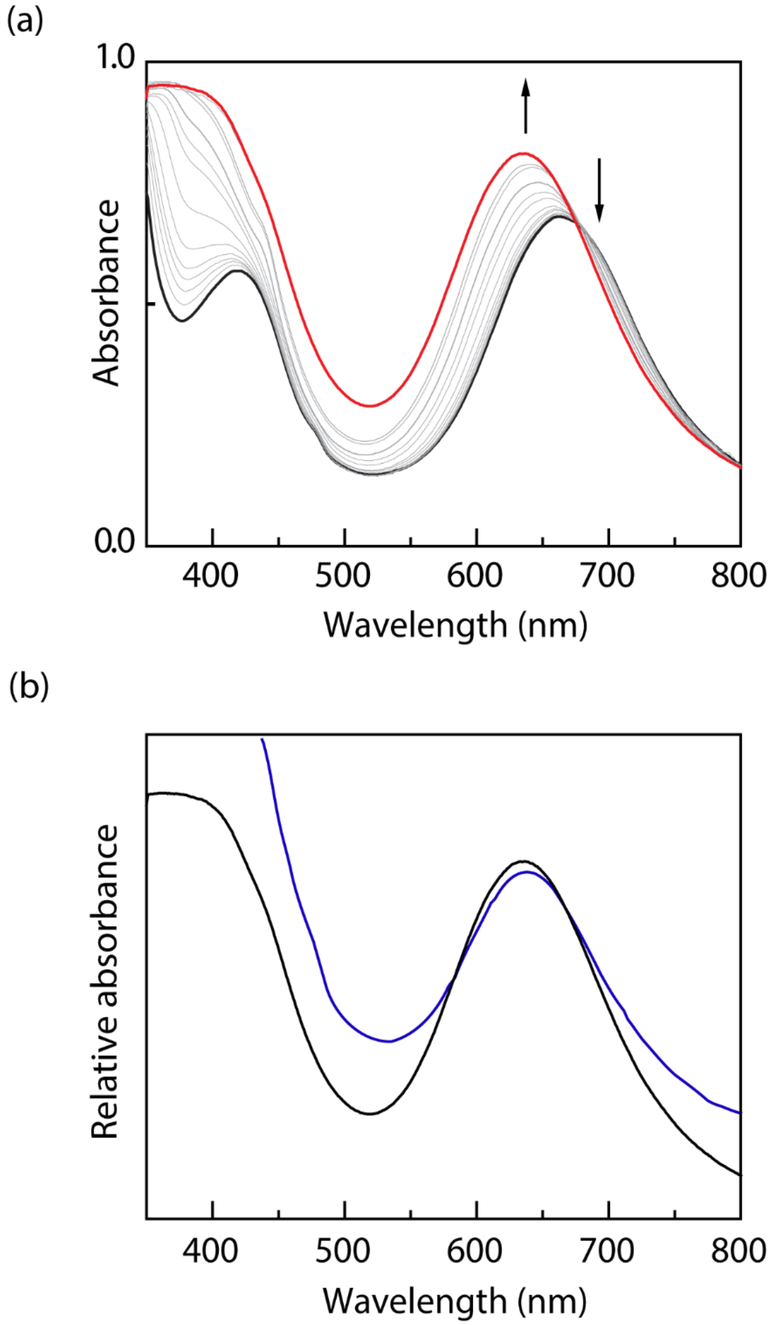

Figure S12. (a) UV–vis spectra collected during the photolysis (λ > 335 nm) of compound **9d** in CH_2Cl_2. A well-anchored isosbestic point is observed at 675 nm, which indicates the lack of a steady-state intermediate in the conversion of **9d** to **10** and **13d**. (b) Comparison of UV-vis spectra of the end point of the photolysis of compound **9d** (—) and compound **10** (—) in CH_2Cl_2.

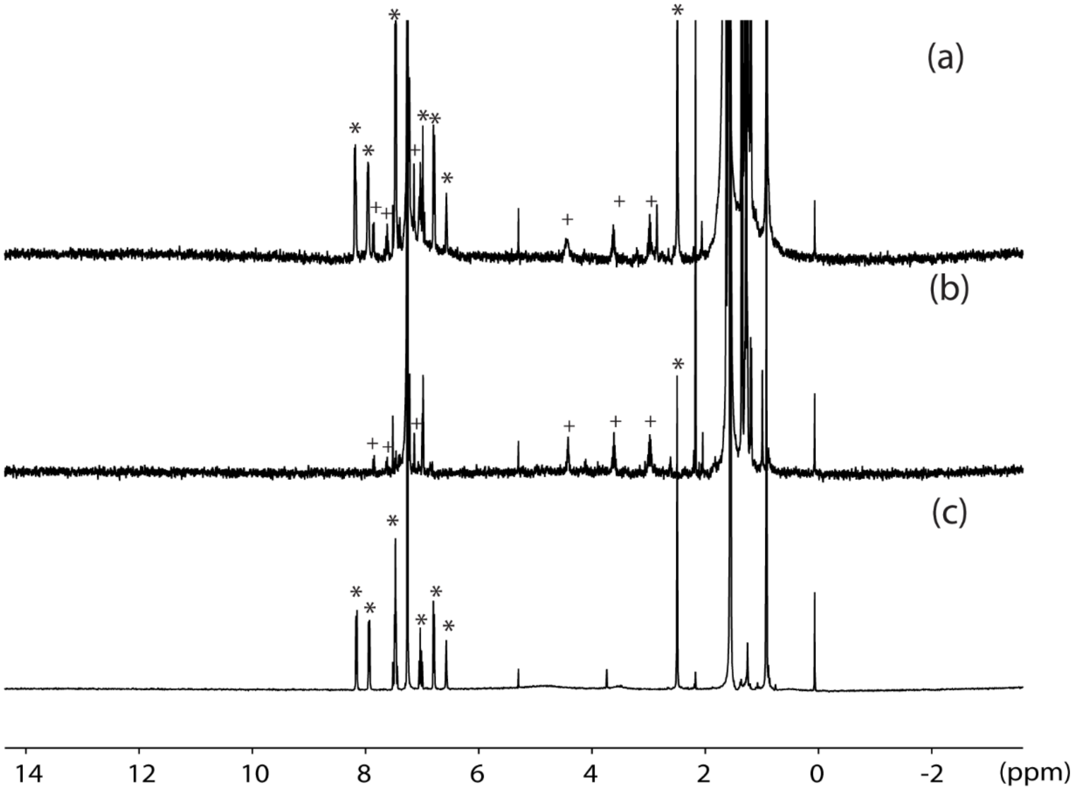

Figure S13. Photolysis (λ > 335 nm) of compound **9d** in CH_2Cl_2 for 24 h results in the formation of compound **13d** and **10**. (a) ^{1}H NMR of the crude mixture of compound **9d** after 24 h photolysis; (b) ^{1}H NMR of isolated compound **13d**; (c) ^{1}H NMR of compound **10** measured at 400 MHz in $CDCl_3$ at 23 °C. The formation compound **13d** and **10** in (a) indicated by + and * signs respectively.

Photolysis of Compound 9e

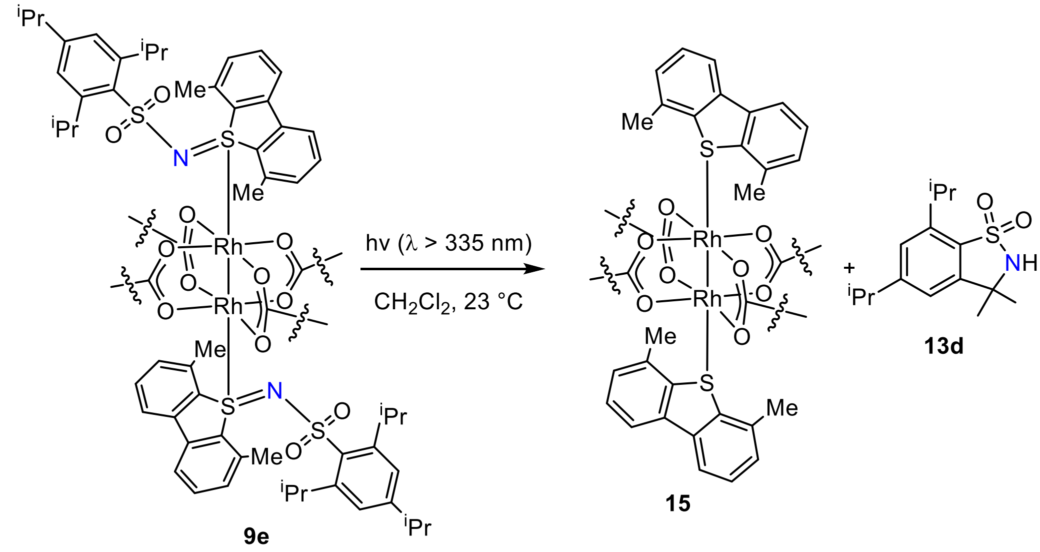

A 20-mL vial was charged with compound **9e** (12.2 mg, 0.00699 mmol, 1.00 equivalent and CH_2Cl_2 (3.00 mL) to prepare a stock solution. A screw-capped quartz cuvette was charged with 2.50 mL of the stock solution inside an N_2-filled glovebox. The solution was photolyzed (λ > 335 nm) and the reaction was monitored *via* UV-vis until the spectrum stopped evolving (Figure S14). After completion the solvent was removed *in vacuo* and the residue was taken up in CD_2Cl_2 for ^1H NMR analysis (Figure S15).

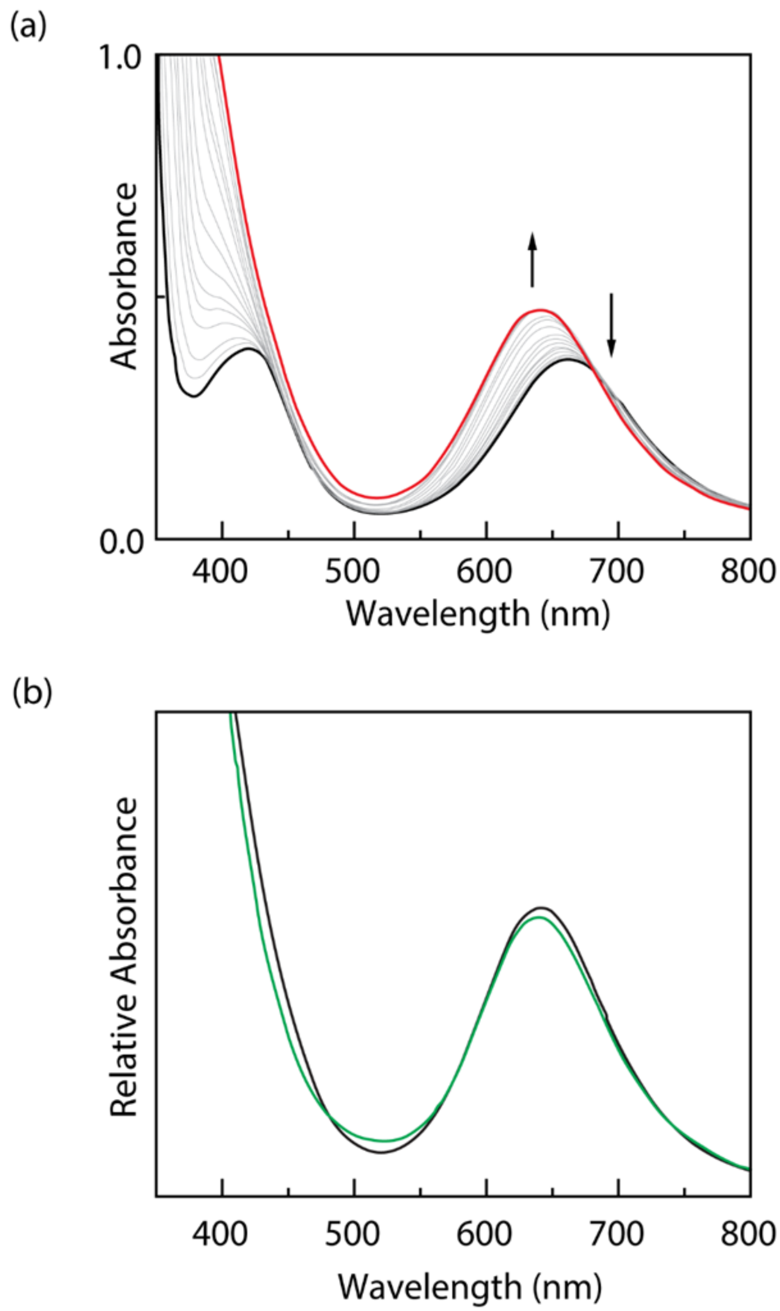

Figure S14. (a) UV-vis spectra were collected during the photolysis (λ > 335 nm) of compound **9e** in CH_2Cl_2. A well-anchored isosbestic point is observed at 682 nm, which indicates the lack of a steady-state intermediate in the conversion of **9e** to **15** and **13d**. (b) Comparison of UV-vis spectra of the endpoint of the photolysis of compound **9e** (**—**) and compound **15** (—) in CH_2Cl_2.

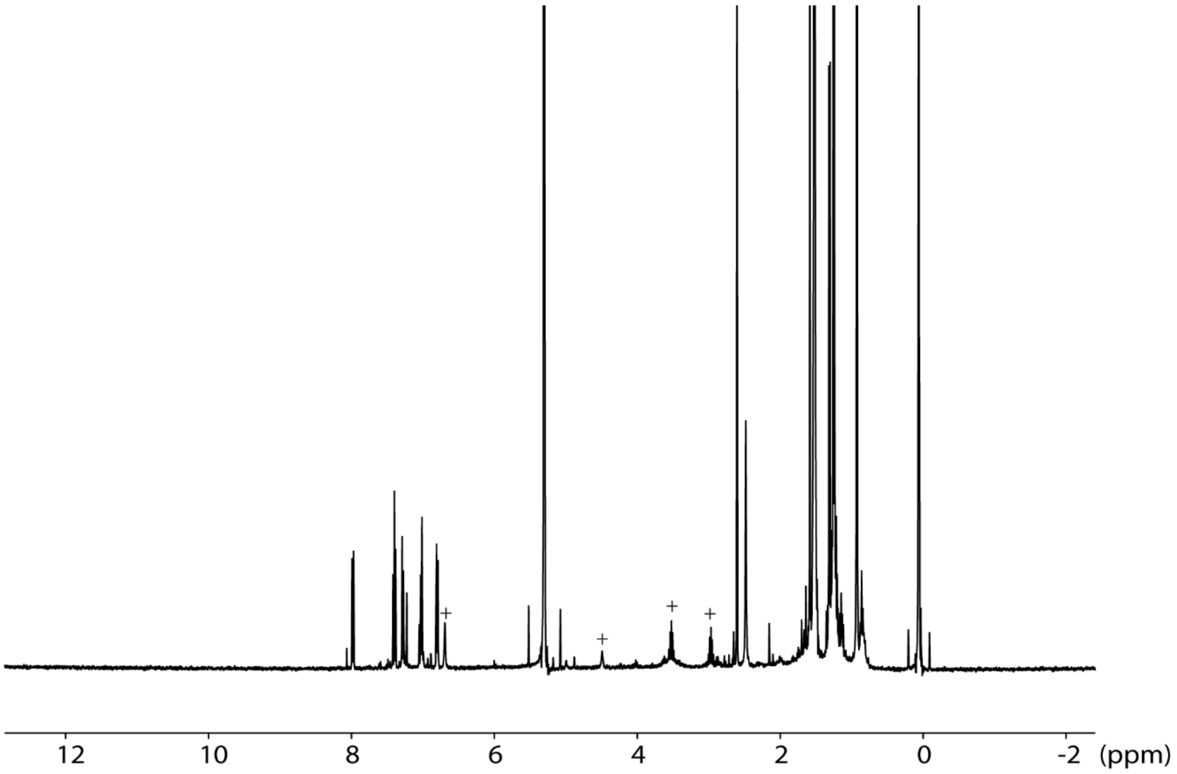

Figure S15. Photolysis (λ > 335 nm) of compound **9e** in CH_2Cl_2 for 24 h results in the formation of compounds **13d** and **15**. ^1H NMR of the crude mixture of compound **9e** after 24 h photolysis measured at 400 MHz in CD_2Cl_2 at 23 °C. Formation of **13d** indicated by +.

Photolysis of compound 9a in the Presence of Tetralin

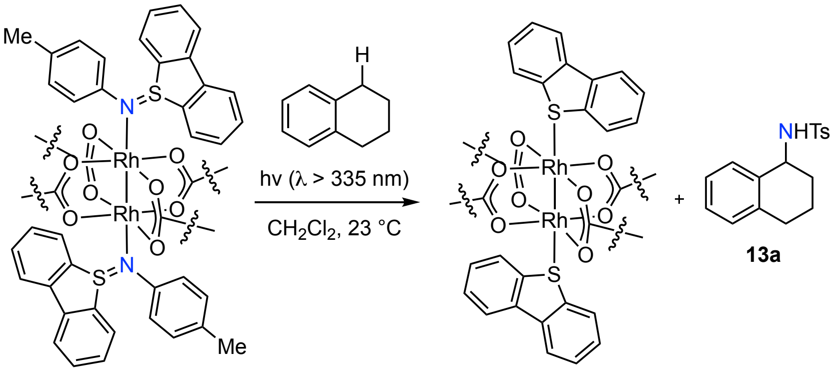

9a **10**

A 20-mL vial was charged with compound **1** (10.4 mg, 0.0137 mmol, 1.00 equiv.) and **6a** (10.2 mg, 0.0289 mmol, 2.10 equiv.), 0.20 mL tetralin (0.734 mmol, 107 equiv.), and CH_2Cl_2 (6.00 mL) to prepare a stock solution. A screw-capped quartz cuvette was charged with 2.50 mL of the stock solution inside an N_2 filled glovebox. The solution was photolyzed (λ > 335 nm) and the reaction was monitored *via* UV-vis until the spectrum stopped evolving. The solvent was then removed *in vacuo* and the residue was taken up in $CDCl_3$ for NMR analysis (Figure S17).

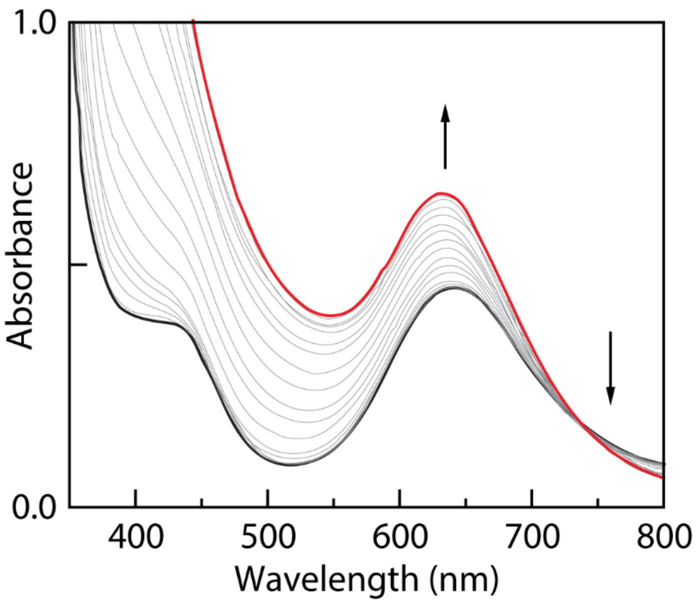

Figure S16. UV–vis spectra collected during the photolysis (λ > 335 nm) of compound **9a** with tetralin in CH_2Cl_2. A well-anchored isosbestic point is observed at 740 nm, which indicates the lack of a steady-state intermediate in the conversion of **9a** to **10** and **13a**.

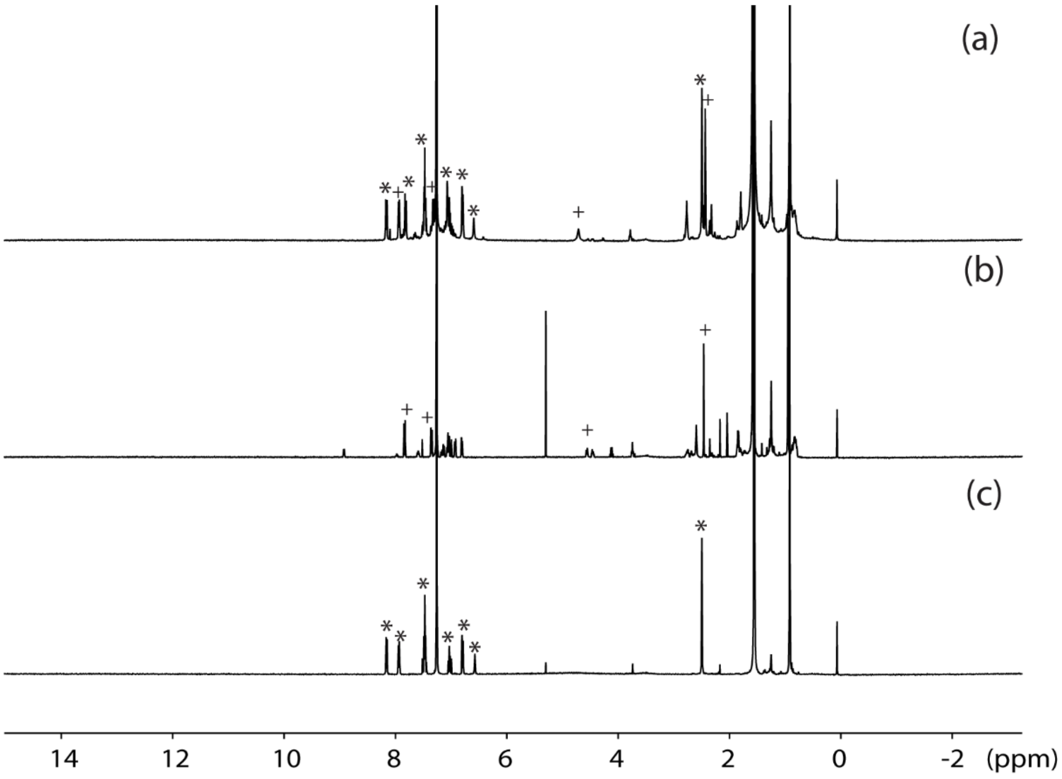

Figure S17. Photolysis (λ > 335 nm) of compound **9a** in the presence of tetralin in CH_2Cl_2 for 24 h results in the formation of compound **13a** and **10**. (a) ^{1}H NMR of the crude mixture of compound **9a** and tetralin after 24 h photolysis; (b) ^{1}H NMR of isolated compound **13a**; (c) ^{1}H NMR of compound **10** measured at 400 MHz in $CDCl_3$ at 23 °C. The formation compound **13a** and **10** in (a) indicated by + and * signs respectively.

Photolysis of Compound 9b in the Presence of Tetralin

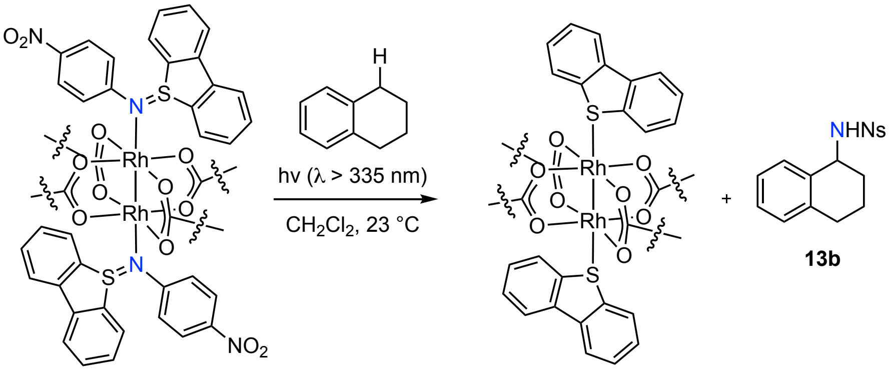

9b **10**

A 20-mL vial was charged with compound **1** (4.10 mg, 0.00540 mmol, 1.00 equiv.) and **6b** (4.50 mg, 0.0117 mmol, 2.17 equiv.), 0.100 mL tetralin (0.734 mmol, 136 equiv.), and CH_2Cl_2 (3.00 mL) to prepare a stock solution. A screw-capped quartz cuvette was charged with 2.50 mL of the stock solution inside an N_2 filled glovebox. The solution was photolyzed (λ > 335 nm) and the reaction was monitored *via* UV-vis until the spectrum stopped evolving. The solvent was then removed *in vacuo* and the residue was taken up in $CDCl_3$ for NMR analysis (Figure S19).

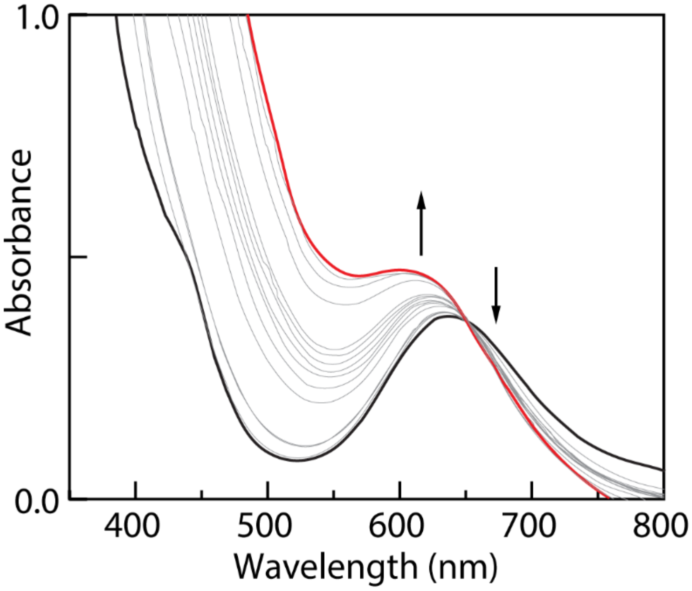

Figure S18. UV–vis spectra collected during the photolysis (λ > 335 nm) of compound **9b** with tetralin as substrate in CH_2Cl_2. A well-anchored isosbestic point is observed at 650 nm, which indicates the lack of a steady-state intermediate in the conversion of **9b** to **10** and **13b**.

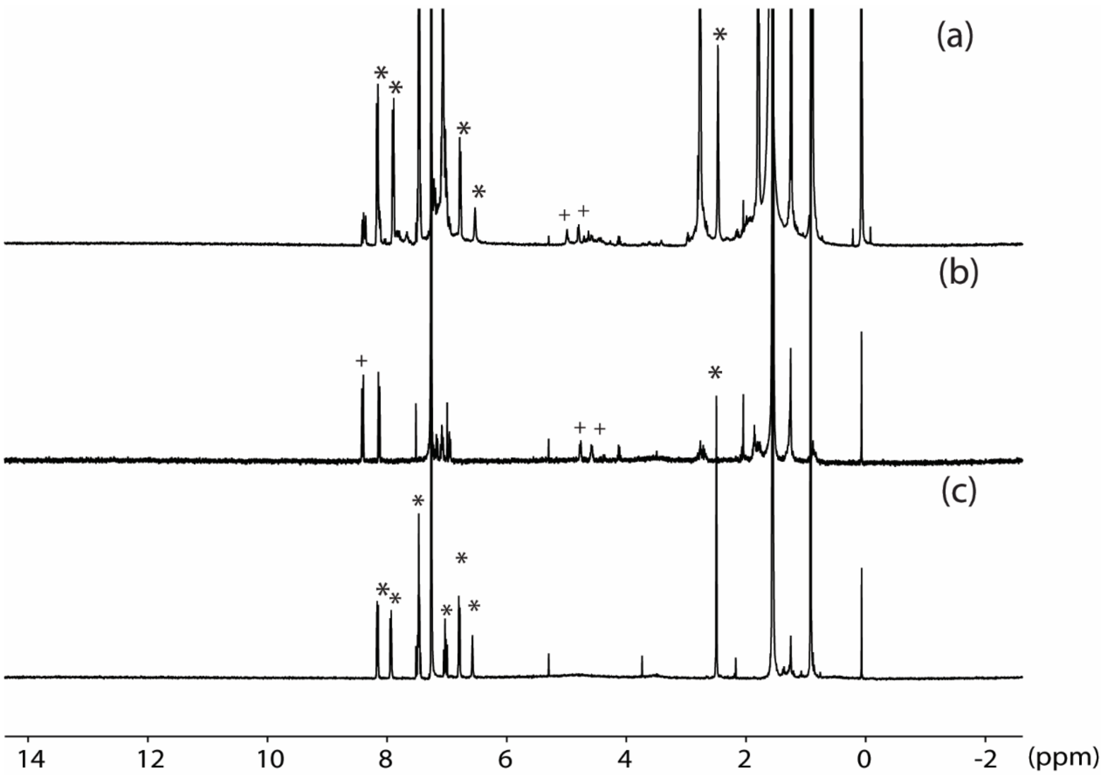

Figure S19. Photolysis (λ > 335 nm) of compound **9b** in the presence of tetralin in CH_2Cl_2 for 24 h results in the formation of compound **13b** and **10**. (a) ^1H NMR of the crude mixture of compound **9b** and tetralin after 24 h photolysis; (b) ^1H NMR of isolated compound **13b**; (c) ^1H NMR of compound **10** measured at 400 MHz in $CDCl_3$ at 23 °C. The formation compound **13b** and **10** in (a) indicated by + and * signs respectively.

Photolysis of Compound 9c in the Presence of Tetralin

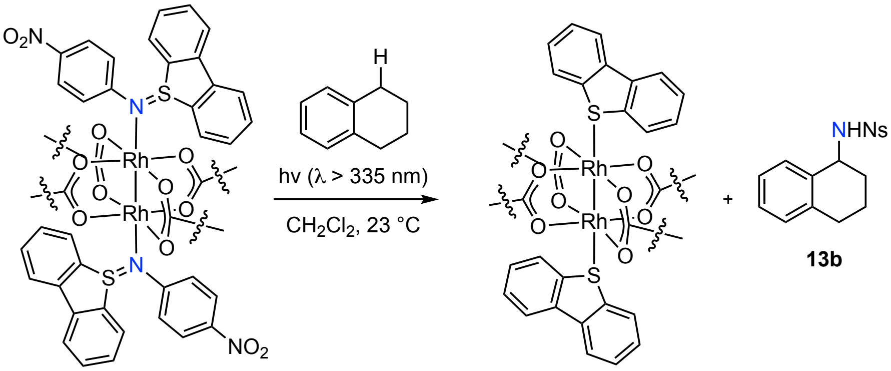

A 20-mL vial was charged with compound **1** (5.00 mg, 0.00659 mmol, 1.00 equiv.) and **6c** (6.10 mg, 0.0142 mmol, 2.15 equiv.), 0.10 mL tetralin (0.734 mmol, 111 equiv.), and CH_2Cl_2 (3.00 mL) to prepare a stock solution. A screw-capped quartz cuvette was charged with 2.50 mL of the stock solution inside an N_2-filled glovebox. The solution was photolyzed (λ > 335 nm) and the reaction was monitored *via* UV-vis until the spectrum stopped evolving. The solvent was then removed *in vacuo* and the residue was taken up in $CDCl_3$ for NMR analysis (Figure S21).

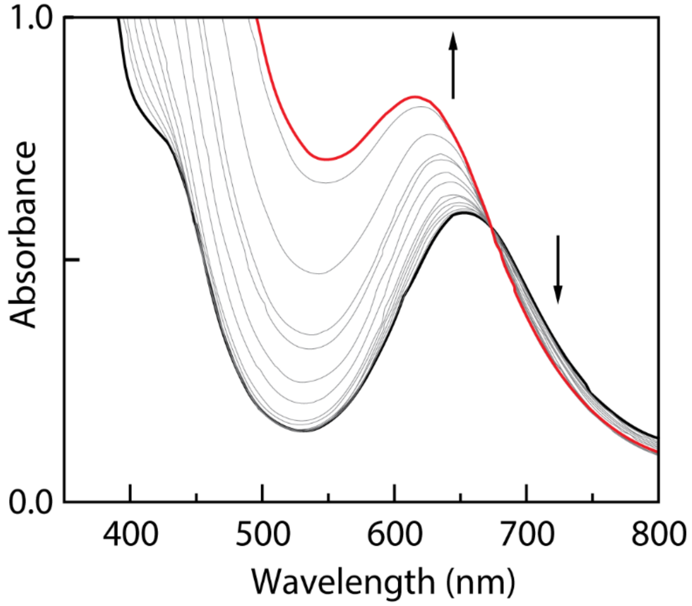

Figure S20. UV-vis spectra collected during the photolysis (λ > 335 nm) of compound **9c** with tetralin in CH_2Cl_2. A well-anchored isosbestic point is observed at 672 nm, which indicates the lack of a steady-state intermediate in the conversion of **9c** to **10** and **13c**.

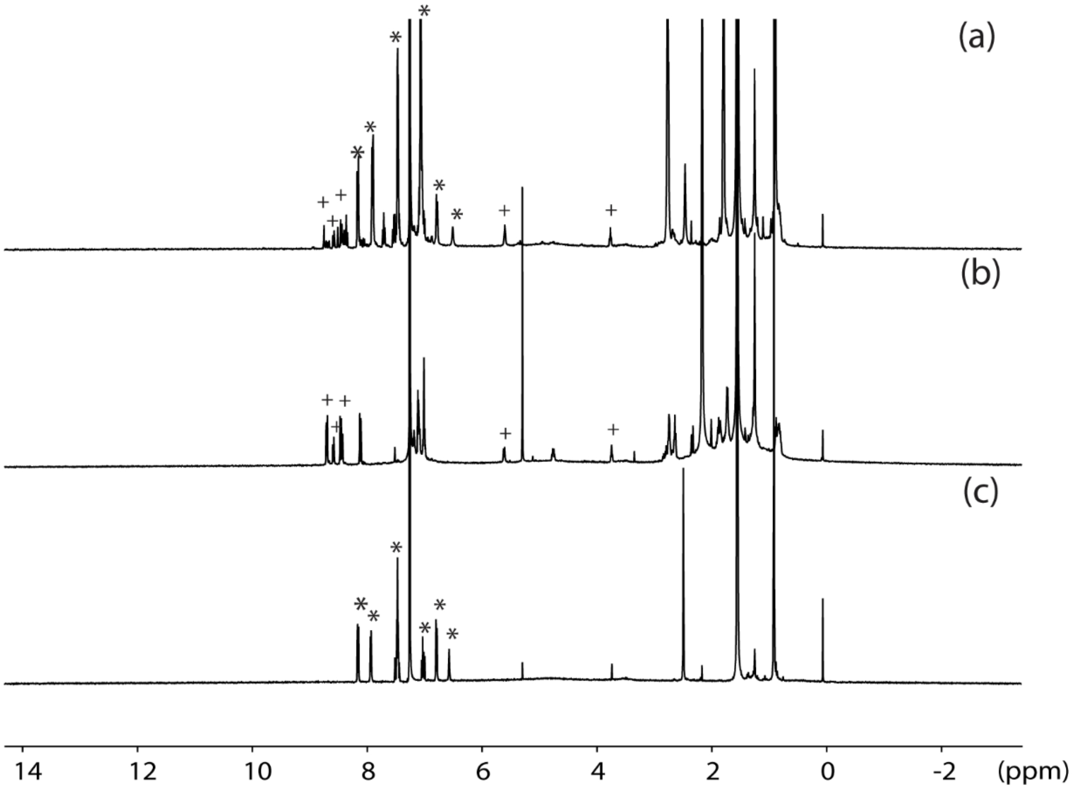

Figure S21. Photolysis (λ > 335 nm) of compound **9c** in the presence of tetralin in CH_2Cl_2 for 24 h results in the formation of compound **13b** and **10**. (a) ^1H NMR of the crude mixture of compound **9c** and tetralin after 24 h photolysis; (b) ^1H NMR of isolated compound **13c**; (c) ^1H NMR of compound **10** measured at 400 MHz in $CDCl_3$ at 23 °C. The formation compound **13b** and **10** in (a) indicated by + and * signs respectively.

G. Solid-State Photolysis Experiment

Photolysis of compound 9d

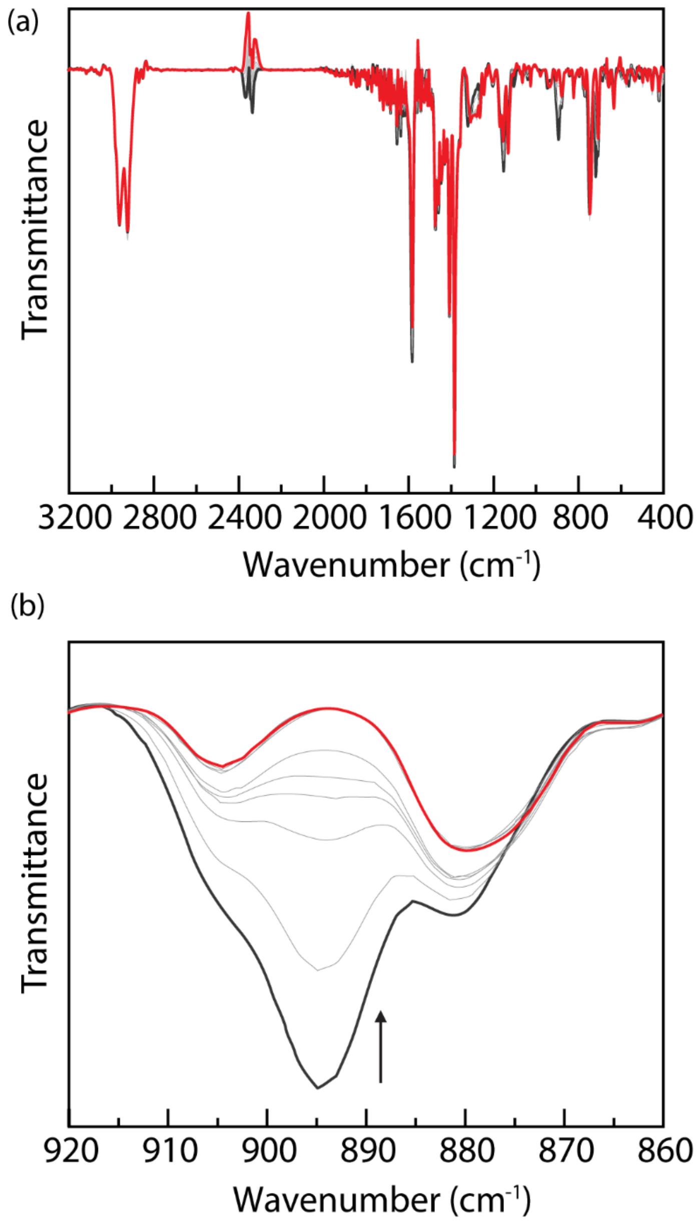

Figure S22. Solid-state photolysis of compound **9d**. (a) IR spectra collected during the photolysis (λ > 335 nm) of a KBr pellet of **9d** at 23 °C from 0 min (—) to 24 h (—). (b) Expansion of the spectral window depicting the disappearance of the N=S peak 896 cm^{-1}.

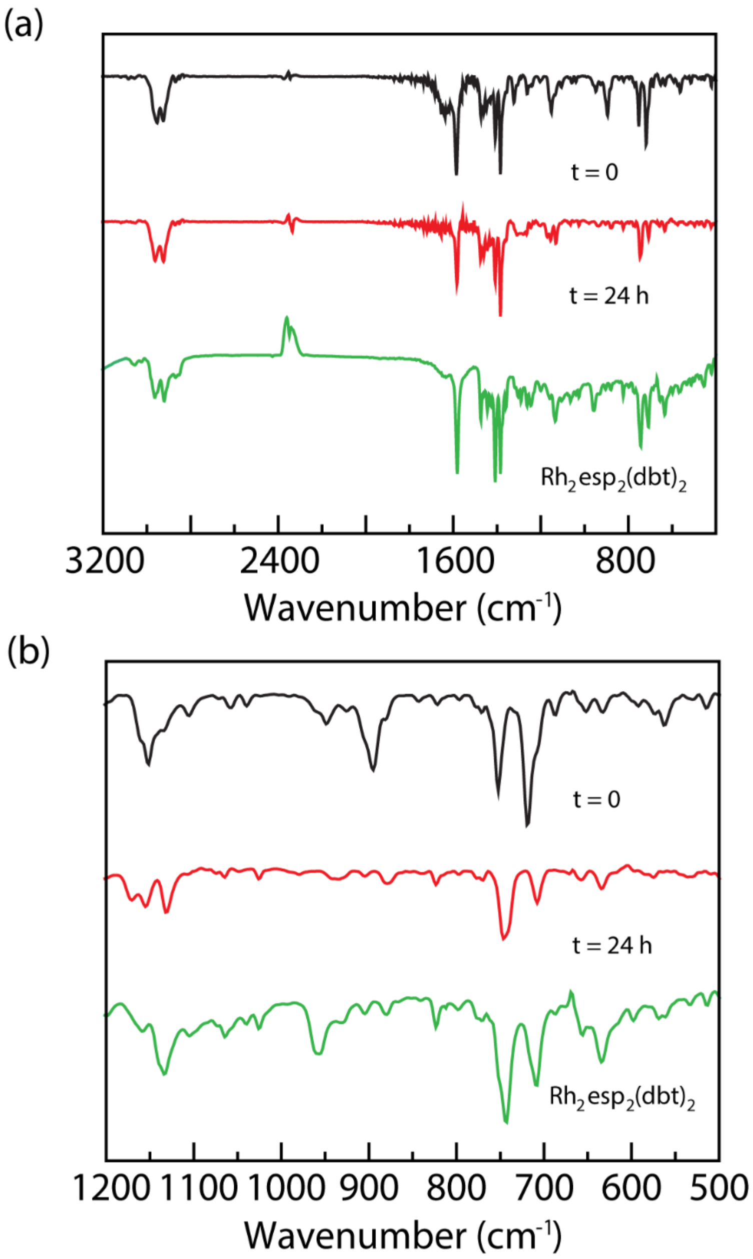

Figure S23. (a) Comparison of IR spectra of compound **9d** (—), following photolysis (—), and $Rh_2esp_2(dbt)_2$ (**10**) (—) (b) Expansion of the spectral window depicting the disappearance of the peaks at 896 cm^{-1} and redshift of the peaks at 752 and 719 cm^{-1} to 744 and 709 cm^{-1} from t = 0 to t = 24 h. The comparison between (—) and (—) indicates the formation of **10** during 24 h photolysis.

Photolysis of compound 9b

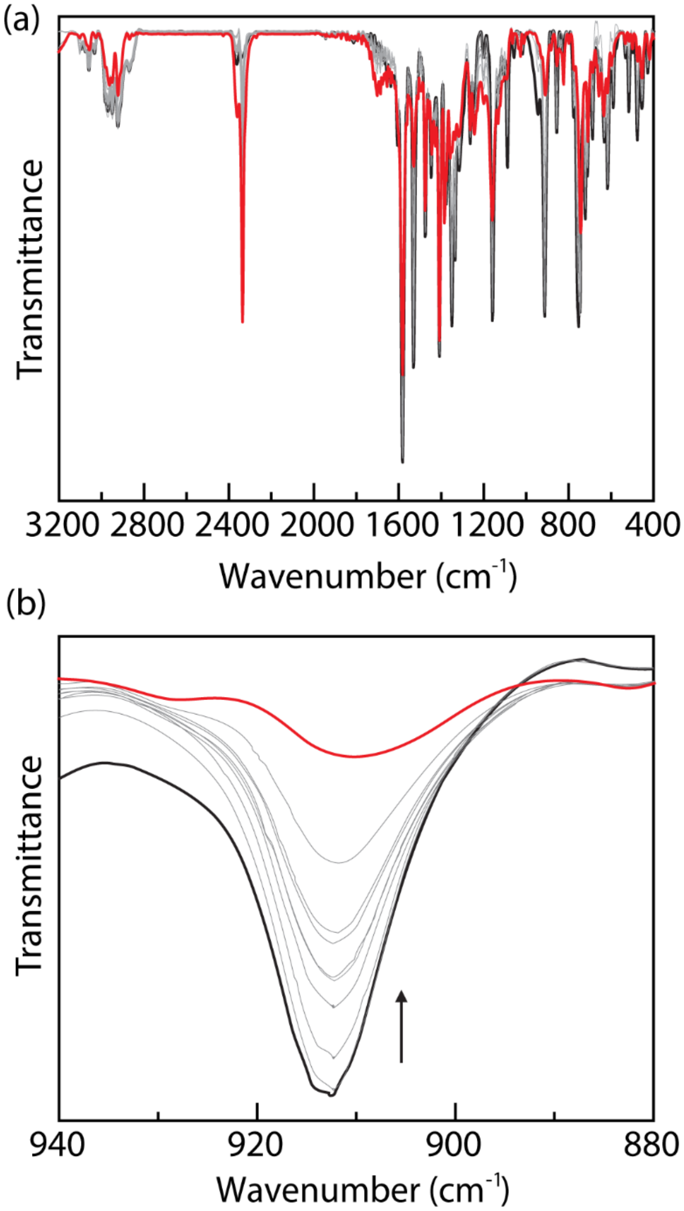

Figure S24. Solid-state photolysis of compound **9b**. (a) IR spectra collected during the photolysis (λ > 335 nm) of a KBr pellet of **9b** at 23 °C from 0 min (—) to 24 h (—). (b) Expansion of the spectral window depicting the disappearance of the N=S peak 912 cm^{-1}.

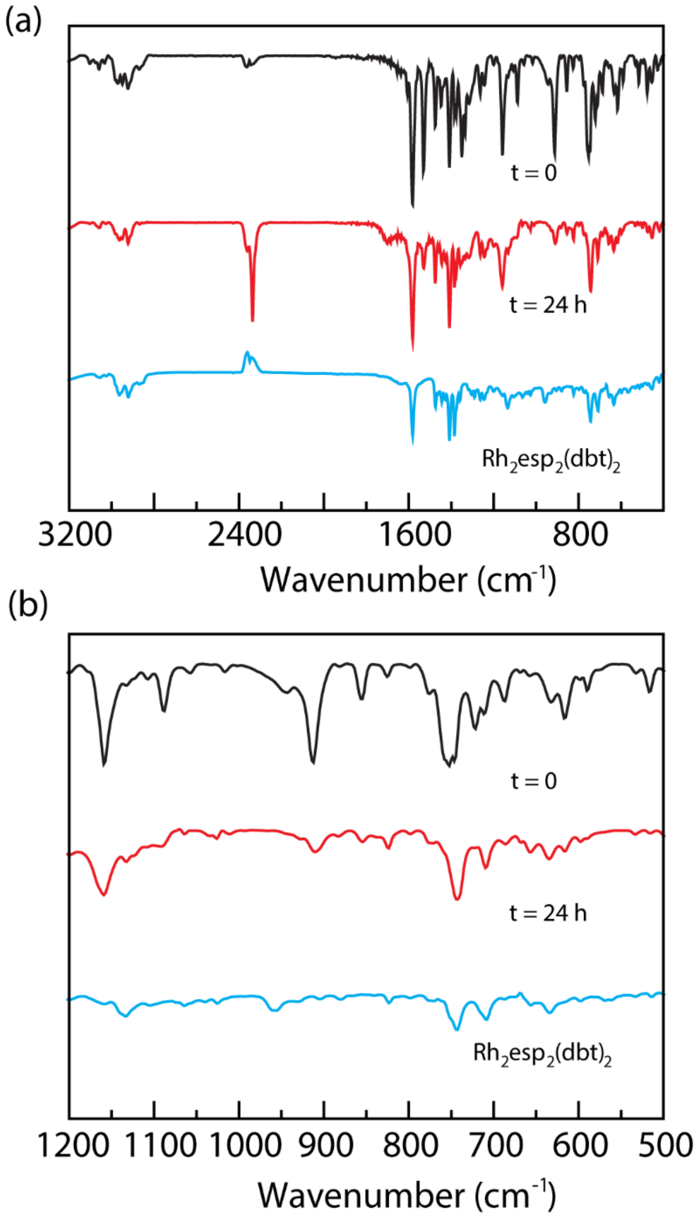

Figure S25. (a) Comparison of IR spectra of compound **9b** (—), following photolysis (—), and $Rh_2esp_2(dbt)_2$ (**10**) (—). (b) Expansion of the spectral window depicting the disappearance of the peaks at 912, 856 and 723 cm^{-1} and redshift of the peaks at 752, 711 cm^{-1} to 744 and 709 cm^{-1}. The comparison between (—) and (—) indicates the formation of (**10**) following photolysis.

Photolysis of compound 9e

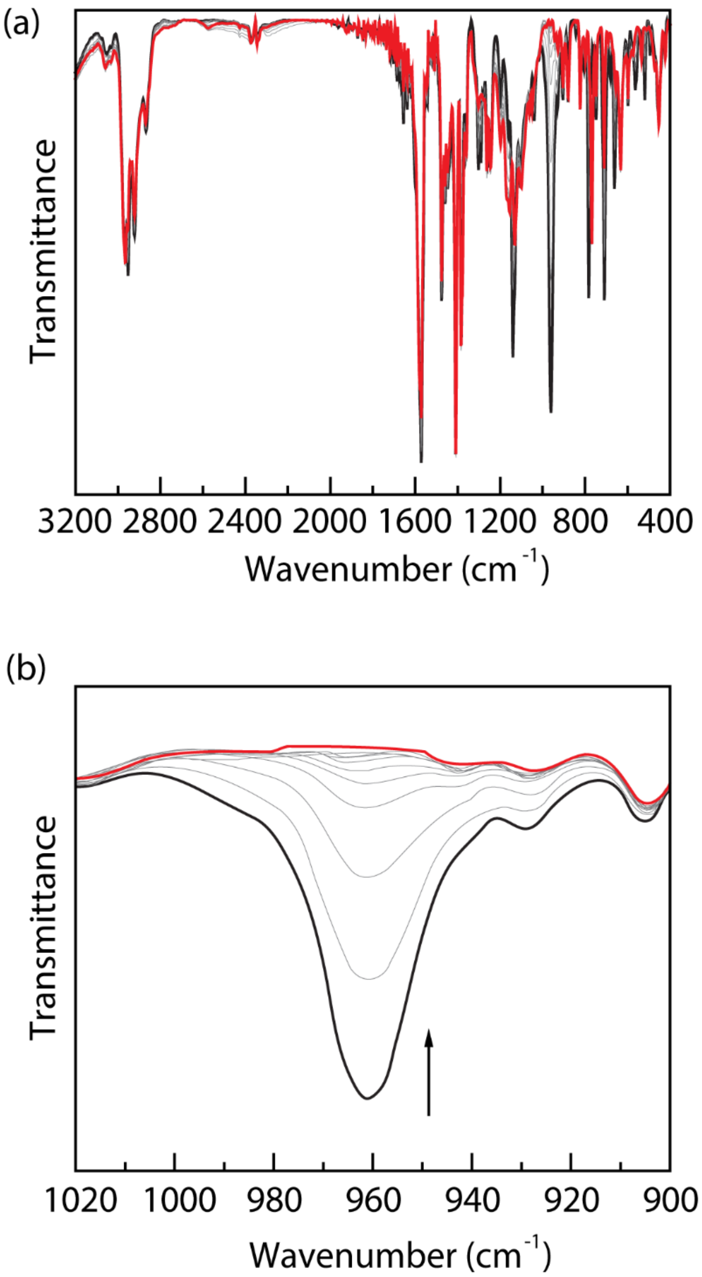

Figure S26. Solid-state photolysis of compound **9e**. (a) IR spectra collected during the photolysis (λ > 335 nm) of a KBr pellet of **9e** at 23 °C from 0 min (—) to 11 h (—). (b) Expansion of the spectral window depicting the disappearance of the N=S peak 960 cm^{-1}.

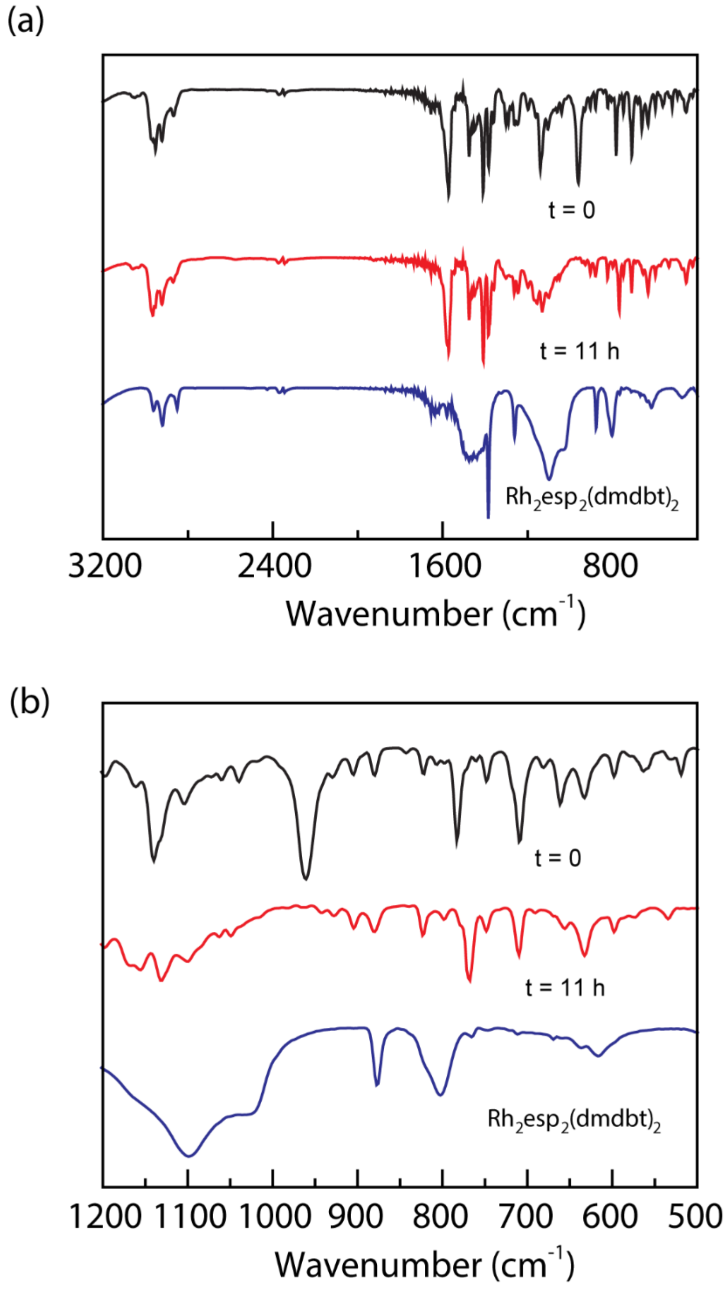

Figure S27. (a) Comparison of IR spectra of compound **9e** (—), following photolysis (—), and $Rh_2esp_2(dmdbt)_2$ (**15**) (—) (b) Expansion of the spectral window depicting the disappearance of the peaks at disappearance of the peaks at 960, 806, 680 cm^{-1} and redshift of the peak at 783 cm^{-1} to 767 cm^{-1} from t = 0 to t = 11 h. The comparison between (—) and (—) indicates the formation of **15** during 11 h photolysis.

H. Thin-Film Photochemistry

Preparation of thin film of compound 9d

A 20-mL vial was charged with polystyrene (Mw = 350000; 125 mg) and dry 1,2-dichloroethane (5.00 mL) and was sonicated for 15 min to afford a 2 wt% stock solution of polystyrene. Another second vial was charged with **1** (8.10 mg, 0.0107 mmol, 1.0 equiv.), compound **6d** (10.1 mg, 0.0217 mmol, 2.02 equiv.), and 2.50 mL of the polystyrene stock solution and was sonicated for 10 min to make a solution of **9d**.[a] A sapphire slide was washed with hexanes followed by sonication in 2-propanol for 15 min and was dried in a 100 °C oven overnight. The thin-film was drop-cast on a clean and dry sapphire slide, where the residual 1,2-dichloroethane was removed *in vacuo* for 24 h.

Preparation of the thin film of compound $Rh_2esp_2(dbt)_2$ (10)

A 20-mL vial was charged with polystyrene (Mw 350000; 125 mg) and dry 1,2-dichloroethane (5.00 mL) and was sonicated for 15 min to make a 2 wt% stock solution of polystyrene. Another vial was charged with **1** (11.0 mg, 0.0145 mmol, 1.00 equiv.) dibenzothiophene (5.40 mg, 0.0293 mmol, 2.02 equiv.), and 2.50 mL of the polystyrene stock solution and was sonicated for 10 min to make a solution of **10**.[b] A sapphire slide was washed with hexanes, followed by sonication in 2-propanol for 15 min, and was dried in a 100 °C oven overnight. The thin-film was drop-cast on a clean and dry sapphire slide, where the residual 1,2-dichloroethane was removed *in vacuo* for 24 h.

[a] The UV-vis spectrum of this polystyrene solution of **9d** was superimposable on a DCE solution of **9d**.
[b] The UV-vis spectrum of this polystyrene solution of **10** was superimposable on a DCE solution of **10**.

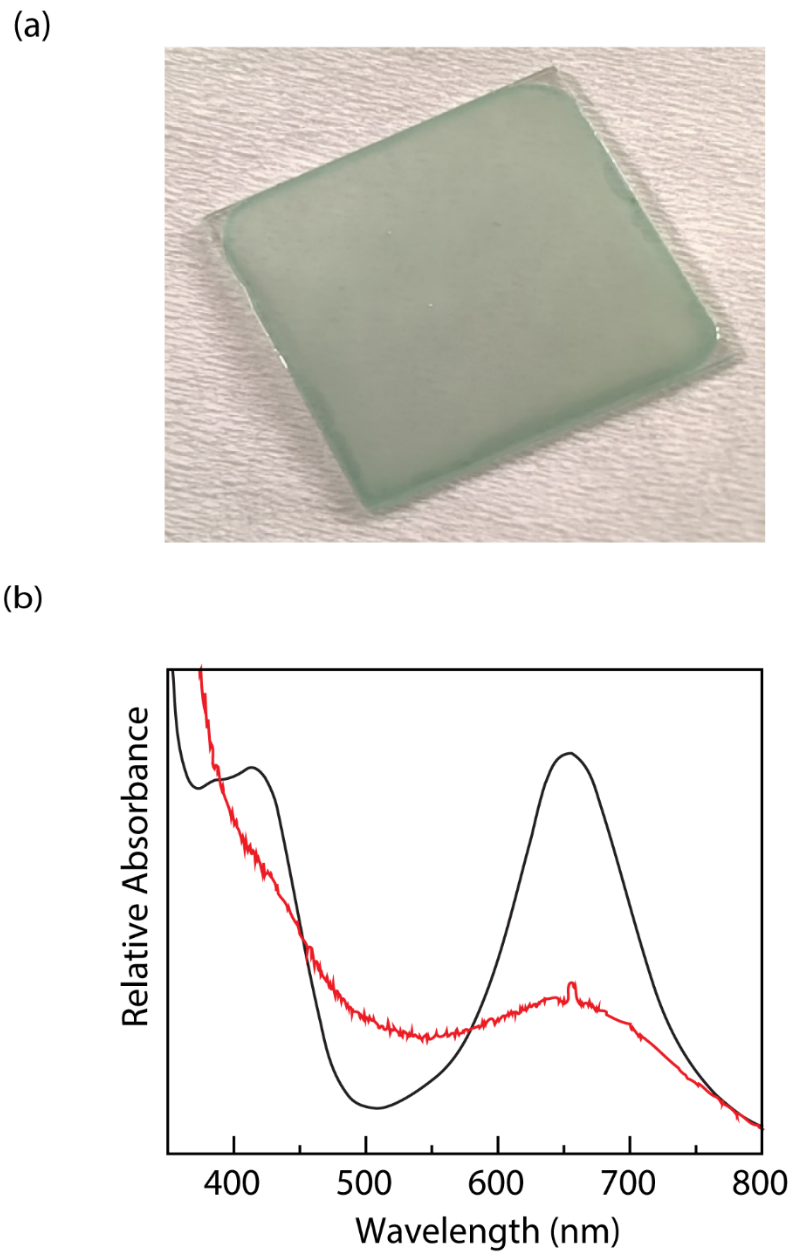

Figure S28. (a) Thin film of compound **9d** prepared from 2 wt% polystyrene (Mw = 350000) solution of 1,2-dichloroethane. (b) Comparison between the solution phase spectrum of **9d** (—) in 2 wt% polystyrene (Mw = 350000) in 1,2-dichloroethane and spectrum of compound **9d** in a thin film on a sapphire slide (—).

I. X-Ray Diffraction Data

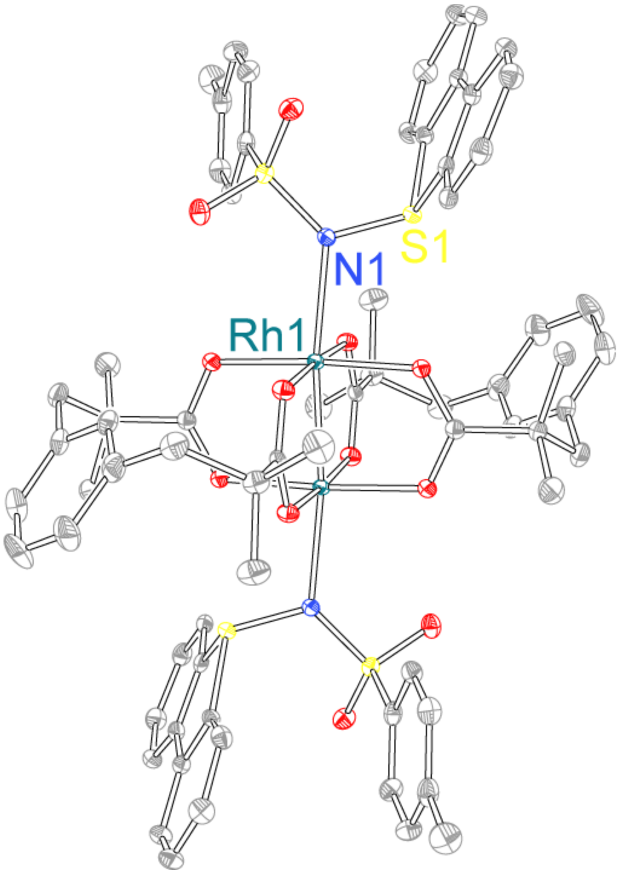

Figure S29. Displacement ellipsoid plot of **9a** plotted at 50% probability. H-atoms and solvents are removed for clarity. The crystalline sample used in this diffraction experiment was obtained from a concentrated CH_2Cl_2 solution at −20 °C.

Crystal data	
Chemical formula	$C_{70}H_{70}N_2O_{12}Rh_2S_4 \cdot 2(CH_2Cl_2)$
M_r	1635.19
Crystal system, space group	Orthorhombic, *Pbca*
Temperature (K)	110
a, *b*, *c* (Å)	19.1550(7), 17.2810(5), 21.6940(6)
V (Å^3)	7181.1(4)
Z	4
Radiation type	Mo $K\alpha$
μ (mm^{-1})	0.79
Crystal size (mm)	0.13 × 0.11 × 0.02
Data collection	
Diffractometer	Bruker Quest (PHOTON III)
Absorption correction	Multi-scan<br>*SADABS2016*/2 (Bruker,2016/2)
T_{min}, T_{max}	0.688, 0.746
No. of measured, independent and observed [$I > 2\sigma(I)$] reflections	108738, 10951, 8398
R_{int}	0.074
$(\sin \theta/\lambda)_{max}$ (Å^{-1})	0.715
Refinement	
$R[F^2 > 2\sigma(F^2)]$, $wR(F^2)$, S	0.037, 0.082, 1.06
No. of reflections	10951
No. of parameters	460
No. of restraints	86
H-atom treatment	H-atom parameters constrained
$\Delta\rho_{max}$, $\Delta\rho_{min}$ (e Å^{-3})	0.93, −0.82

Table S1. X-ray experimental details of **9a** (CCDC 2260853).

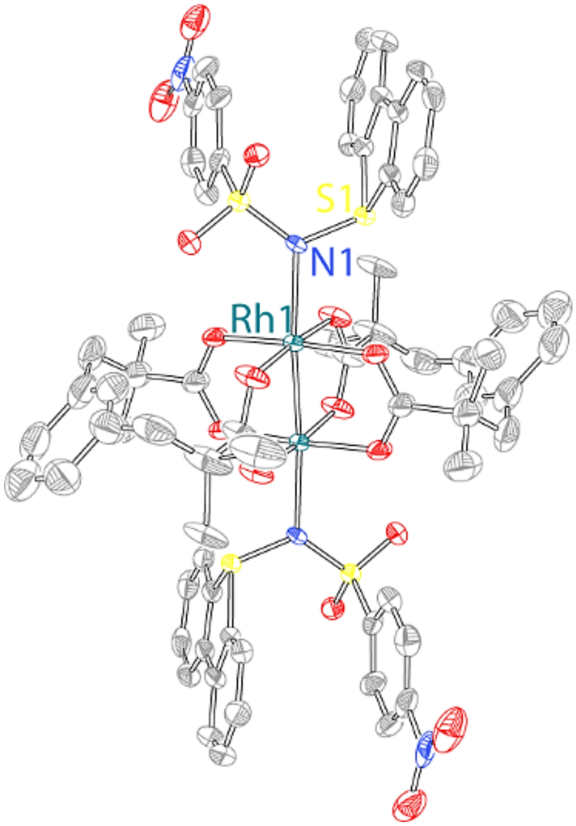

Figure S30. Displacement ellipsoid plot of **9b** plotted at 50% probability. H-atoms and solvents are removed for clarity. The crystalline sample used in this diffraction experiment was obtained from a concentrated CH_2Cl_2 solution at −20 °C.

Table S2. X-ray experimental details of **9b** (CCDC 2261080).

Crystal data	
Chemical formula	$C_{68}H_{64}N_4O_{16}Rh_2S_4{\cdot}CH_2Cl_2$
M_r	1612.21
Crystal system, space group	Monoclinic, $C2/c$
Temperature (K)	100
a, b, c (Å)	25.393(3), 14.204(2), 22.950(3)
β (°)	120.729(2)
V (Å^3)	7115.3(2)
Z	4
Radiation type	Synchrotron, λ = 0.41328 Å
μ (mm^{-1})	0.71
Crystal size (mm)	0.11 × 0.05 × 0.03
Data collection	
Diffractometer	Synchrotron
Absorption correction	Multi-scan<br>*SADABS2016*/2 (Bruker,2016/2)
T_{min}, T_{max}	0.661, 0.744
No. of measured, independent and observed [$I > 2\sigma(I)$] reflections	98178, 8195, 7222
R_{int}	0.053
$(\sin \theta/\lambda)_{max}$ (Å^{-1})	0.651
Refinement	
$R[F^2 > 2\sigma(F^2)]$, $wR(F^2)$, S	0.068, 0.173, 1.18
No. of reflections	8195
No. of parameters	536
No. of restraints	577
H-atom treatment	H-atom parameters constrained
$\Delta\rho_{max}$, $\Delta\rho_{min}$ (e Å^{-3})	1.83, −1.25

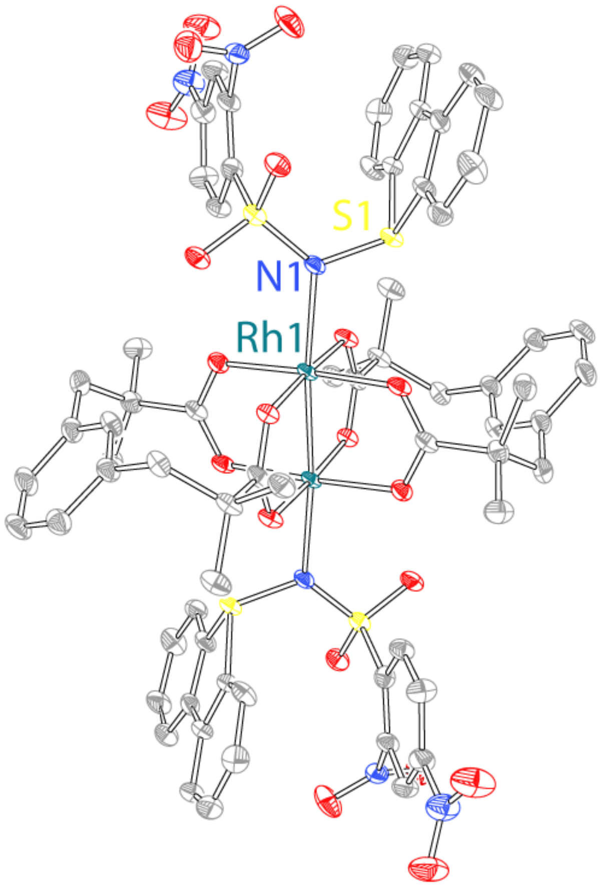

Figure S31. Displacement ellipsoid plot of **9c** plotted at 50% probability. H-atoms and solvents are removed for clarity. The crystalline sample used in this diffraction experiment was obtained from a concentrated CH_2Cl_2 solution at −20 °C.

Table S3. X-ray experimental details of **9c** (CCDC 2261071).

Crystal data	
Chemical formula	$C_{68}H_{62}N_6O_{20}Rh_2S_4 \cdot 4(CH_2Cl_2)$
M_r	1957.00
Crystal system, space group	Triclinic, *P*-1
Temperature (K)	110
a, *b*, *c* (Å)	10.7438(6), 13.7006(8), 14.4872(8)
α, β, γ (°)	102.721(2), 93.557(2), 102.459(2)
V (Å^3)	2017.7(2)
Z	1
Radiation type	Mo *K*α
μ (mm^{-1})	0.85
Crystal size (mm)	0.14 × 0.12 × 0.02
Data collection	
Diffractometer	Bruker Quest (PHOTON III)
Absorption correction	Multi-scan *SADABS2016*/2 (Bruker,2016/2)
T_{min}, T_{max}	0.297, 0.333
No. of measured, independent and observed [$I > 2\sigma(I)$] reflections	91704, 10239, 9089
R_{int}	0.035
$(\sin \theta/\lambda)_{max}$ (Å^{-1})	0.702
Refinement	
$R[F^2 > 2\sigma(F^2)]$, $wR(F^2)$, S	0.027, 0.058, 1.06
No. of reflections	10239
No. of parameters	531
No. of restraints	80
H-atom treatment	H-atom parameters constrained
$\Delta\rho_{max}$, $\Delta\rho_{min}$ (e Å^{-3})	0.80, −0.65

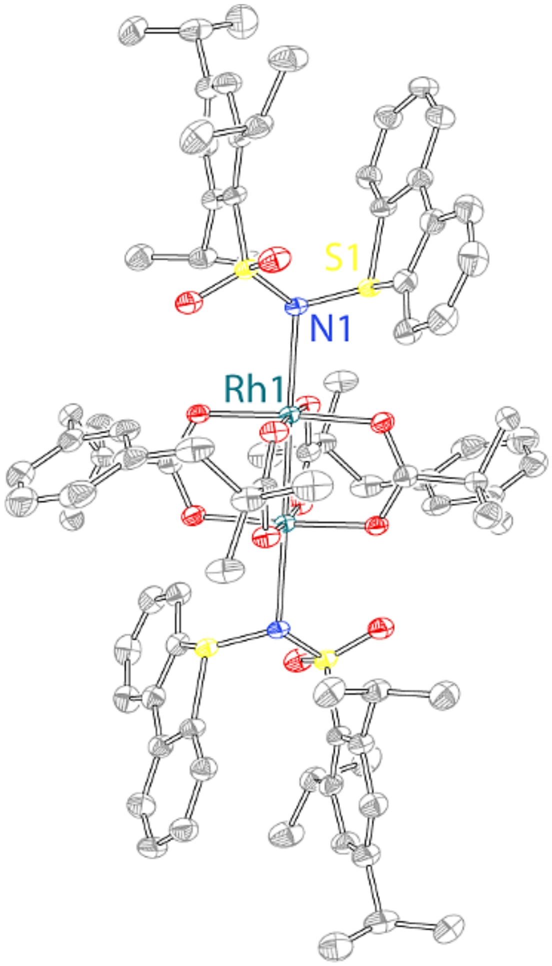

Figure S32. Displacement ellipsoid plot of **9d** plotted at 50% probability. H-atoms and solvents are removed for clarity. The crystalline sample used in this diffraction experiment was obtained from a concentrated CH_2Cl_2 solution at −20 °C.

Table S4. X-ray experimental details of **9d** (CCDC 2261078).

Crystal data	
Chemical formula	$C_{86}H_{102}N_2O_{12}Rh_2S_4$
M_r	1689.75
Crystal system, space group	Triclinic, *P*-1
Temperature (K)	110
a, *b*, *c* (Å)	13.044(1), 13.531(1), 14.145(1)
α, β, γ (°)	62.503(2), 69.314(2), 72.954(2)
V ($Å^3$)	2046.2(3)
Z	1
Radiation type	Synchrotron, λ = 0.41328 Å
μ (mm^{-1})	0.61
Crystal size (mm)	0.04 × 0.03 × 0.01
Data collection	
Diffractometer	Synchrotron
Absorption correction	Multi-scan *SADABS2016*/2 (Bruker,2016/2)
T_{min}, T_{max}	0.497, 0.744
No. of measured, independent and observed [$I > 2\sigma(I)$] reflections	53713, 8069, 6537
R_{int}	0.102
$(\sin \theta/\lambda)_{max}$ ($Å^{-1}$)	0.627
Refinement	
$R[F^2 > 2\sigma(F^2)]$, $wR(F^2)$, S	0.045, 0.117, 1.05
No. of reflections	8069
No. of parameters	488
H-atom treatment	H-atom parameters constrained
$\Delta\rho_{max}$, $\Delta\rho_{min}$ (e $Å^{-3}$)	1.10, −1.54

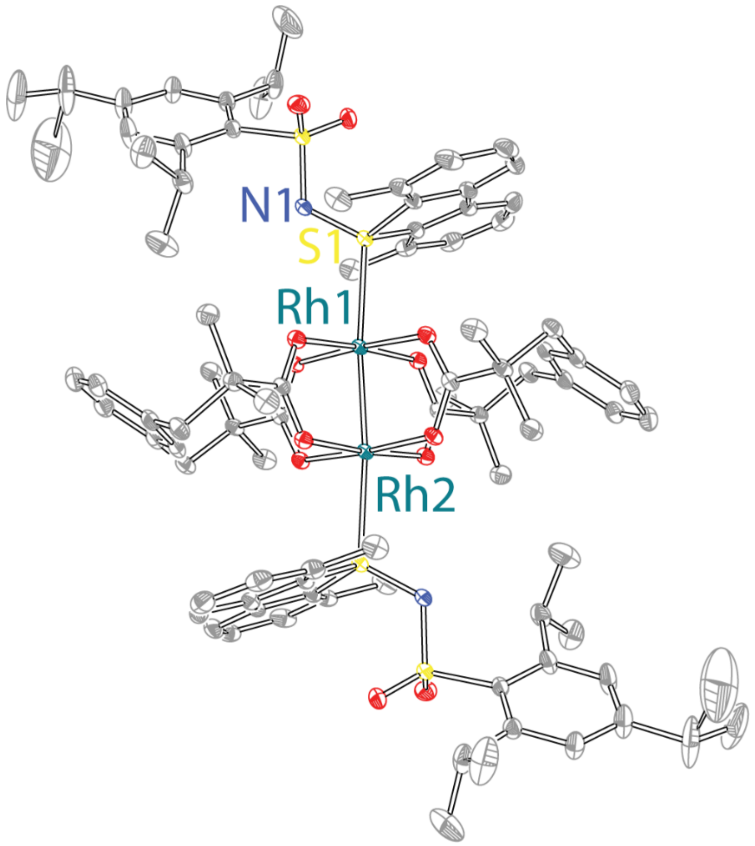

Figure S33. Displacement ellipsoid plot of **9e** plotted at 50% probability. H-atoms and solvents are removed for clarity. The crystalline sample used in this diffraction experiment was obtained from a concentrated CH_2Cl_2 solution at −20 °C.

Table S5. X-ray experimental details of **9e** (CCDC 2261073).

Crystal data	
Chemical formula	$C_{90}H_{110}N_2O_{12}Rh_2S_4$
M_r	1745.85
Crystal system, space group	Monoclinic, $P2_1/c$
Temperature (K)	110
a, b, c (Å)	15.610(1), 11.014(8), 26.605(2)
β (°)	95.679(2)
V (Å^3)	4551.7(5)
Z	2
Radiation type	Mo $K\alpha$
μ (mm^{-1})	0.51
Crystal size (mm)	0.10 × 0.08 × 0.03
Data collection	
Diffractometer	Bruker Quest (PHOTON III)
Absorption correction	Multi-scan *SADABS2016*/2 (Bruker,2016/2)
T_{min}, T_{max}	0.296, 0.337
No. of measured, independent and observed [$I > 2\sigma(I)$] reflections	158464, 13877, 11194
R_{int}	0.060
$(\sin \theta/\lambda)_{max}$ (Å^{-1})	0.714
Refinement	
$R[F^2 > 2\sigma(F^2)]$, $wR(F^2)$, S	0.037, 0.113, 1.11
No. of reflections	13877
No. of parameters	531
No. of restraints	118
H-atom treatment	H-atom parameters constrained
$\Delta\rho_{max}$, $\Delta\rho_{min}$ (e Å^{-3})	2.42, −0.48

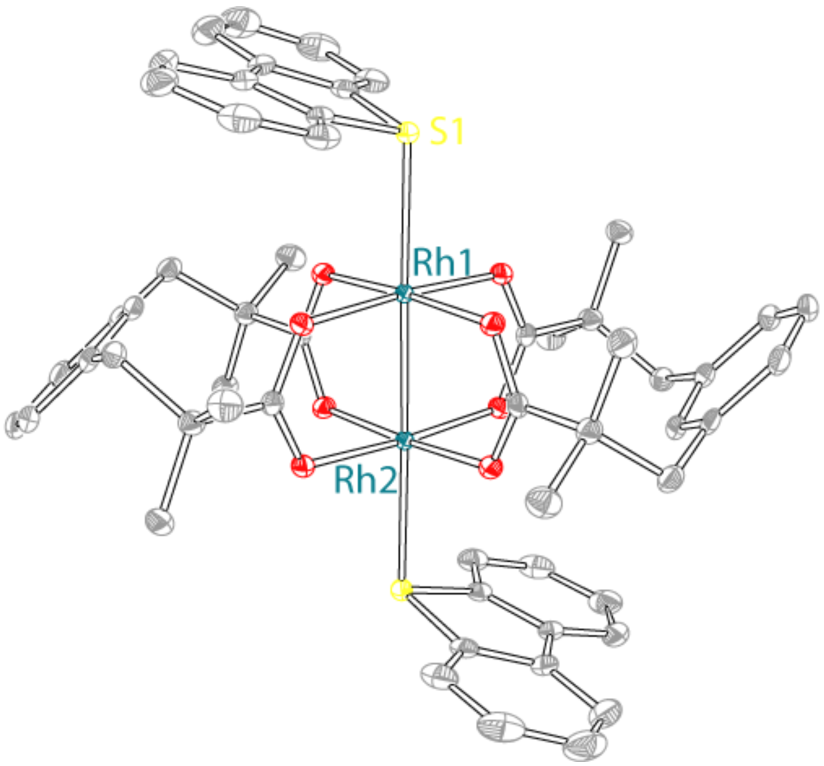

Figure S34. Displacement ellipsoid plot of $Rh_2esp_2(dbt)_2$ (**10**) plotted at 50% probability. H-atoms, and solvent are removed for clarity. The crystalline sample used in this diffraction experiment was obtained from a concentrated $CHCl_3$ solution at −20 °C.

Table S6. X-ray experimental details of $Rh_2esp_2(dbt)_2$ (**10**) (CCDC 2264679).

Crystal data	
Chemical formula	$C_{56}H_{56}O_8Rh_2S_2$
M_r	1126.94
Crystal system, space group	Triclinic, *P*-1
Temperature (K)	110
a, *b*, *c* (Å)	10.7223(8), 11.1208(9), 12.0976(9)
α, β, γ (°)	109.391(2), 111.669(2), 92.831(2)
V (Å^3)	1240.0(2)
Z	1
Radiation type	Mo *K*α
μ (mm^{-1})	0.81
Crystal size (mm)	0.16 × 0.14 × 0.03
Data collection	
Diffractometer	Bruker Quest (PHOTON III)
Absorption correction	Multi-scan *SADABS2016*/2 (Bruker,2016/2)
T_{min}, T_{max}	0.694, 0.745
No. of measured, independent and observed [$I > 2\sigma(I)$] reflections	25597, 6714, 5993
R_{int}	0.024
$(\sin \theta/\lambda)_{max}$ (Å^{-1})	0.718
Refinement	
$R[F^2 > 2\sigma(F^2)]$, $wR(F^2)$, S	0.023, 0.056, 1.03
No. of reflections	6714
No. of parameters	311
H-atom treatment	H-atom parameters constrained
$\Delta\rho_{max}$, $\Delta\rho_{min}$ (e Å^{-3})	0.47, −0.85

J. NMR Spectra

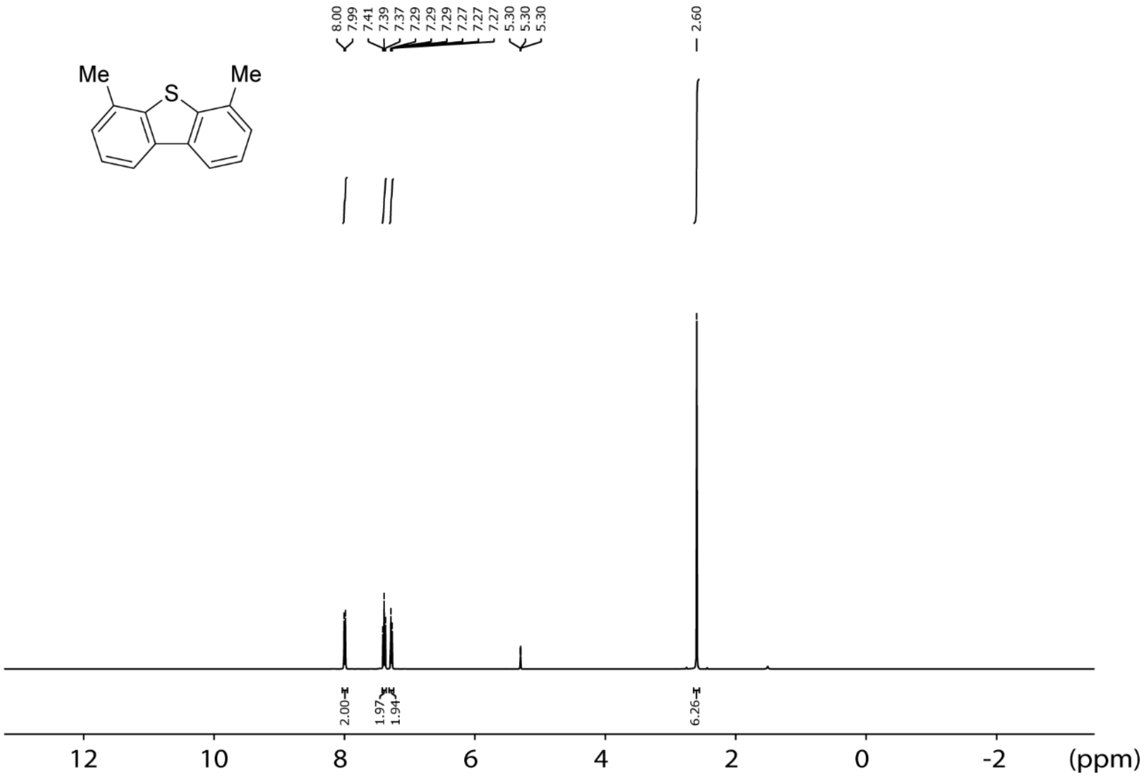

Figure S35. ^{1}H NMR spectrum of compound **2a** recorded in CD_2Cl_2 with an instrument operating at 400 MHz at 23 °C.

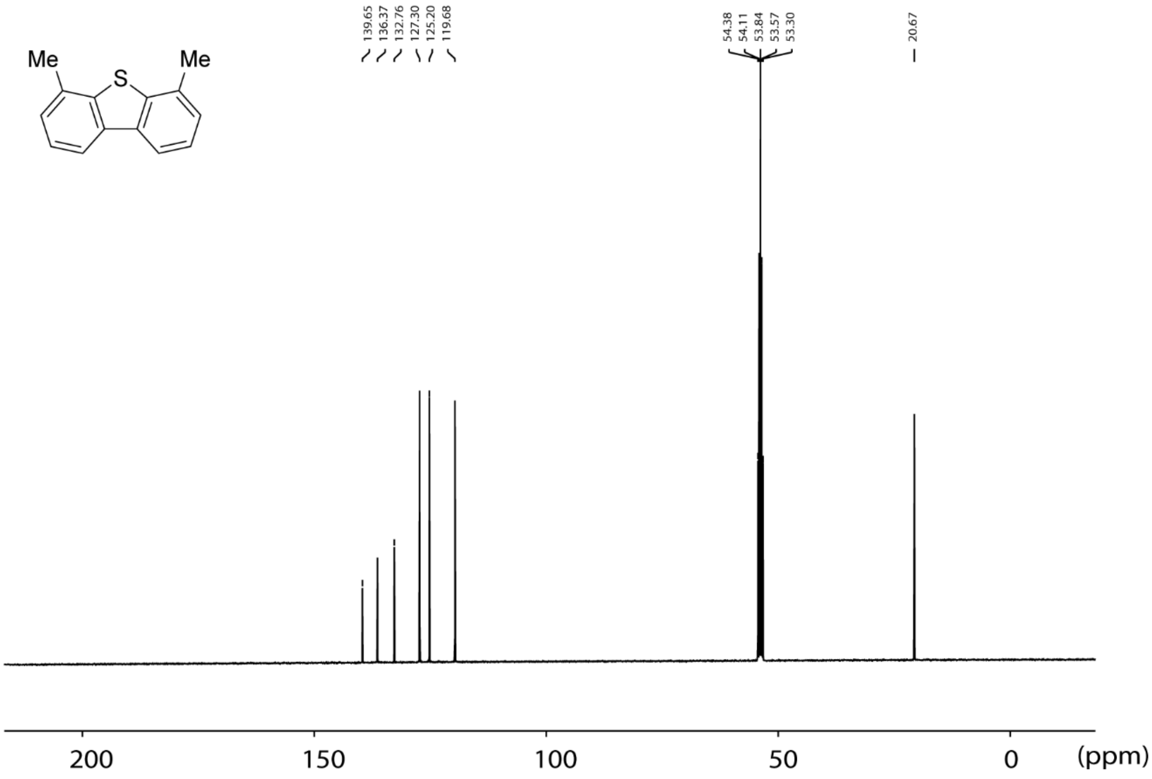

Figure S36. ^{13}C NMR spectrum of compound **2a** recorded in CD_2Cl_2 with an instrument operating at 125 MHz at 23 °C.

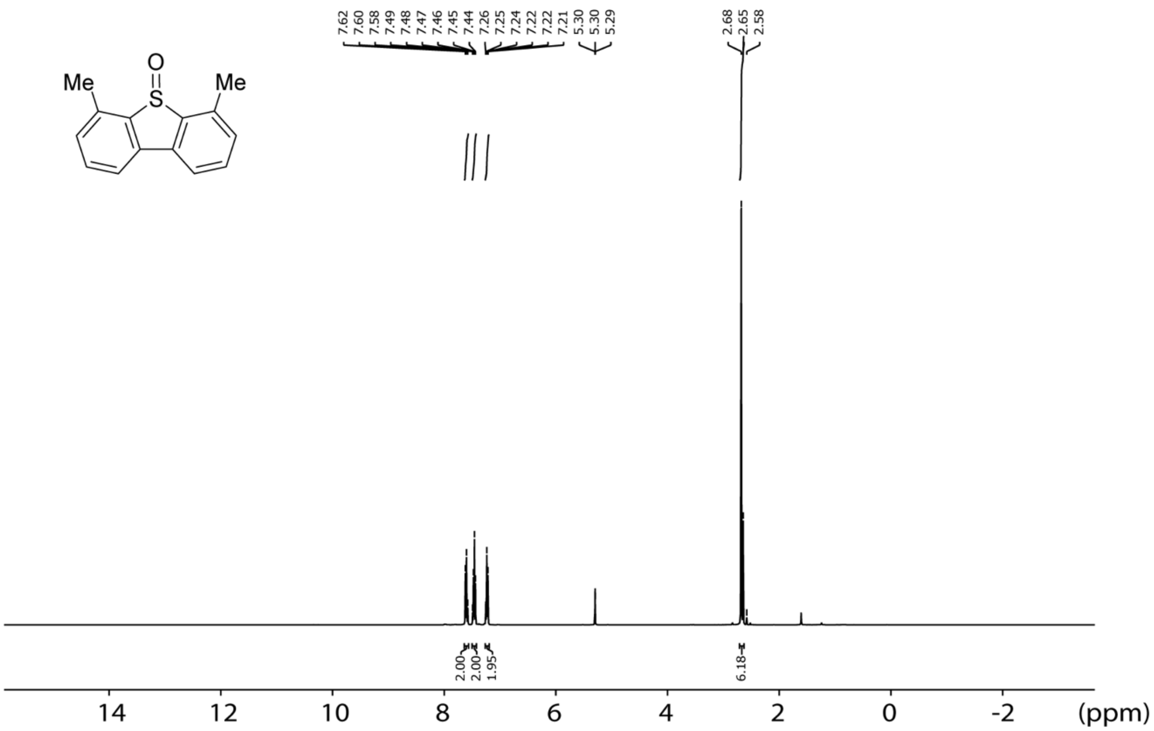

Figure S37. ^{1}H NMR spectrum of compound **3b** recorded in CD_2Cl_2 with an instrument operating at 400 MHz at 23 °C.

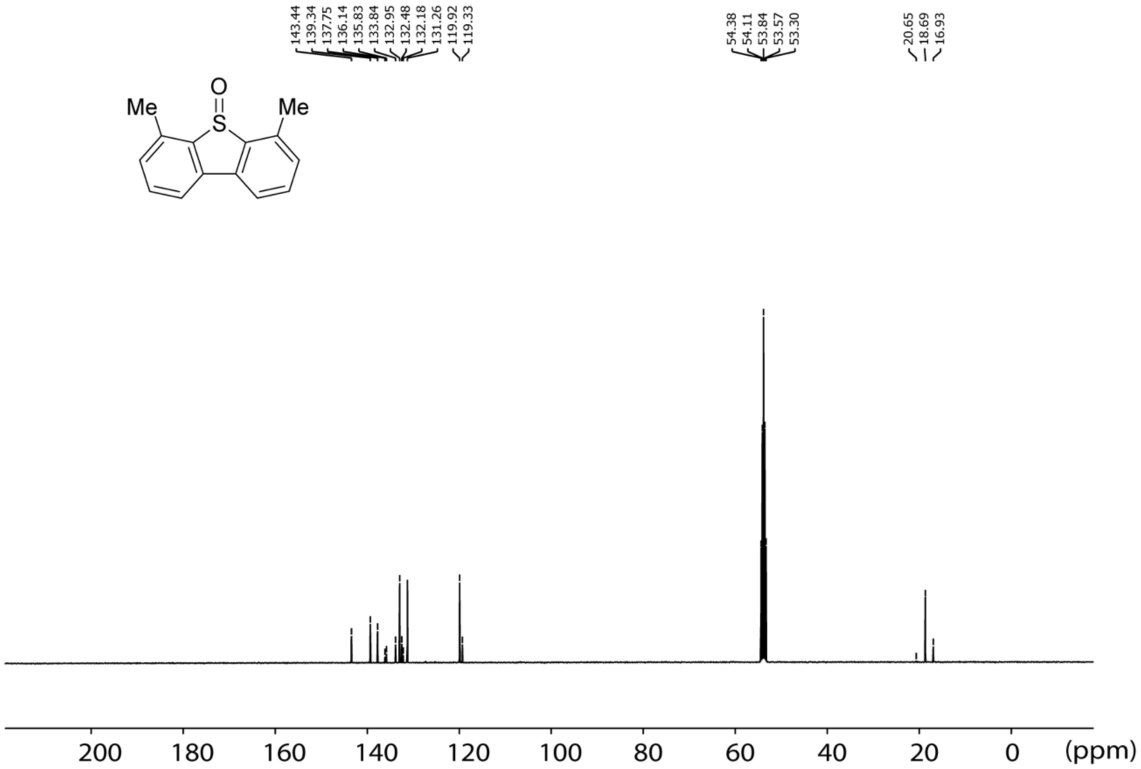

Figure S38. ^{13}C NMR spectrum of compound **3b** recorded in CD_2Cl_2 with an instrument operating at 125 MHz at 23 °C.

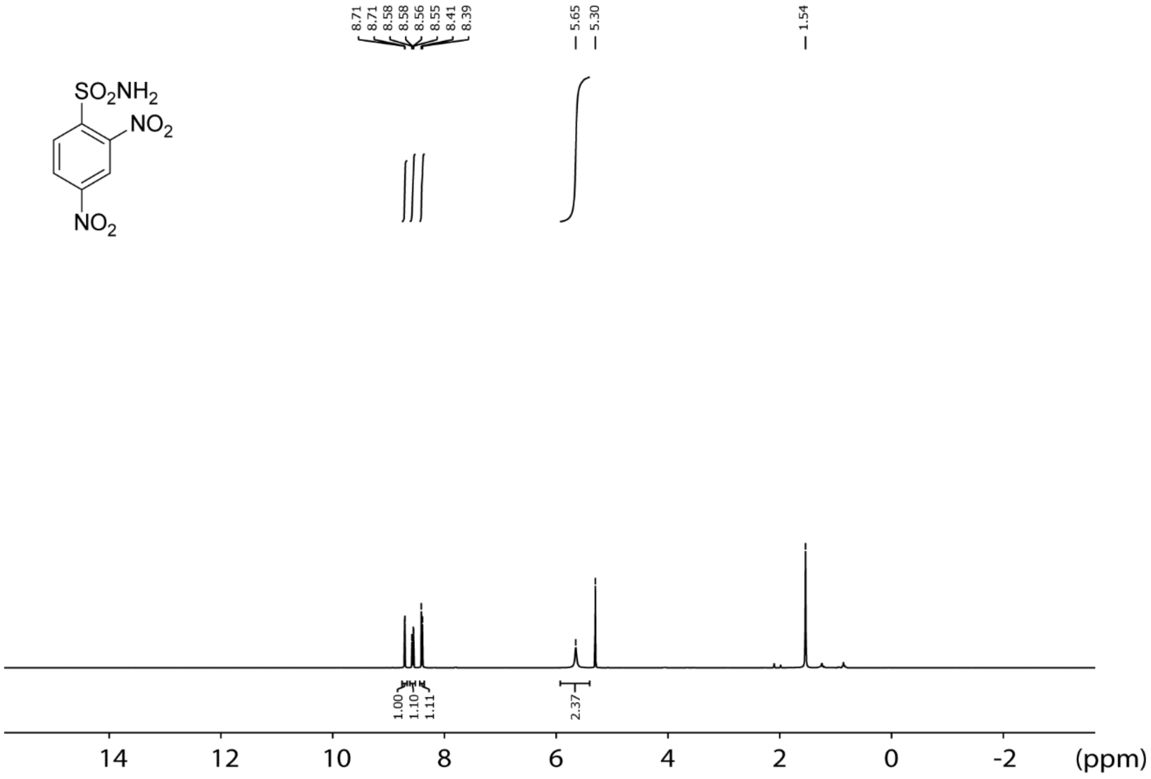

Figure S39. ^{1}H NMR spectrum of compound **4a** recorded in CD_2Cl_2 with an instrument operating at 400 MHz at 23 °C.

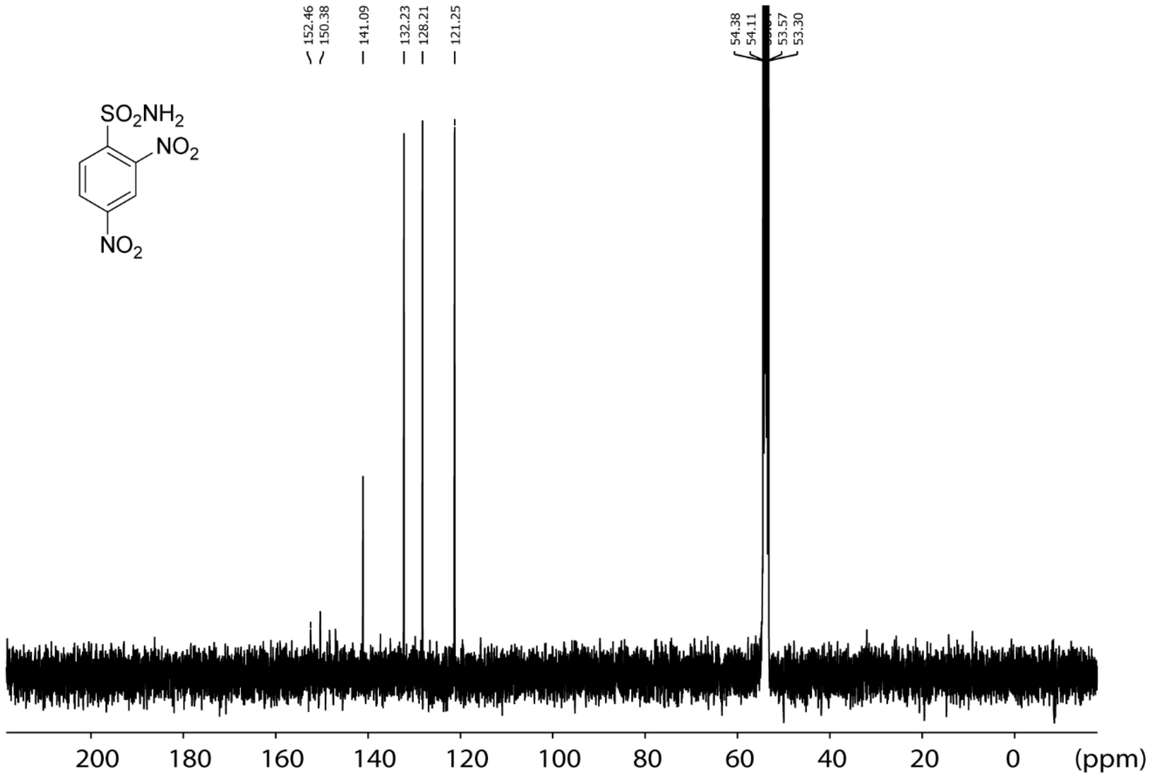

Figure S40. ^{13}C NMR spectrum of compound **4a** recorded in CD_2Cl_2 with an instrument operating at 125 MHz at 23 °C.

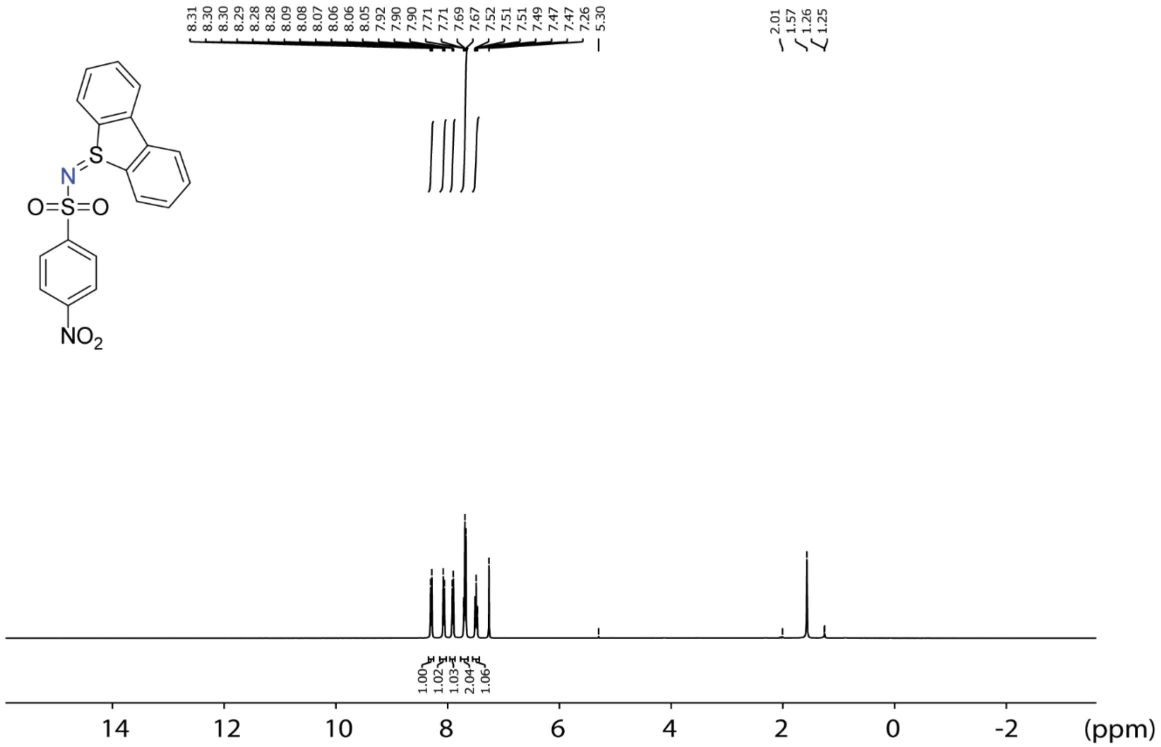

Figure S41. ^{1}H NMR spectrum of compound **6b** recorded in $CDCl_3$ with an instrument operating at 400 MHz at 23 °C.

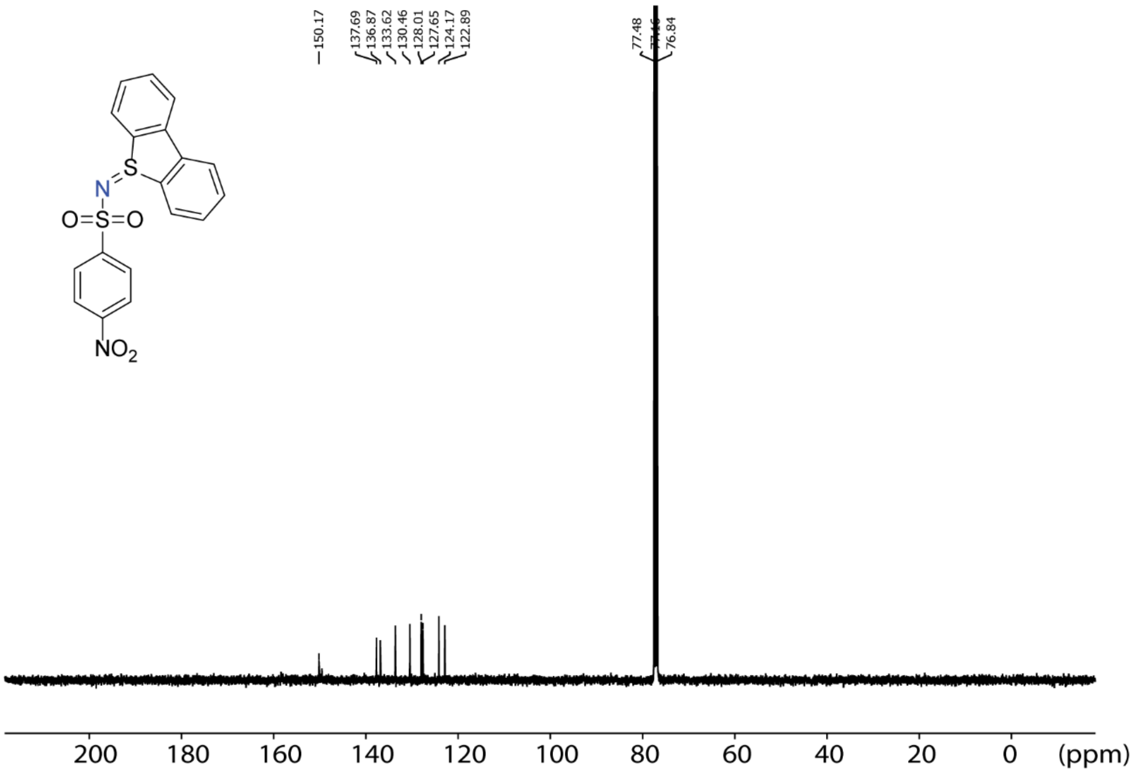

Figure S42. ^{13}C NMR spectrum of compound **6b** recorded in $CDCl_3$ with an instrument operating at 125 MHz at 23 °C.

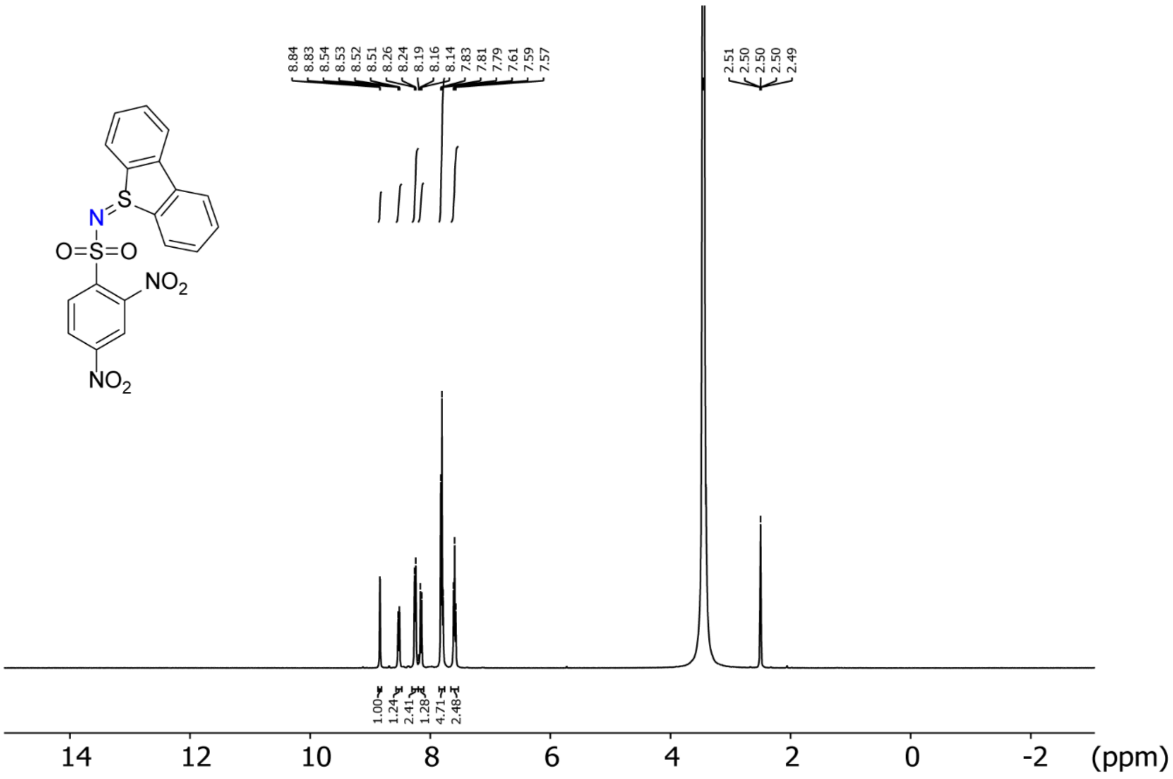

Figure S43. ^{1}H NMR spectrum of compound **6c** recorded in $(CD_3)_2SO$ with an instrument operating at 400 MHz at 23 °C.

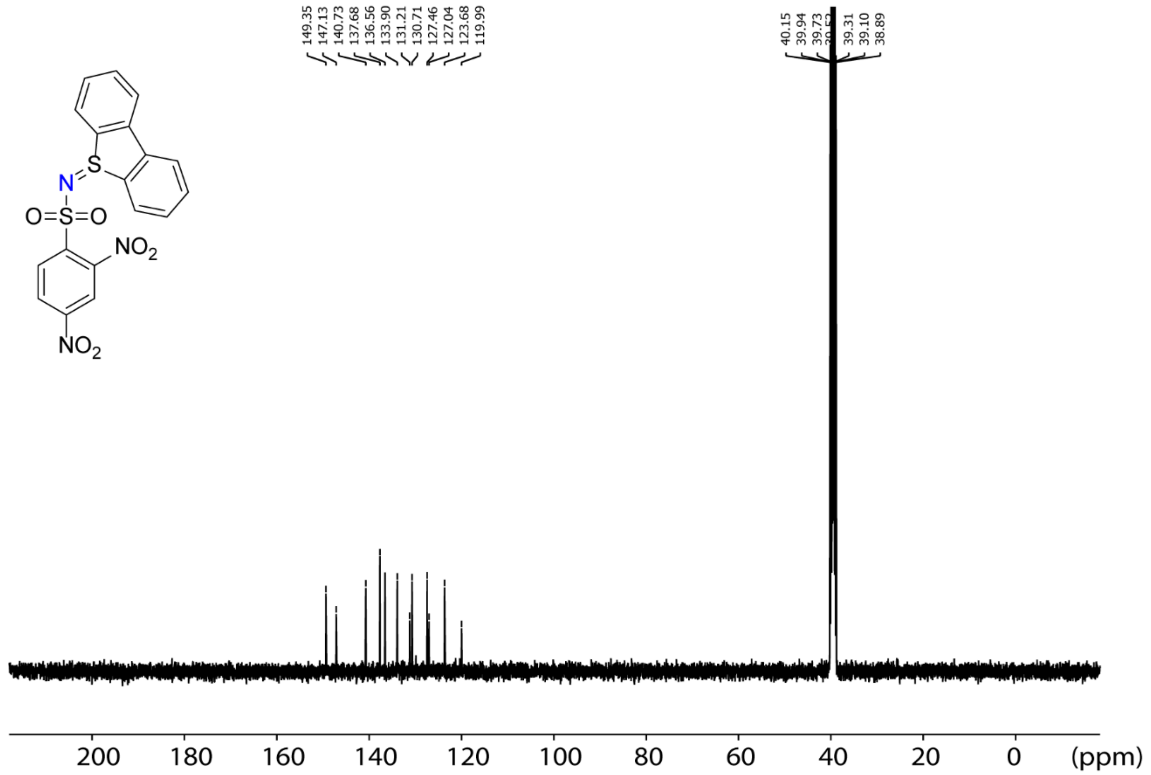

Figure S44. ^{13}C NMR spectrum of compound **6c** recorded in $(CD_3)_2SO$ with an instrument operating at 125 MHz at 23 °C.

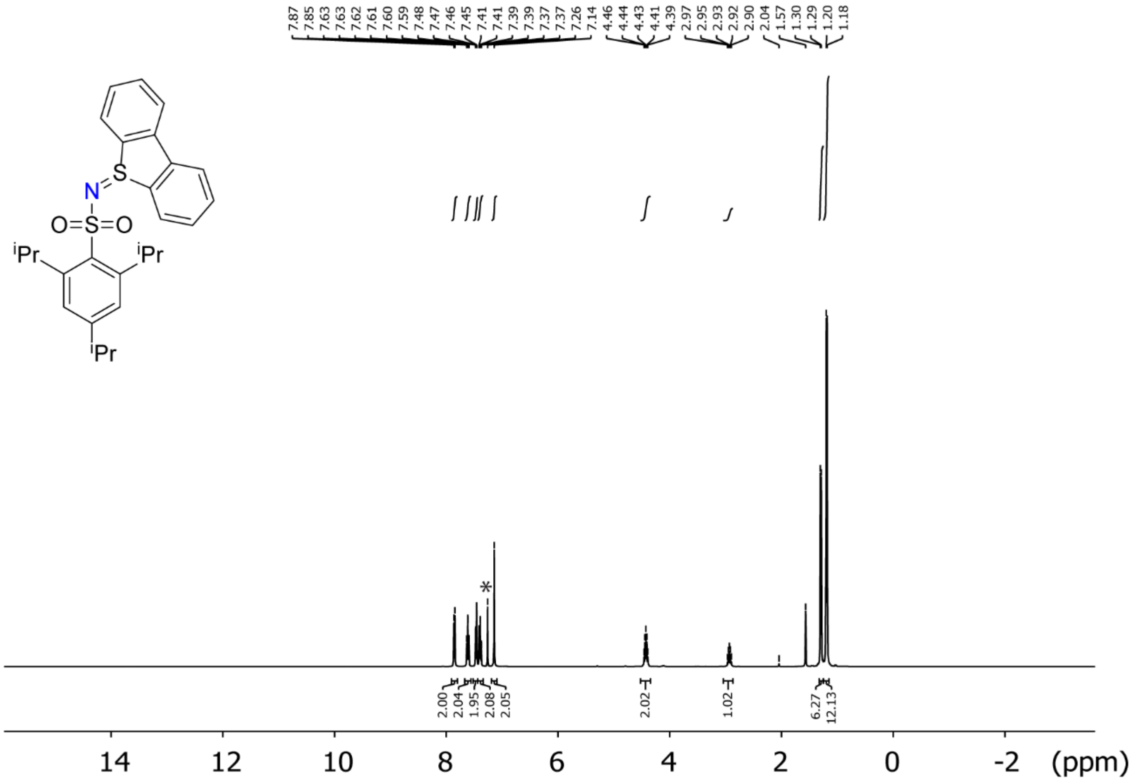

Figure S45. ^{1}H NMR spectrum of compound **6d** recorded in $CDCl_3$ with an instrument operating at 400 MHz at 23 °C.

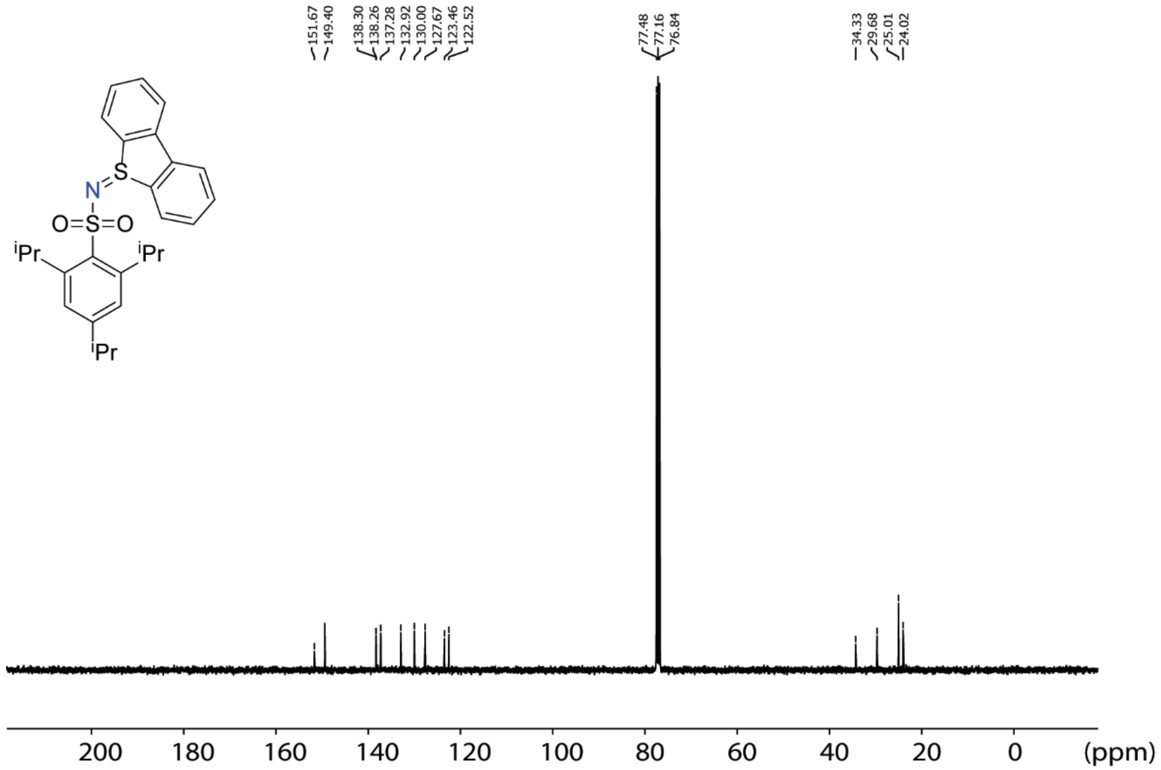

Figure S46. ^{13}C NMR spectrum of compound **6d** recorded in $CDCl_3$ with an instrument operating at 125 MHz at 23 °C.

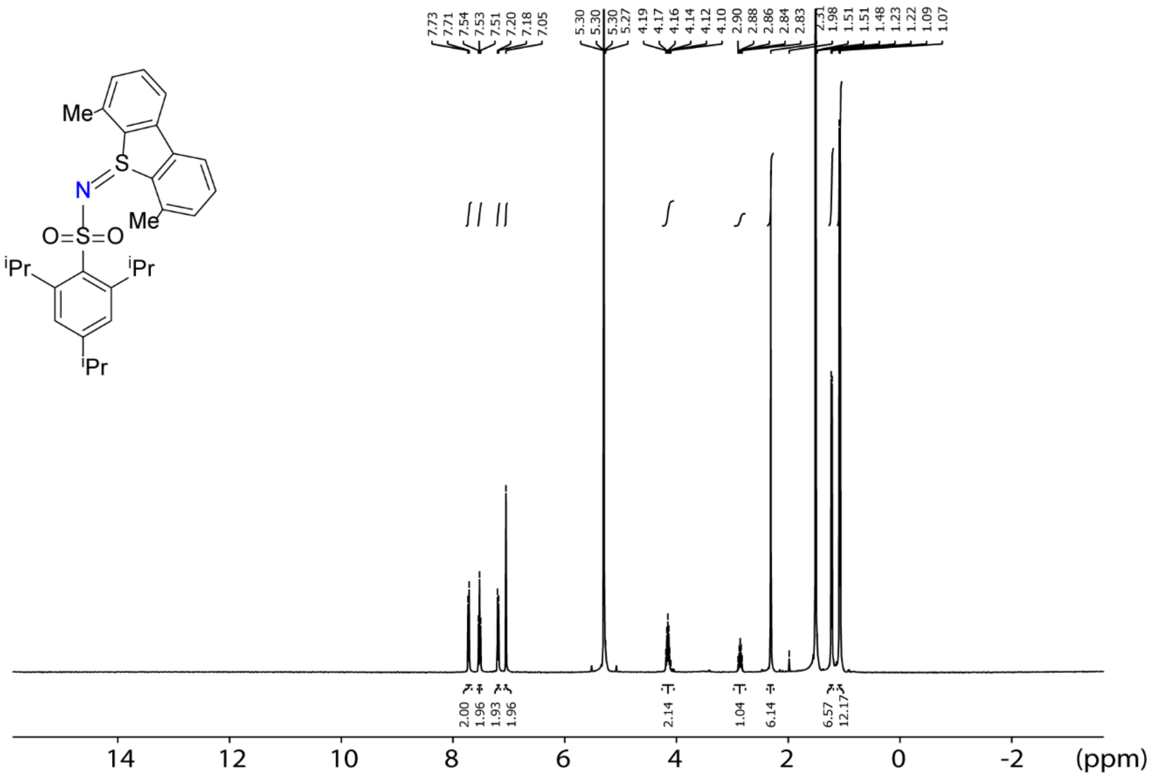

Figure S47. ^{1}H NMR spectrum of compound **6e** recorded in CD_2Cl_2 with an instrument operating at 400 MHz at 23 °C.

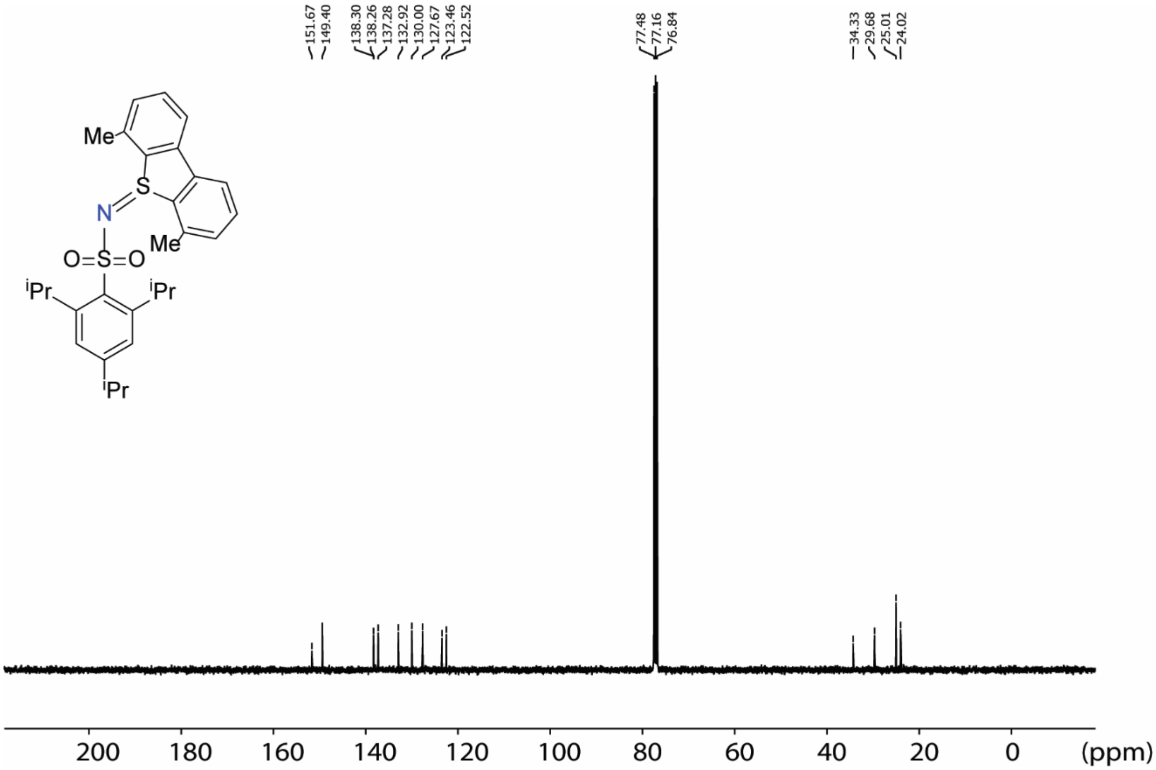

Figure S48. ^{13}C NMR spectrum of compound **6e** recorded in CD_2Cl_2 with an instrument operating at 125 MHz at 23 °C.

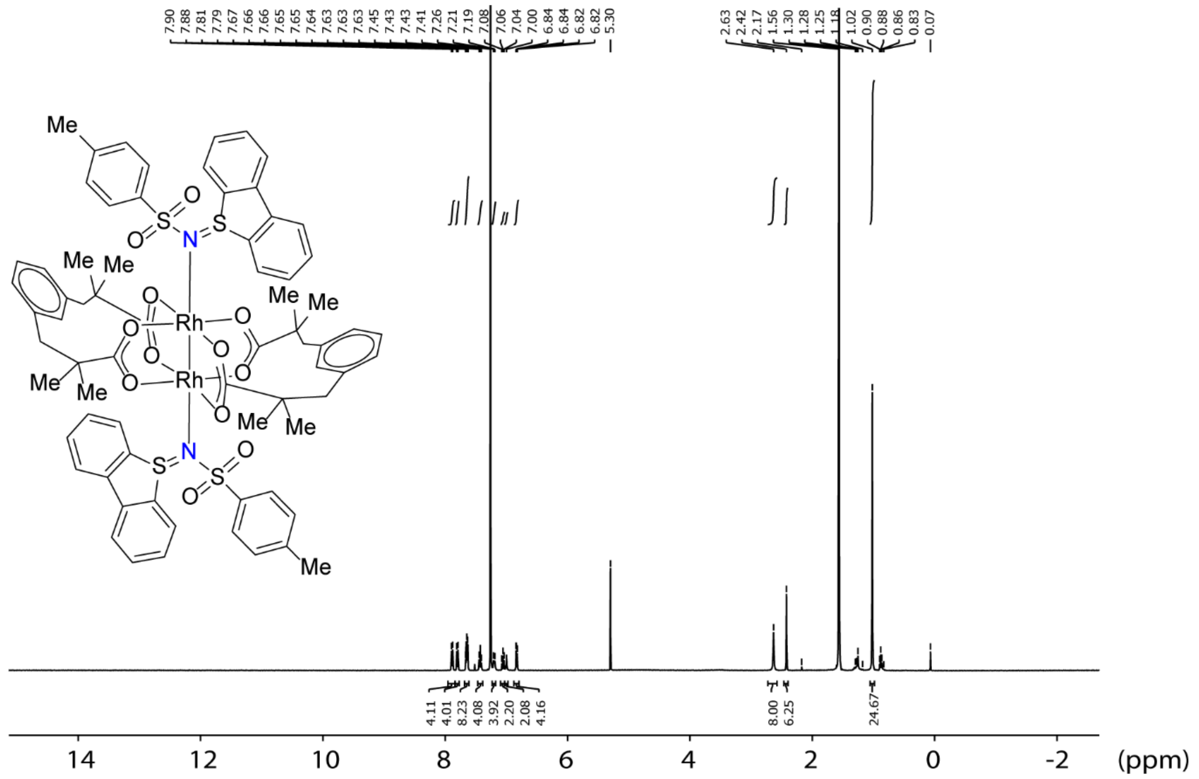

Figure S49. ^{1}H NMR spectrum of compound **9a** recorded in $CDCl_3$ with an instrument operating at 400 MHz at 23 °C.

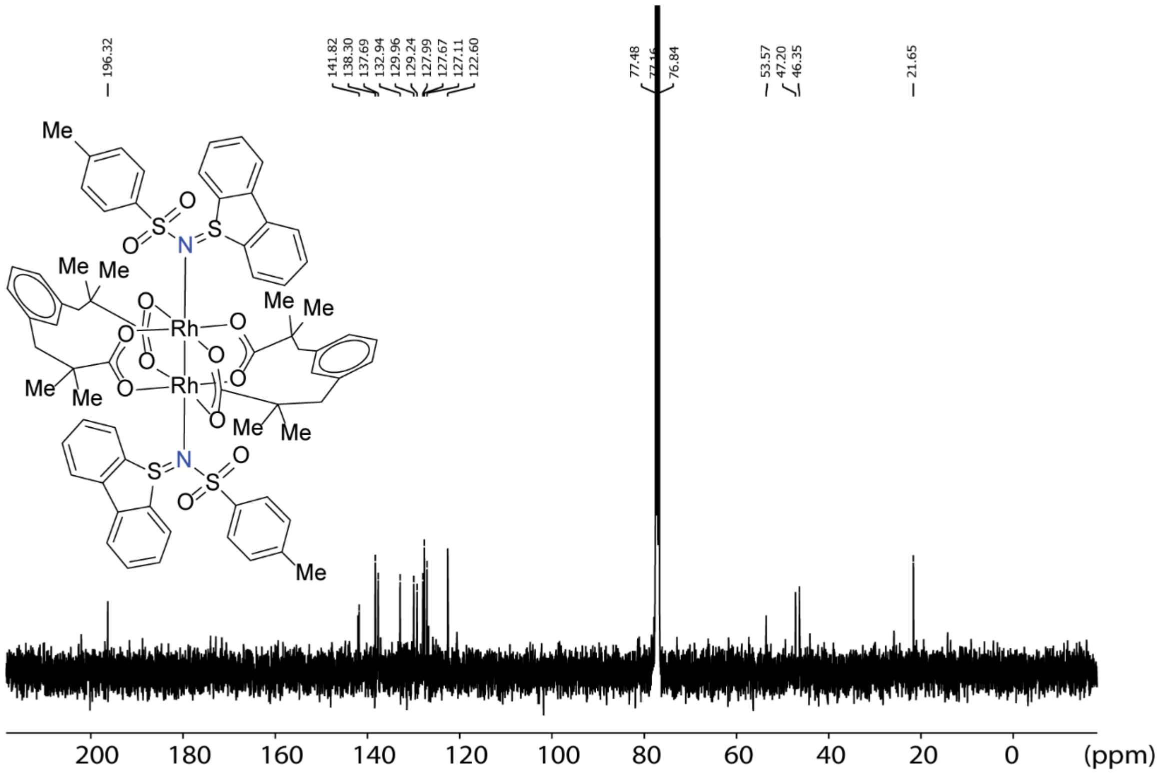

Figure S50. ^{13}C NMR spectrum of compound **9a** recorded in $CDCl_3$ with an instrument operating at 125 MHz at 23 °C.

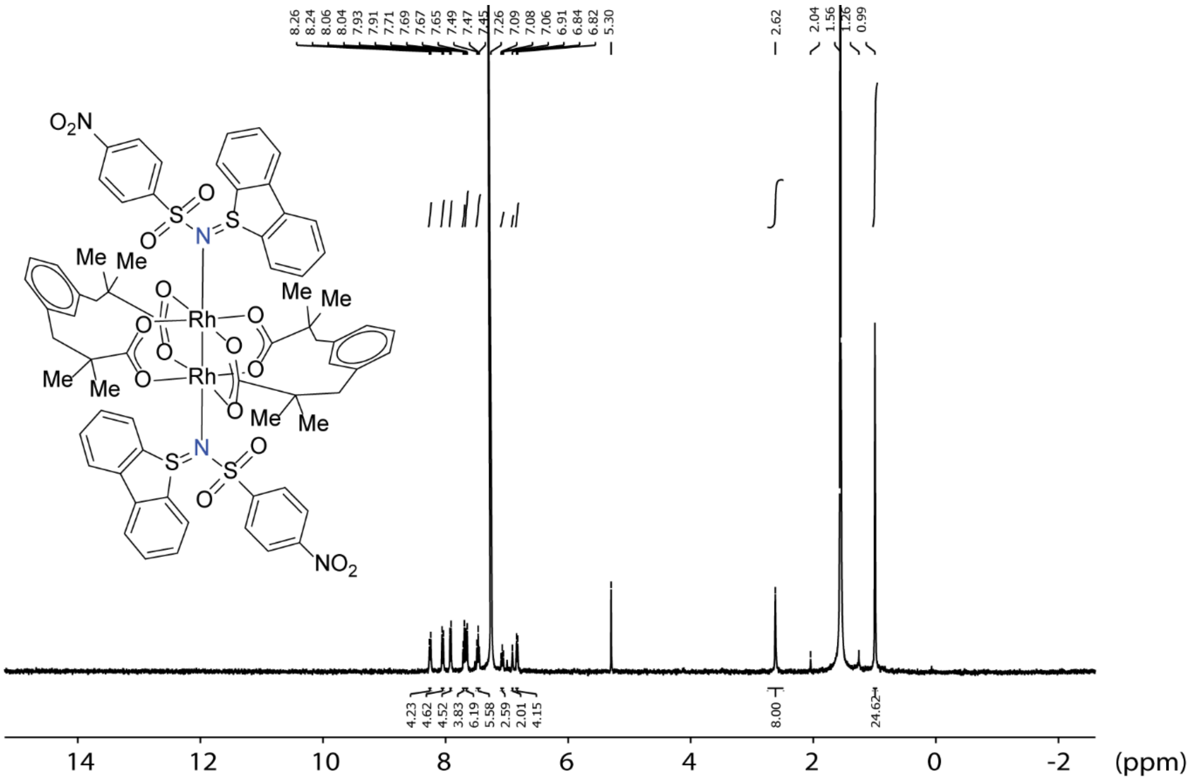

Figure S51. ^{1}H NMR spectrum of compound **9b** recorded in $CDCl_3$ with an instrument operating at 400 MHz at 23 °C.

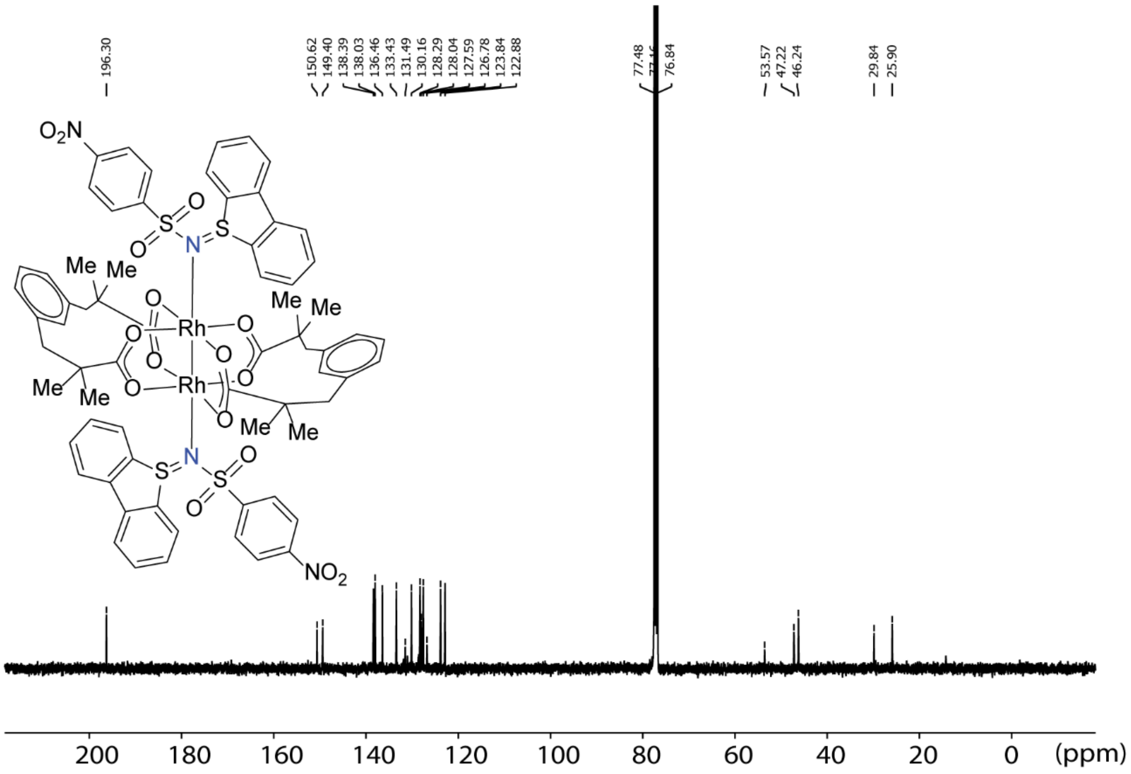

Figure S52. ^{13}C NMR spectrum of compound **9b** recorded in $CDCl_3$ with an instrument operating at 125 MHz at 23 °C.

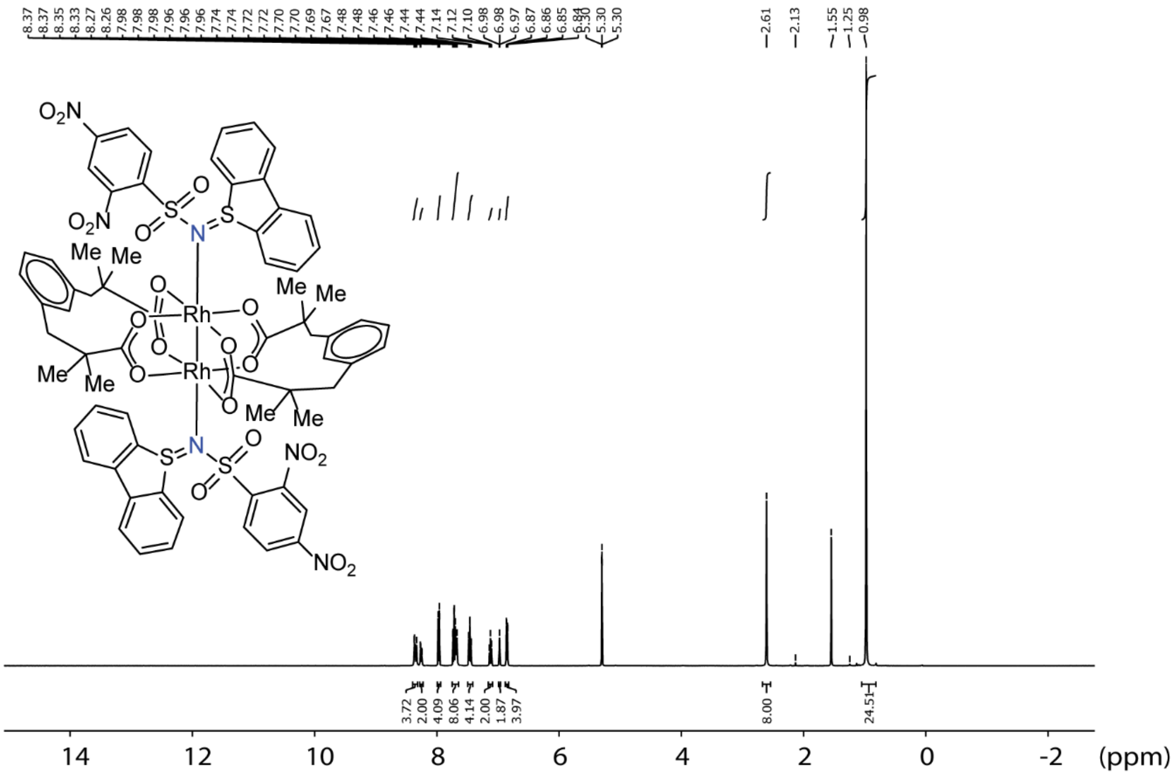

Figure S53. ^{1}H NMR spectrum of compound **9c** recorded in CD_2Cl_2 with an instrument operating at 400 MHz at 23 °C.

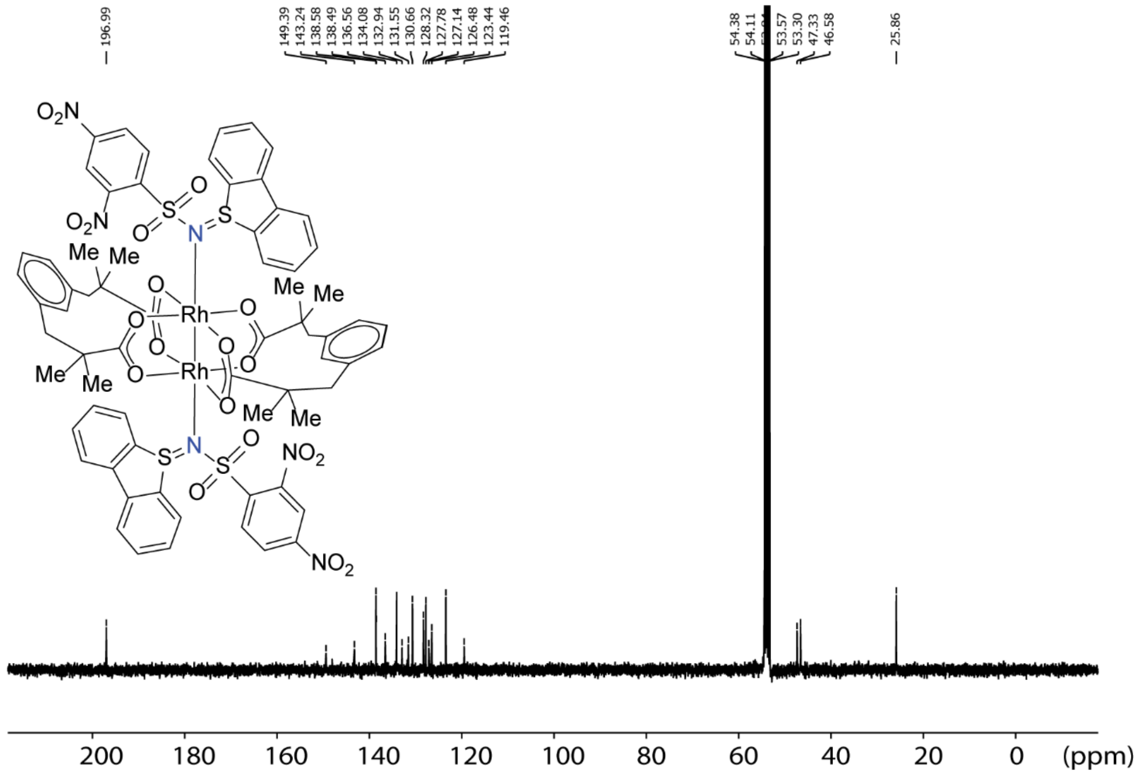

Figure S54. ^{13}C NMR spectrum of compound **9c** recorded in CD_2Cl_2 with an instrument operating at 125 MHz at 23 °C.

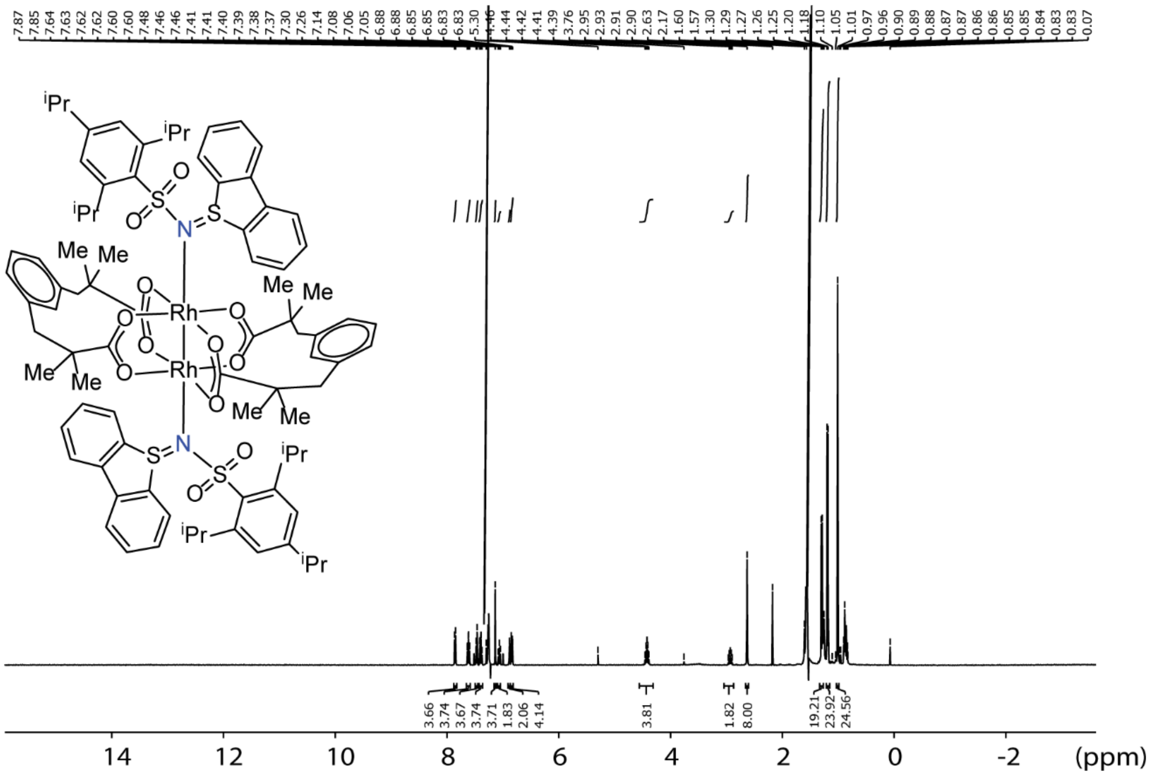

Figure S55. ^1H NMR spectrum of compound **9d** recorded in $CDCl_3$ with an instrument operating at 400 MHz at 23 °C.

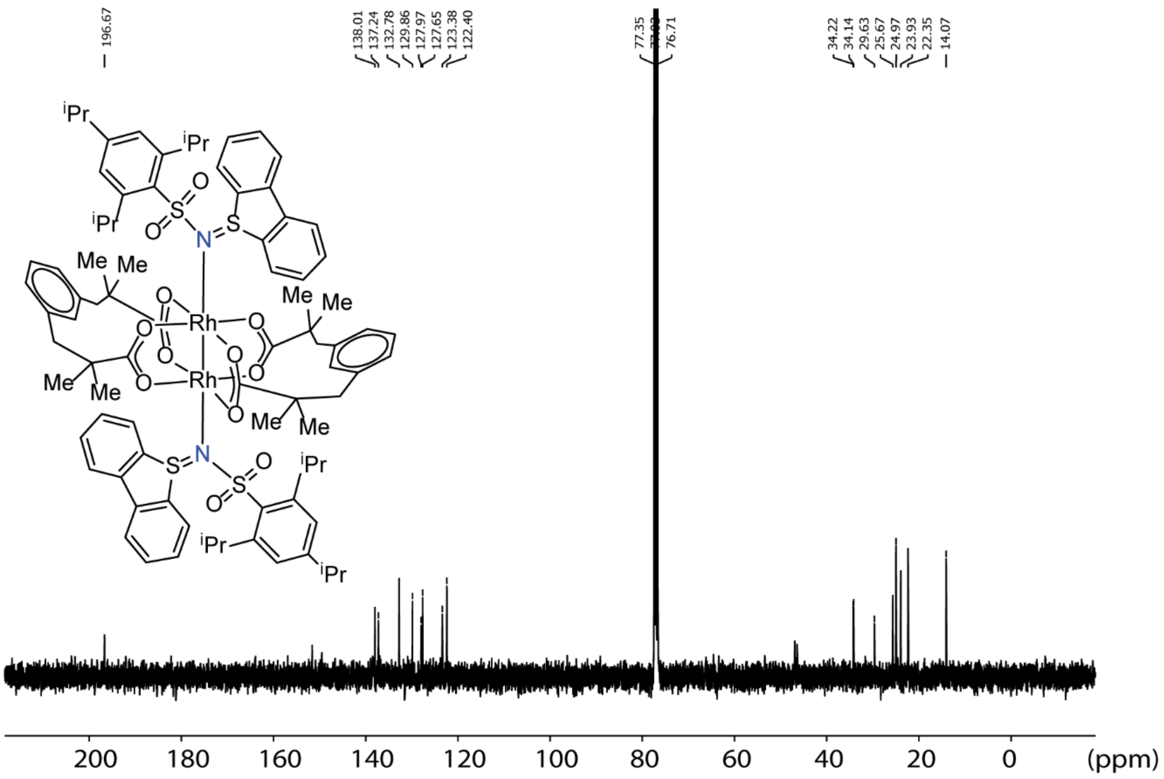

Figure S56. ^{13}C NMR spectrum of compound **9d** recorded in $CDCl_3$ with an instrument operating at 125 MHz at 23 °C.

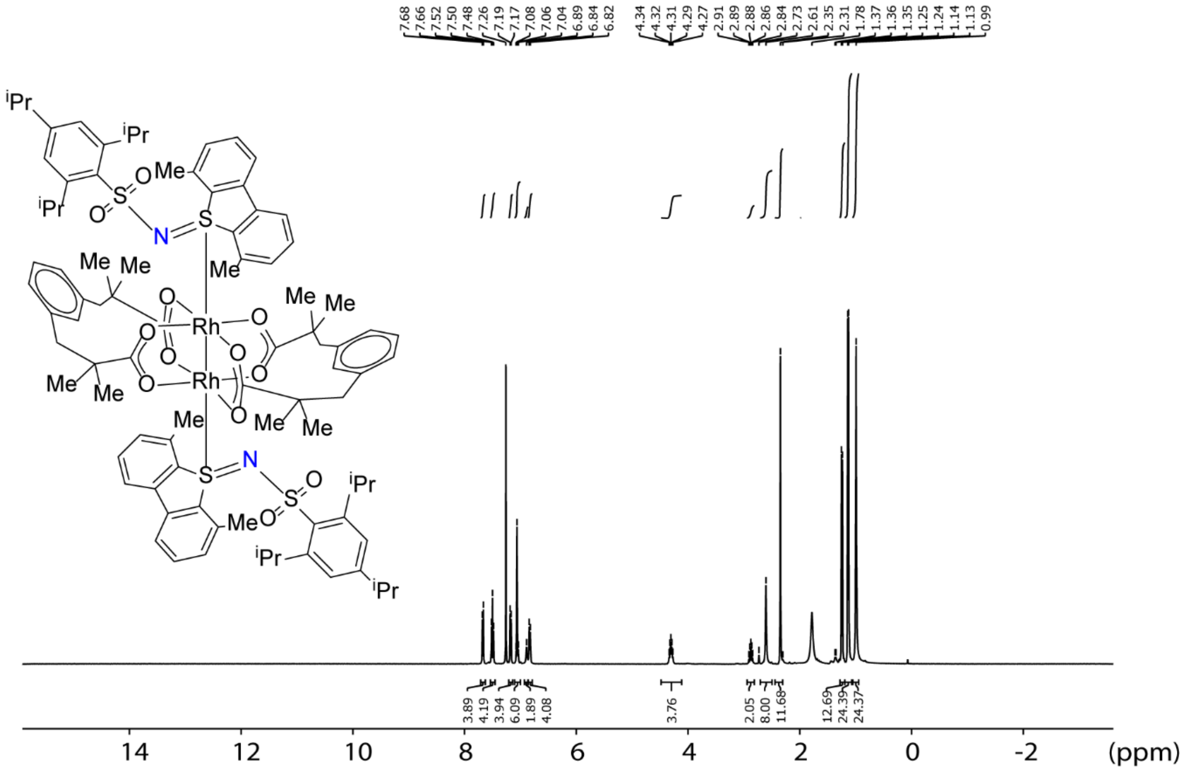

Figure S57. ^{1}H NMR spectrum of compound **9e** recorded in $CDCl_3$ with an instrument operating at 400 MHz at 23 °C.

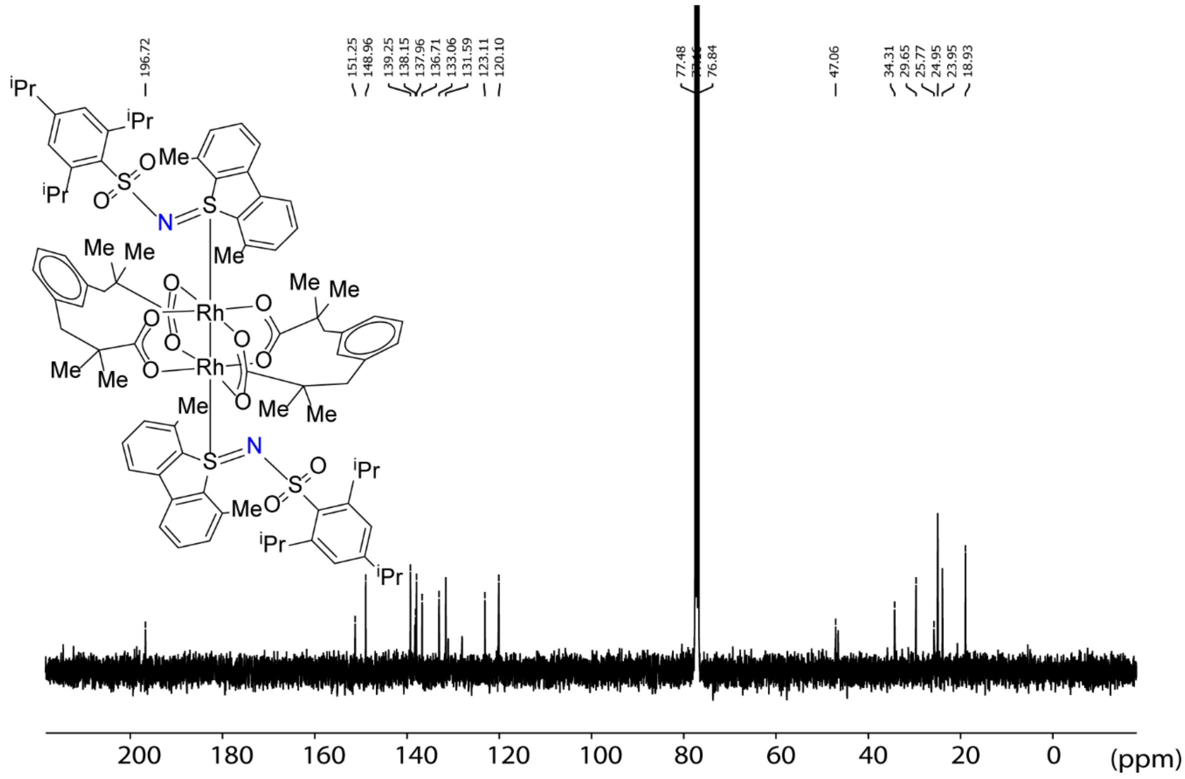

Figure S58. ^{13}C NMR spectrum of compound **9e** recorded in $CDCl_3$ with an instrument operating at 125 MHz at 23 °C.

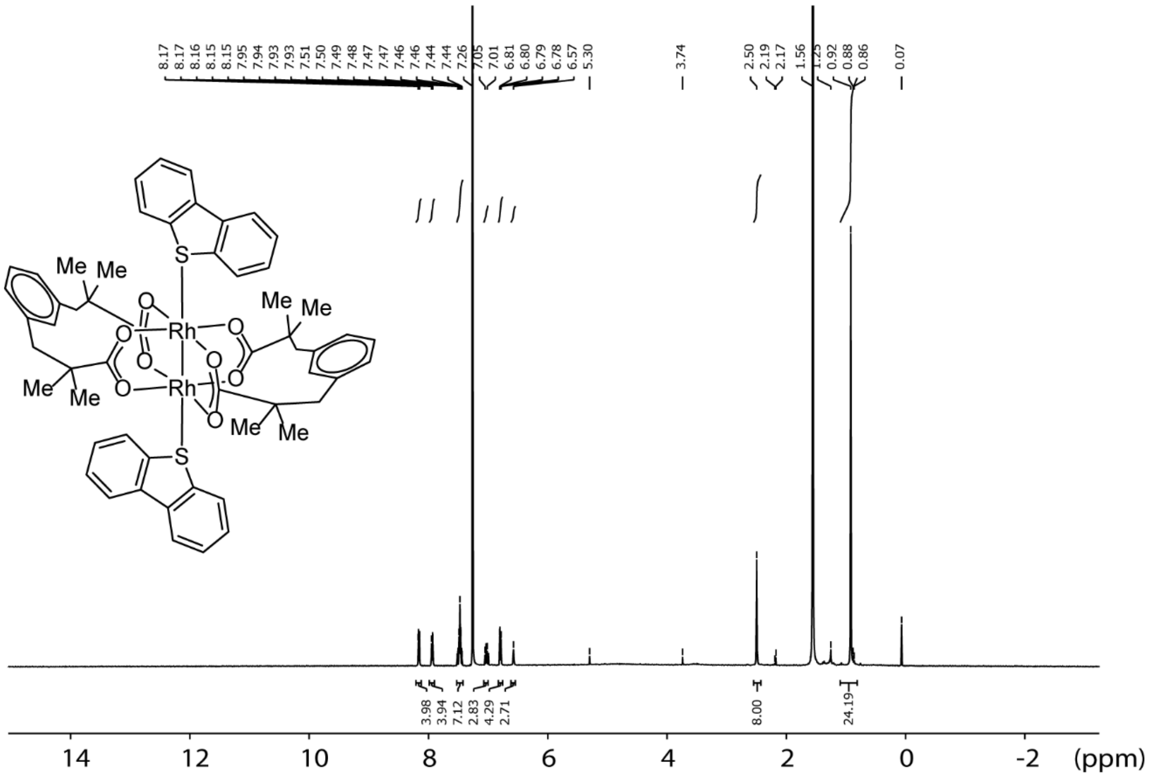

Figure S59. ^{1}H NMR spectrum of compound **10** recorded in $CDCl_3$ with an instrument operating at 400 MHz at 23 °C.

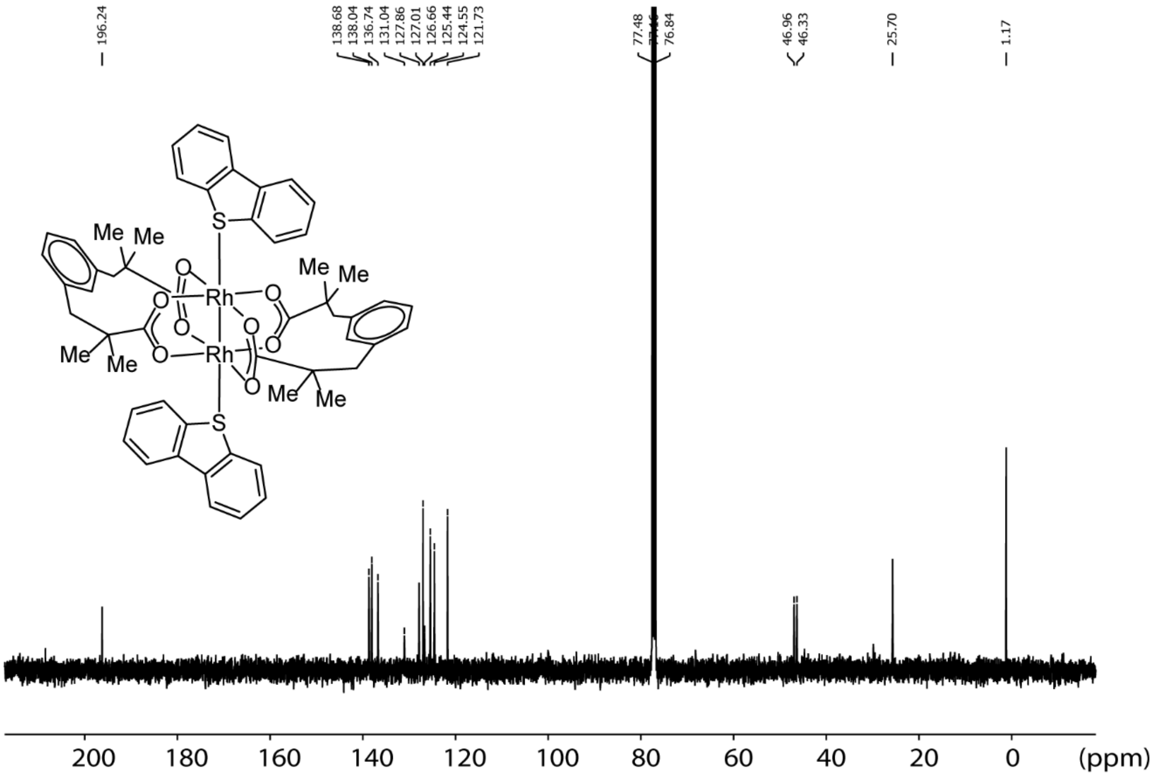

Figure S60. ^{13}C NMR spectrum of compound **10** recorded in $CDCl_3$ with an instrument operating at 125 MHz at 23 °C.

K. References

(1) Armarego, W. L. F.; Chai, C. L. L. Chapter 4 - Purification of Organic Chemicals. In *Purification of Laboratory Chemicals (Sixth Edition)*, Armarego, W. L. F., Chai, C. L. L. Eds.; Butterworth-Heinemann, 2009; pp 88–444.

(2) Pangborn, A. B.; Giardello, M. A.; Grubbs, R. H.; Rosen, R. K.; Timmers, F. J. Safe and Convenient Procedure for Solvent Purification. *Organometallics* **1996**, *15*, 1518–1520.

(3) Das, A.; Chen, Y.-S.; Reibenspies, J. H.; Powers, D. C. Characterization of a Reactive Rh2 Nitrenoid by Crystalline Matrix Isolation. *J. Am. Chem. Soc.* **2019**, *141*, 16232–16236.

(4) Fulmer, G. R.; Miller, A. J. M.; Sherden, N. H.; Gottlieb, H. E.; Nudelman, A.; Stoltz, B. M.; Bercaw, J. E.; Goldberg, K. I. NMR Chemical Shifts of Trace Impurities: Common Laboratory Solvents, Organics, and Gases in Deuterated Solvents Relevant to the Organometallic Chemist. *Organometallics* **2010**, *29*, 2176–2179.

(5) Dolomanov, O. V.; Bourhis, L. J.; Gildea, R. J.; Howard, J. A. K.; Puschmann, H. OLEX2: a complete structure solution, refinement and analysis program. *J. Appl. Cryst.* **2009**, *42*, 339–341.

(6) Sheldrick, G. A short history of SHELX. *Acta. Cryst. A* **2008**, *64*, 1121–1122. DOI: doi:10.1107/S0108767307043930. Sheldrick, G. Crystal structure refinement with SHELXL. *Acta. Cryst. C* **2015**, *71*, 3–8.

(7) Antoni, P. W.; Mackenroth, A. V.; Mulks, F. F.; Rudolph, M.; Helmchen, G.; Hashmi, A. S. K. Dibenzothiophenesulfilimines: A Convenient Approach to Intermolecular Rhodium-Catalysed C−H Amidation. *Chem. Eur. J.* **2020**, *26*, 8235–8238.

(8) Kuehm-Caubère, C.; Adach-Becker, S.; Fort, Y.; Caubère, P. Expeditious and efficient syntheses of pure 4-methyl and 4,6-disubstituted dibenzothiophenes. *Tetrahedron* **1996**, *52*, 9087–9092.

(9) Liu, R.; Zhang, W.; Wei, D.; Chen, J.-H.; Ng, S. W.; Yang, G. Adducts of triangular silver(i) 3,5-bis(trifluoromethyl)pyrazolate with thiophene derivatives: a weak interaction model of desulfurization. *Dalton Trans.* **2019**, *48*, 16162–16166.

(10) Gaitonde, V.; Sucheck, S. J. Synthesis of β-Glycosyl Amides from N-Glycosyl Dinitrobenzenesulfonamides. *J. Carbohydr. Chem.* **2012**, *31*, 353–370.

(11) Hayashi, T.; Kawai, M.; Tokunaga, N. Asymmetric Synthesis of Diarylmethyl Amines by Rhodium-Catalyzed Asymmetric Addition of Aryl Titanium Reagents to Imines. *Angew. Chem. Int. Ed.* **2004**, *43*, 6125–6128.

(12) Ding, Y.; Zhang, S.-Y.; Chen, Y.-C.; Fan, S.-X.; Tian, J.-S.; Loh, T.-P. Regioselective C–H Amidation of (Alkyl)arenes by Iron(II) Catalysis. *Org. Let.* **2019**, *21*, 2736–2739.

(13) Davis, O. A.; Croft, R. A.; Bull, J. A. Synthesis of diversely functionalised 2,2-disubstituted oxetanes: fragment motifs in new chemical space. *Chem. Commun.* **2015**, *51*, 15446–15449.

(14) Diethelm, S.; Schindler, C. S.; Carreira, E. M. Access to the Aeruginosin Serine Protease Inhibitors through the Nucleophilic Opening of an Oxabicyclo[2.2.1]heptane: Total Synthesis of Microcin SF608. *Chem. Eur. J.* **2014**, *20*, 6071–6080.

(15) Luo, L.; Tang, J.; Sun, R.; Li, W.; Zheng, X.; Yuan, M.; Li, R.; Chen, H.; Fu, H. Direct C–H Sulfonylimination of Pyridinium Salts. *Org. Let.* **2022**, *24*, 2821–2825.
